# Determinants of the Efficacy of Cardiac Ischemic Preconditioning: A Systematic Review and Meta-Analysis of Animal Studies

**DOI:** 10.1371/journal.pone.0142021

**Published:** 2015-11-18

**Authors:** Kimberley E. Wever, Carlijn R. Hooijmans, Niels P. Riksen, Thomas B. Sterenborg, Emily S. Sena, Merel Ritskes-Hoitinga, Michiel C. Warlé

**Affiliations:** 1 Systematic Review Centre for Laboratory animal Experimentation (SYRCLE), Radboud University Medical Center, Nijmegen, The Netherlands; 2 Department of Internal Medicine and Dept. of Pharmacology-Toxicology, Radboud University Medical Center, Nijmegen, The Netherlands; 3 Department of Surgery, Radboud University Medical Center, Nijmegen, The Netherlands; 4 Centre for Clinical Brain Sciences, University of Edinburgh, Edinburgh, United Kingdom; University of Colorado Denver, UNITED STATES

## Abstract

**Background:**

Ischemic preconditioning (IPC) of the heart is a protective strategy in which a brief ischemic stimulus immediately before a lethal ischemic episode potently limits infarct size. Although very promising in animal models of myocardial infarction, IPC has not yet been successfully translated to benefit for patients.

**Objective:**

To appraise all preclinical evidence on IPC for myocardial infarction and identify factors hampering translation.

**Methods and results:**

Using systematic review and meta-analysis, we identified 503 animal studies reporting infarct size data from 785 comparisons between IPC-treated and control animals. Overall, IPC reduced myocardial infarction by 24.6% [95%CI 23.5, 25.6]. Subgroup analysis showed that IPC efficacy was reduced in comorbid animals and non-rodents. Efficacy was highest in studies using 2–3 IPC cycles applied <45 minutes before myocardial infarction. Local and remote IPC were equally effective. Reporting of study quality indicators was low: randomization, blinding and a sample size calculation were reported in 49%, 11% and 2% of publications, respectively.

**Conclusions:**

Translation of IPC to the clinical setting may be hampered by the observed differences between the animals used in preclinical IPC studies and the patient population, regarding comorbidity, sex and age. Furthermore, the IPC protocols currently used in clinical trials could be optimized in terms of timing and the number of ischemic cycles applied. In order to inform future clinical trials successfully, future preclinical studies on IPC should aim to maximize both internal and external validity, since poor methodological quality may limit the value of the preclinical evidence.

## Introduction

Coronary heart disease is projected to remain the leading cause of disability and death worldwide until 2030[1,2]. Despite optimal reperfusion strategies, mortality and morbidity in patients with acute coronary syndrome remain significant. This is caused, at least in part, by ‘lethal reperfusion injury’[3]. Therefore, novel strategies to reduce myocardial ischemia-reperfusion injury (IRI) are urgently needed.

Ischemic preconditioning (IPC) is a protective strategy where brief sublethal bursts of ischemia and reperfusion induce protection against a subsequent prolonged ischemic insult. Its protective effects have been demonstrated in hundreds of experiments in rodents, dogs and pigs. In the last decade, over thirty clinical trials have investigated the protective effects of remote IPC (RIPC) in patients undergoing major cardiovascular surgery [4]. Compared to the preceding animal studies, the results have been disappointing: the most extensive meta-analysis of remote ischemic conditioning in cardiovascular surgery indicated that 11 out of 15 trials found no effect of RIPC on peak troponin levels [5]. Furthermore, four independent meta-analyses reached variable conclusions on the effect of RIPC on mortality, peri-operative myocardial infarction (MI) and major cardiovascular events [5–8]. Thus, the promising protective effect of ischemic conditioning in animal studies has not yet been translated to the clinical setting [9].

Translational failure is not unique to ischemic conditioning. Recent studies in other fields provide disquieting evidence regarding the limited predictive value of animal studies for therapeutic effects in humans. For translational research to be effective, animal models should be sufficiently similar to the human disease (external validity), and measures should be taken to minimize the risk of bias (internal validity), which may otherwise substantially confound the observed efficacy[10]. A lack of effective review of available animal data, as well as shortcomings in the design and reporting of animal studies, can severely hamper the translation of animal data to clinical practice[11,12].

Systematic review of the animal studies on IPC may provide insights into a number of issues currently impeding translation. For instance, there is no consensus on how many ischemic stimuli should be applied for optimal protection and what their duration and timing should be. The variation in IPC protocols used in clinical trials is very limited: three cycles of 5 minutes of ischemia are applied to the upper arm in the vast majority of cases. Furthermore, there is little evidence on whether local IPC and remote IPC are equally effective, and the extent to which animal/patient characteristics such as gender and co-morbidities interfere with these cardioprotective strategies has not been systematically analyzed. Lastly, the quality of the available preclinical evidence has not been assessed. As a consequence, the external validity of the animal models is unclear and the ischemic conditioning stimulus used in clinical trials could be suboptimal or incorrectly applied, or unsuitable for the patient population.

In parallel to previous work in *e*.*g*. ischemic heart disease, renal IPC and stroke[13–15], we propose that systematic review and meta-analysis of animal studies can provide helpful insights to the impact of study design and quality, within the limitations associated with combining data from different studies. Therefore, we set out to 1) summarize the evidence supporting the efficacy of IPC in animal models of MI using meta-analysis, 2) ascertain the conditions of maximum efficacy to inform the design of future clinical trials and 3) assess the internal and external validity of the animal studies and their effects on reported efficacy.

## Methods

See Text A in S1 File for a detailed description of the methodology used. This systematic review is based on published results of animal experiments studying the effects of local or remote IPC on MI, which we identified using a systematic, computerized search in MEDLINE (via PubMed) and EMBASE. The inclusion criteria and method of analysis were pre- specified and documented in an *a priori* protocol (see http://www.dcn.ed.ac.uk/camarades/).

### 2.1 Literature search strategy, inclusion and exclusion criteria

We performed a systematic search in PubMed and EMBASE on January 2^nd^ 2014, using the search components “animal”[16,17], “heart”, “ischemia reperfusion injury” and “preconditioning” (see Text B in S1 File for full search strategy).

Two independent reviewers assessed all references and included studies if 1) the study assessed the effect of IPC on myocardial infarction and reported the mean and variance of the infarct size as a percentage of the area at risk (IS/AAR%), as well as the number of animals that these figures were based on; 2) the study was performed in animals *in vivo;* 3) the study was an original full paper which presented unique data.

### 2.3 Study characteristics and data extraction

In each publication, we identified all independent comparisons of the infarct size in IPC-treated animals *versus* controls. We extracted data on the animals used, the myocardial infarction model and the IPC intervention (see Figure A in S1 File). We extracted data on infarct size if raw data or group averages, standard deviation (SD) or standard error (SE) and number of animals per group (n) were reported, or could be recalculated. In case of missing outcome measure data, we attempted to contact authors for additional information.

### 2.5 Assessment of methodological quality

Two independent reviewers assessed each publication using the SYRCLE Risk of Bias tool[18]. In addition, we extracted data on reporting of any measure of randomization and any measure of blinding, in order to distinguish between reporting of measures to report bias and actual risk of bias. We assessed reporting of a sample size calculation an additional study quality indicator.

### 2.6 Data synthesis and statistical analyses

Data were analyzed using STATA/SE, version 11.2 (StataCorp, College Station, TX, USA). All data were extracted in the same unit of analysis (IS/AAR%). For each independent comparison, we calculated an effect size as a raw difference in means (MD; the mean of the experimental group minus the mean of the control group) of IS/AAR% and corresponding 95% confidence interval (CI). To account for anticipated heterogeneity, we pooled effect sizes using random effect meta-analysis, which takes into account the precision of individual studies and the variation between studies and weights each study accordingly. Heterogeneity was quantified using I^2^ and Τ^2^ statistics (see [19] for meta-analysis details).

We performed pre-specified subgroup analyses, using meta-regression, to explore sources of heterogeneity and assess the impact of methodological and study quality indicators on the observed efficacy of IPC. Subgroup analyses were performed when ≥ 2 of its categories contained ≥ 10 comparisons. The percentage of between-study variance explained by variables of interest was assessed using the T^2^ and adjusted R^2^ statistics. We adjusted our significance level using the Holm-Bonferroni method[20] (1-(1-α)^1/m^) to account for multiple comparisons. We assessed for potential publication bias by visual inspection of funnel plots for asymmetry, performing Duval and Tweedie's trim and fill analysis[21] and Egger's regression analysis[22] for small study effects.

### 2.7 Sensitivity analyses

To assess the robustness of our findings, sensitivity analysis was performed for the cut-off points for the subgroups of total IPC ischemia and IPC to IR delay, and exclusion of myocardial infarction models using a coronary-to-carotid artery shunt for cardiac reperfusion. Post-hoc, we tested the robustness of our findings by performing separate analyses for RIPC and LIPC, and by re-running our analysis using the standardized difference in means (SMD).

## Results

### 3.1 Study selection process

**Fig 1:**
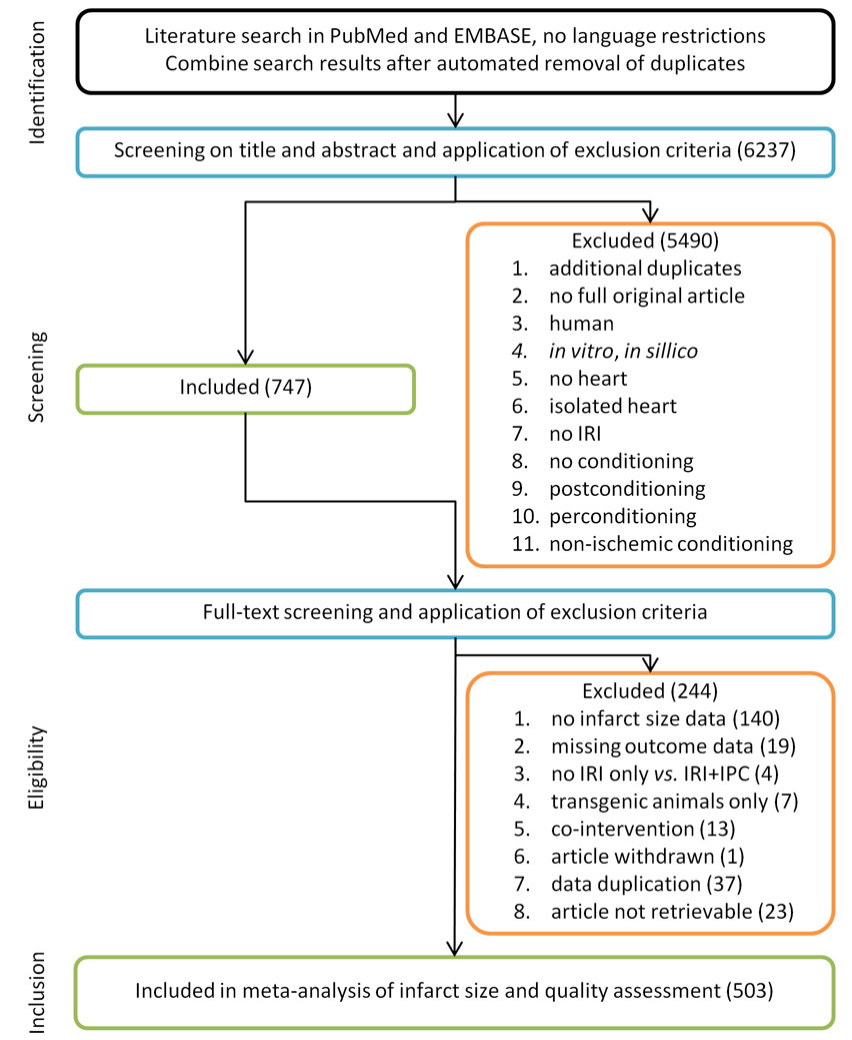
Flow chart of the study selection process. A systematic search in PubMed and EMBASE yielded 6237 unique publications. After application of inclusion and exclusion criteria, data from 503 publications were included in the meta-analysis and quality assessment.

Fig 1 depicts a flow chart of the study selection process. Out of 6237 publications retrieved from PubMed or EMBASE, 747 were included after screening of title and abstract. Out of these 747 publications, 503 fulfilled our inclusion criteria (see reference list Text C in S1 File).

### 3.2 Study characteristics

From the 503 publications, we extracted data of 785 controlled comparisons of IPC in models of myocardial IR (see Figure B in S1 File). In total, our analysis includes data obtained from 5237 control animals and 6251 animals undergoing IPC. Characteristics of all studies and comparisons are listed in Table A in S1 File.

#### 3.2.1 Animal characteristics

See Table 1. Out of 785 comparisons, 44% (344/785) were experiments conducted in rodents (67 in mice, 277 in rat), and 56% (441/785) of the data were from other species, predominantly rabbits (263 comparisons), dogs (92 comparisons) and pigs (72 comparisons). Data were predominantly obtained from male animals (58% of comparisons), or groups of mixed sex (19% of comparisons). Only 5% (36/785) of comparisons represented data obtained from females, whereas 20% (99/503) of publications failed to report the sex of the animals used. Ninety-four percent of the animals studied were healthy, young adults (739/785 comparisons). Animals suffering from comorbidities were used in only 6% (46/785) of comparisons. Out of all conditions, diabetes was studied most frequently (2% of comparisons). Administration of opioids pre-IPC, which may induce pharmacological preconditioning, was reported for 5% (41/785) of comparisons. However, most publications did not specify whether or not any opioids were used (93% of comparisons).

**Table 1:** Meta-analysis animal model and IPC characteristics.

	# pub	# comp	(%)	MD	[95%CI]
**all** (T^2^ 164.4, I^2^ 94.7%)	503	785	(100)	24.6	[23.5, 25.6]
**species**					
P<0.0001, adj. R^2^ 5.94%					
dog	66	92	(12)	17.7	[14.9, 20.4]
ferret	1	5	(0.6)	9.4	[-3.2, 21.9]
macaque	1	1	(0.1)	41.8	[15.0, 68.6]
mouse	45	67	(9)	25.8	[22.6, 28.9]
pig	48	72	(9)	28.3	[25.0, 31.7]
rabbit	169	263	(34)	23.3	[21.6, 25.0]
rat	171	277	(35)	27.1	[25.5, 28.7]
sheep	5	8	(1)	24.8	[15.5, 34.0]
**species**					
P<0.0001, adj. R^2^ 2.47%					
total rodents	214	344	(44)	26.8	[25.3, 28.2]
total non-rodents	290	441	(56)	22.7	[21.4, 24.1]
**sex**					
P<0.0001, adj. R^2^ 3.44%					
not reported	99	146	(19)	25.0	[22.7, 27.3]
female	22	36	(5)	28.2	[23.5, 33.0]
male	281	457	(58)	25.7	[24.5, 27.0]
mixed	101	146	(19)	19.7	[17.5, 22.0]
**conditions**					
P<0.0009, adj. R^2^ 3.38%					
none / healthy	490	739	(94)	25.0	[24.0, 26.0]
brain death	3	3	(0.4)	10.0	[-5.0, 24.8]
cardiac hypertrophy	2	4	(0.5)	26.8	[14.1, 39.5]
coronary artery stenosis	1	1	(0.1)	30.7	[3.9, 57.5]
diabetes	11	13	(2)	10.2	[2.9, 17.6]
hypercholesterolemia	6	7	(0.9)	21.1	[10.9, 31.2]
hyperglycaemia	6	8	(1)	17.8	[8.5, 27.0]
hypertension	2	2	(0.3)	48.5	[29.2, 67.8]
obesity	1	1	(0.1)	10.1	[-16.8, 37.0]
ovariectomy	1	1	(0.1)	24.5	[-4.4, 53.4]
middle-age	1	1	(0.1)	28.0	[1.4, 54.6]
senescence	5	5	(0.6)	13.1	[0.7, 25.5]
(total conditions	36	46	(6)	17.7	[13.8, 21.7])
**pre-IPC opioid use**					
P = 0.47, adj. R^2^ 0.04%					
yes	33	41	(5)	22.2	[17.9, 26.5]
none	8	12	(2)	24.7	[23.7, 25.8]
not reported	462	732	(93)	22.5	[14.6, 30.4]
**IPC site**					
P = 0.18, adj. R^2^ 0.31%					
local	477	728	(93)	24.8	[23.8, 25.8]
total remote	45	55	(7)	21.5	[17.8, 25.2]
local + remote	2	2	(0.2)	34.8	[9.9, 59.7]
**IPC ischemic cycle duration**					
P<0.04, adj. R^2^ 1.48%					
1 minute	4	4	(1)	20.7	[6.6, 34.8]
2 minutes	15	24	(3)	17.4	[11.7, 23.1]
3 minutes	41	57	(7)	21.6	[18.0, 25.2]
4 minutes	35	51	(7)	26.0	[22.2, 29.8]
5 minutes	361	522	(67)	25.4	[24.2, 26.6]
7 minutes	2	2	(0.3)	22.9	[3.8, 42.0]
8 minutes	1	1	(0.1)	28.0	[-3.1, 59.1]
10 minutes	59	86	(11)	25.3	[22.2, 28.4]
15 minutes	16	33	(4)	19.1	[14.1, 24.0]
20 minutes	2	2	(0.3)	6.4	[-12.7, 25.6]
25 minutes	1	1	(0.1)	29.0	[1.7, 56.3]
30 minutes	2	2	(0.3)	15.1	[4.3, 34.4]
**IPC # ischemic cycles**					
P<0.0001, adj. R^2^ 3.8%					
1 cycle	222	308	(39)	22.5	[21.0, 24.1]
2 cycles	88	113	(14)	28.9	[26.4, 31.5]
3 cycles	126	158	(20)	27.7	[25.6, 29.9]
4 cycles	91	124	(16)	22.1	[19.7, 24.5]
5 cycles	5	6	(0.8)	30.8	[19.1, 42.6]
6 cycles	35	49	(6)	24.8	[20.9, 28.7]
7 cycles	2	2	(0.3)	28.3	[-1.0, 57.7]
8 cycles	3	3	(0.4)	1.8	[-19.9, 23.4]
9 cycles	7	7	(0.9)	17.5	[7.3, 27.7]
10 cycles	1	2	(0.3)	24.8	[3.5, 46.1]
12 cycles	5	12	(2)	21.9	[14.2, 29.6]
25 cycles	1	1	(0.1)	6.3	[-29.6, 42.2]
**IPC total ischemia**					
P<0.0007, adj. R^2^ 2.34%					
0–2 minutes	9	9	(1)	11.9	[2.3, 21.4]
3–6 minutes	181	230	(29)	23.4	[21.6, 25.2]
7–17 minutes	225	314	(40)	26.4	[24.9, 28.0]
18–44 minutes	153	215	(27)	24.2	[22.3, 26.1]
45–110 minutes	13	17	(2)	16.2	[9.2, 23.1]
**IPC to IRI delay**					
P<0.0001, adj. R^2^ 9.72%					
0–6 minutes	229	306	(39)	27.3	[25.8, 28.8]
7–44 minutes	229	310	(40)	25.8	[24.2, 27.3]
45–278 minutes	28	45	(6)	8.6	[4.2, 13.0]
279–1754 minutes	83	109	(14)	19.6	[17.0, 22.1]
1755–11066 minutes	7	13	(2)	16.1	[8.2, 23.9]
not reported	2	2	(0.3)	19.0	[-1.6, 39.6]

Total # comparisons = 10, corrected P<0.005; IPC = ischemic preconditioning, pub = publications, comp = comparisons, MD = weighted difference in means, adj. = adjusted, IAO = infrarenal aorta occlusion, LCX = left circumflex coronary artery, MAO = mesenteric artery occlusion, RAO = renal artery occlusion, IRI = ischemia-reperfusion injury.

#### 3.2.2 IPC characteristics

See Table 1. Although in most comparisons (93%) the IPC stimulus was applied locally by clamping the coronary artery, in 7% (55/785) of the cases the preconditioning stimulus was applied to a remote organ. The duration of one ischemic cycle of the IPC protocol ranged from 1 to 30 minutes, with the majority of studies using five minute duration for one ischemic cycle (67% of comparisons). The number of ischemic cycles applied during IPC varied from 1 to 25, although most studies used either one (39% of comparisons) or three cycles (20%). The total duration of all ischemic cycles in the IPC protocol ranged from 1 to 90 minutes, with 40% (314/785) of comparisons applying 7 to 16 minutes of ischemia in total. The delay between the application of the IPC protocol and the induction of myocardial IR was less than 40 minutes in most studies, corresponding to the so-called first or early window of protection (79% of comparisons). Delays of 40 to 240 minutes and of more than 24 hours between IPC and IRI were studied least frequently (respectively 6% and 2% of comparisons).

#### 3.2.3 Study quality and risk of bias characteristics

**Fig 2:**
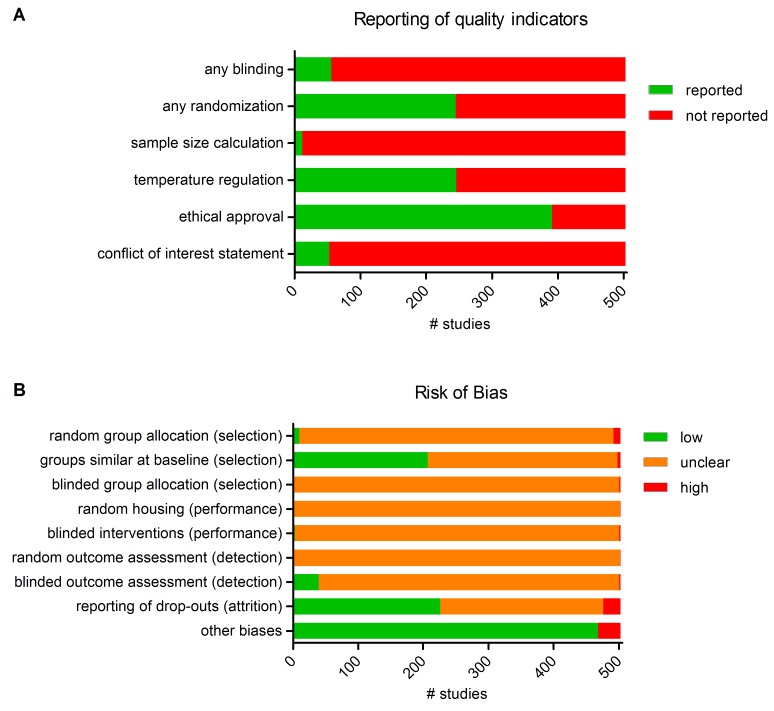
Reporting of study quality indicators and risk of bias in 503 included studies. **A**, Reporting of three key study quality indicators was found to be poor in many cases. **B**, Using SYRCLE's risk of bias tool, the risks of selection, performance, detection, attrition and other biases were assessed. Lack of (adequate) reporting of measures to reduce bias resulted in a high percentage of unclear risk of bias for most items.

For the study quality and risk of bias assessment of individual studies, see Table B in S1 File. The overall reporting of study quality indicators for the 503 included publications is presented in Fig 2. For 49% (245/503) of publications, it was reported that the experiment was randomized in some way. Blinding of the experiment at any level was reported in only 11% (56/503) of the cases. In only 2% (12/503) of the publications, a sample size calculation supporting the chosen group sizes was reported. Nearly half of the publications (49%) stated that the temperature of the animals was controlled within a physiological range during surgery. A statement of ethical approval for the animal experiment was reported in the majority (77%) of publications. A conflict of interest statement was present in 53/503 (10%) out of publications, four of which reported a possible conflict of interest.

When assessing individual items in the risk of bias tool, inadequate reporting led to an unclear risk of bias in many cases (Table C in S1 File and Fig 2). Of three measures to reduce selection bias, random and blind group allocation were most poorly reported: details of the method used were unclear in 482/503 and 500/503 publications, for randomization and blinding respectively. In 3% (13/503) of publications, the risk of selection bias was assessed as high, caused by explicit non-randomized or non-blinded allocation. Reporting of baseline characteristics of control and experimental groups indicated a low risk of selection bias in 41% (207/503) and a high risk of bias in 5 publications. In 58% of the cases, data for one or more baseline characteristics were missing, leading to an unclear risk of bias.

Measures to reduce performance bias were reported in only 3 publications, in the form of blinding of staff during the intervention. We identified 3 studies with a high risk of performance bias, due to explicit non-randomized housing or non-blinded staff. The risk of performance bias was unclear in >99% of publications.

Concerning the risk of detection bias, random outcome assessment was reported only once, and blinding of the outcome assessment was reported in 8% (40/503) of publications, leading to a low risk of bias assessment for these studies. Again, most publications failed to provide details on measures to reduce detection bias (unclear risk of bias in >90% of studies).

Attrition bias was evaluated as being low in 45% (226/503) of studies, in which the number of animals excluded from the study (drop-outs), as well as the reason for exclusion, were specified and clear. The description of drop-outs was insufficient to assess attrition bias in 50% (250/503) of the publications, leading to an unclear risk of bias. The risk of attrition bias was assessed as being high in 27 publications.

When assessing other potential sources of bias, we found that in 46 studies the initial number of animals allocated to each experimental group differed substantially between groups, but that all groups were of equal size after exclusion of drop-outs. Some studies explicitly stated that drop-outs were replaced with new animals until a certain number of animals per group was reached. If this was the case, and the number of replacements differs substantially between groups, we regarded this as a potential source of bias. In two publications, the experimental procedure for the IPC *versus* the control group was not identical, which was also regarded as a potential source of bias.

### 3.3 Meta-analysis of IPC efficacy

Overall, IPC reduced infarct size in the area at risk by a mean difference of 24.6% [23.5–25.6], when compared to untreated controls (P<0.001; n = 785 comparisons, see also Table 1). We observed substantial heterogeneity (Τ^2^ = 164.4 and I^2^ = 94.7%; P<0.001).

#### 3.3.1. Impact of animal and IPC characteristics on IPC efficacy

**Fig 3:**
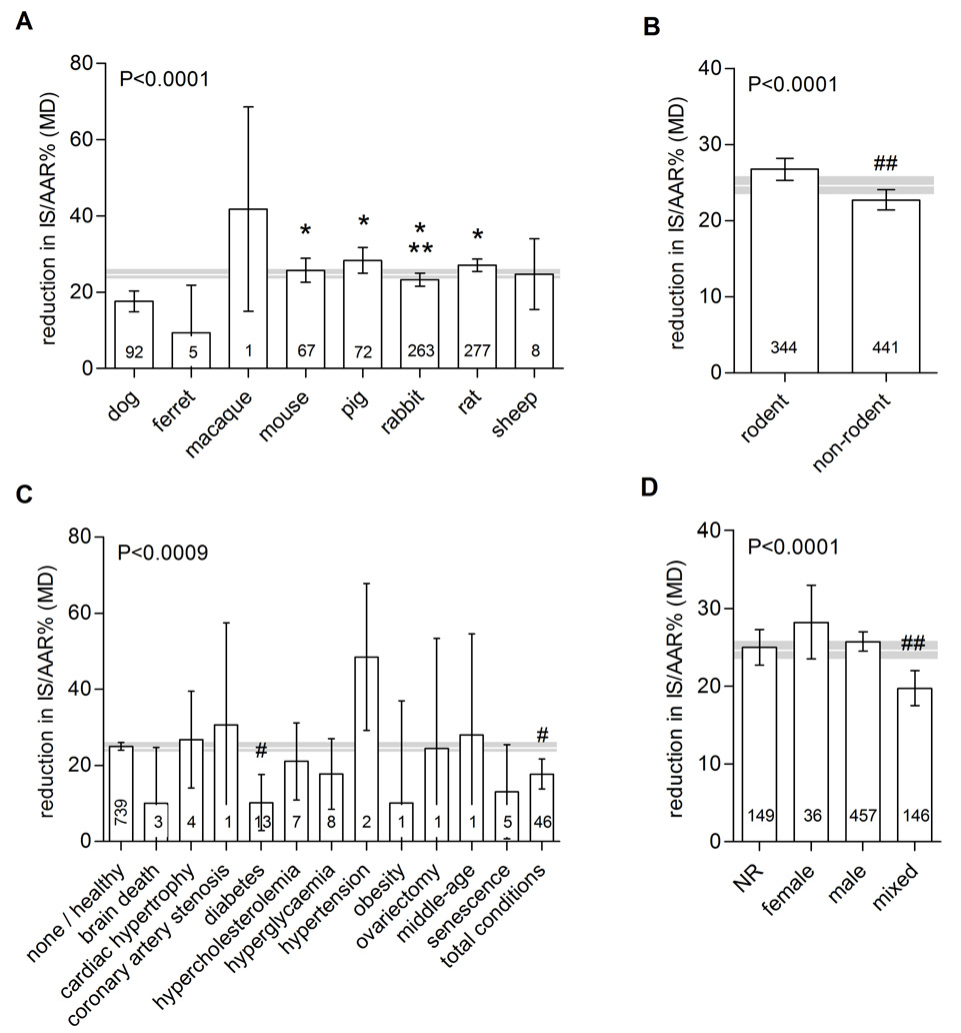
Impact of animal characteristics on IPC efficacy. Data are presented as raw difference in means (MD) in infarct size as a percentage of the area at risk (IS/AAR%). Horizontal white line and grey bar represent the pooled effect estimate and its 95% confidence interval. The number of comparisons contributing data is indicated in each bar. *differs from dog; **differs from rat; ^#^differs from total conditions; ^##^differs from all other groups.

The species used accounted for a substantial proportion of between-study heterogeneity (P<0.0001; Τ^2^ = 154.7; adjusted R^2^ = 5.94%). IPC reduced infarct size in all animal species, except for ferrets, and was more effective in rodents than in non-rodents (Table 1; Fig 3). Although effective in both sexes, IPC efficacy differed depending on the sex of the animals (male *versus* female *versus* mixed *versus* not reported, P<0.0001). Differences related to sex accounted for 3.4% of the between-study variance (Τ^2^ = 158.8). The use of animals with comorbidities also had a significant influence on IPC efficacy (P<0.0001; Τ^2^ = 158.9; adjusted R^2^ = 3.4%). Comparing healthy animals to animals modelling any comorbidity or condition, IPC efficacy in the latter group was reduced (ΔMD 7.3[3.1, 11.5]). The protective effect of IPC was significantly reduced in diabetic animals (ΔMD 14.8[7.2, 22.4] *versus* healthy animals). IPC efficacy also appeared to be affected in brain dead and senescent animals, but the number of experiments in these subgroups was small. The use of opioids pre-IPC had no apparent influence on IPC efficacy (yes *versus* none *versus* not reported, P = 0.47).

**Fig 4:**
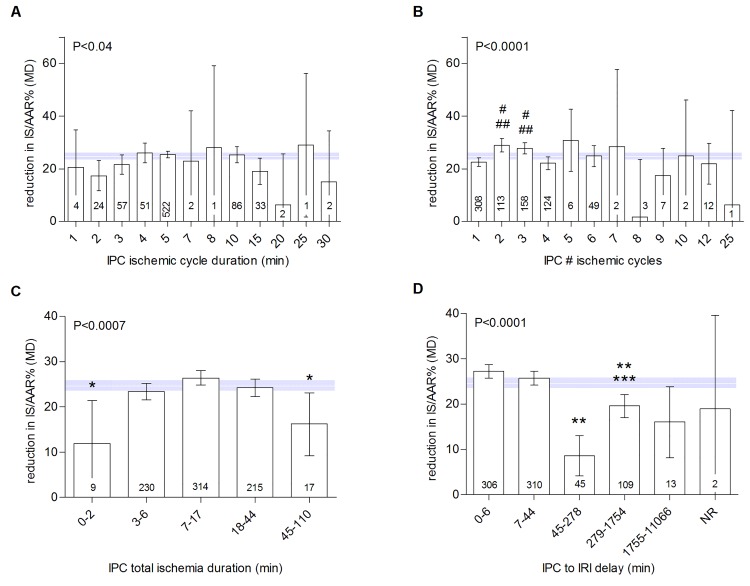
Impact of IPC protocol characteristics on IPC efficacy. Data are presented as raw difference in means (MD) in infarct size as a percentage of the area at risk (IS/AAR%). Horizontal white line and grey bar represent the pooled effect estimate and its 95% confidence interval. The number of comparisons contributing data is indicated in each bar. *differs from 7–17 min; **differs from 0–6 and 7–44 min; ***differs from 45–278 min; ^#^differs from 1 cycle; ^##^differs from 4 cycles.

For factors related to the IPC stimulus (Table 1; Fig 4), meta-analysis showed that both local and remote IPC reduced infarct size and that efficacy was equal between these groups (local *versus* remote *versus* both, P = 0.18). IPC efficacy was also not influenced by the duration of each ischemic cycle of the IPC protocol (P<0.04). The number of ischemic cycles applied and the total duration of ischemia during IPC did influence IPC efficacy (P<0.0001 for number of cycles and P<0.0007 for total durations) and accounted for 3.8% (Τ^2^ = 158.2) and 2.34% (Τ^2^ = 160.6) of between-study variance respectively. Protocols using 2 or 3 cycles of ischemia induced greater protection than those using 1 cycle. No reduction of infarct size after IPC was observed after 7, 8 and 24 cycles of IPC ischemia, but data in these subgroups were limited. Furthermore, IPC efficacy was influenced by the delay between IPC and myocardial IRI (P<0.001), which accounted for a substantial proportion of between study-variance (Τ^2^ = 148.4; adjusted R^2^ = 9.72%). IPC efficacy appears to be highest for short delays between IPC and MI (up to 6 minutes) and between 7 and 44 minutes. A second, although less potent, peak in efficacy is observed for delays of >4h - 24h. Efficacy was diminished in studies using delays between 45 and 278 minutes and those longer than 24 hours.

#### 3.3.2. Impact of study quality and risk of bias on effect estimates

We performed subgroup analysis of the reporting of randomization and blinding at any level of the experiment, as well as on each individual item of the risk of bias tool, to assess their influence on effect estimates (Table C in S1 File). Our analysis showed no difference in IPC efficacy between studies reporting any form of randomization or blinding *versus* studies that did not report any details on these items. This result was comparable in studies using rodents *versus* non-rodent species.

For the individual items of the risk of bias tool, no differences in IPC efficacy were observed between studies with a high, low and unclear risk of bias. The amount of between-study variance explained by any of the items was highest for attrition bias (Τ^2^ = 162.3; adjusted R^2^ = 1.29%). The influence of blinded allocation, random housing, blinding of staff and random outcome assessment could not be analyzed due to insufficient data.

### 3.4 Publication bias

**Fig 5:**
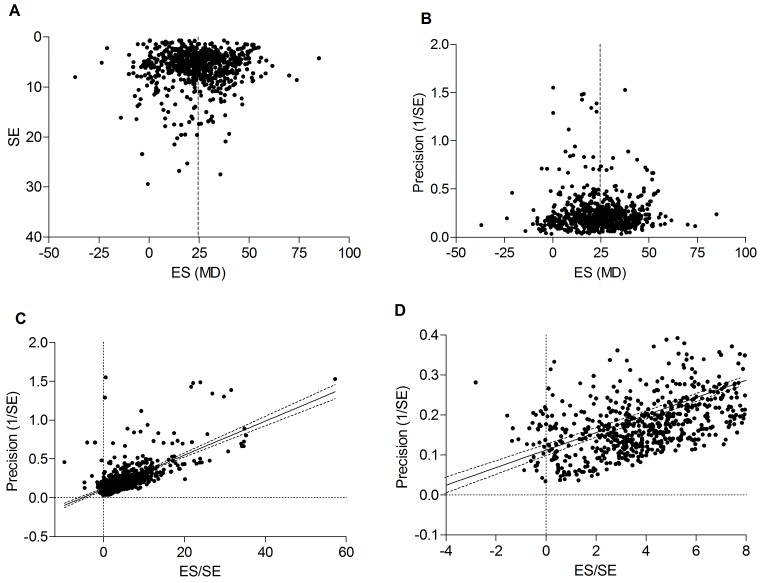
Assessment of publication bias. Visual inspection of funnel (**A**) and precision (**B**) plots suggested an underrepresentation of studies with moderate precision and small effect sizes, but this was not confirmed by trim and fill analysis. Dotted line represents the overall effect size of 24.6. **C-D:** Egger's regression test revealed that small study effects are likely present, since the regression line does not intercept at the origin. ES = effect size; SE = standard error; MD = raw difference in means.

Visual inspection of the funnel and precision plots (Fig 5A and 5B) suggest asymmetry consistent with an underrepresentation of studies with moderate precision and small effect sizes. However, Duval and Tweedie's trim and fill analysis did not identify any missing studies, indicating a possible absence of bias. In Egger's regression test (Fig 5C and 5D) the null-hypothesis of no small study effects was rejected at P<0.001 (estimated bias coefficient = 0.82 ± 0.22 SE). Overall, we estimate that the risk of publication bias in this dataset is small.

### 3.5 Sensitivity analysis

Changing the increments for categorization of total IPC ischemia from log_10_(0.4) to log_10_(0.3) or log_10_(0.5) minutes had no significant effect on the outcome of this subgroup analysis (see Figure C, panel A-C in S1 File). Similarly, the outcome of the subgroup analysis of IPC to IR delay was virtually unchanged when changing the increment between categories from log_10_(0.8) minutes to log_10_(0.7) or log_10_(0.9) minutes (see Figure C, panelD-F in S1 File). The effect of both variables on IPC efficacy remained significant and the percentage of heterogeneity explained was similar.

Omitting data from the 13 studies using a coronary-to-carotid artery shunt from the analysis had no notable effect on meta-analysis outcomes for the summary effect (MD 24.4[23.4, 25.5]) or any of the subgroup analyses.

When re-running our analysis using the SMD, all results were similar to those found using the MD. We found a highly significant (p<0.000) summary SMD of 2.6 with a CI of [2.5, 2.7]. The magnitude and direction of effect, as well as the level of significance and amount of heterogeneity explained by the various subgroup variables did not change.

The site of IPC (local versus remote) did not explain a significant amount of heterogeneity. However, following suggestions from experts in the field, we performed post-hoc analysis on the separate datasets for RIPC and LIPC. For LIPC, subgroup analysis outcomes were unchanged, except for the duration of one ischemic cycle, which now also explained a significant proportion of heterogeneity. For RIPC, many subgroup analyses could not be performed due to insufficient data. Species (rodent vs. non-rodent) accounted for a substantial part of heterogeneity, but the number, timing and duration of RIPC cycles did not (data not shown).

## Discussion

This systematic review and meta-analysis of animal studies provides, for the first time, insight in the vast amount of data available on IPC of the heart. As expected, we find IPC to be highly effective in animal models, reducing myocardial infarction in various species, under variable circumstances, using different protocols. We also provide essential clues as to why these impressive results have been difficult to reproduce in patients.

### 4.1 The optimal IPC protocol

Overall, we found that IPC reduced IS/AAR by 25%. There was no difference in efficacy between studies using LIPC *versus* those using RIPC. This is an important observation as it may facilitate translation to the clinical setting, where RIPC is favoured because it is non-invasive, safe and low-cost. Indeed, nearly all clinical trials investigate RIPC, rather than LIPC. This is in sharp contrast to animal studies, of which only 45 studies (7% of data) investigated RIPC.

We found no effect of the duration of each ischemic cycle of the IPC protocol on treatment efficacy. However, the number of cycles did influence treatment efficacy, as did the total duration of ischemia during the protocol. Our findings suggest that protocols using 2–3 cycles of 4–5 minutes of ischemia provide optimal protection. Longer ischemic duration or more ischemic cycles do not offer additional benefit, and may even result in loss of the protective effect.

Importantly, IPC stimuli applied <45 minutes before MI (timed for the so-called 'early window of protection') were found to be more potent than stimuli applied approximately 4–29 hours before MI (second/late window of protection). Efficacy is markedly diminished in the intermediate period (45–278 minutes before MI). This indicates that for optimal protection, the delay between IPC and MI should be short. Of note, these findings contradict those for renal IPC[15], where the second window of protection is more potent. However, for renal IPC the average delay in the 'late' subgroup averaged 17±23 days, whereas for cardiac IPC such long delays are scarce. Furthermore, the optimal delay could be organ specific and dependent on the underlying mechanism of protection.

### 4.2 External validity

Our analysis shows that cardiac IPC is highly reproducible across various species in terms of the direction and magnitude of effect, which is considered to be advantageous for its translation to humans. However, we also found striking differences between the animal models used and the patients for whom treatment is intended. Most striking is the limited number of studies performed in comorbid animals; only 6% of data was obtained in animals exhibiting comorbidities, including comorbidities commonly present in patients with cardiovascular disease, such as diabetes and obesity. None of the animals suffered from more than one comorbidity, although patients typically do. Animals also do not match the patient population in terms of age, with a very small minority of studies performed in middle-aged or senescent animals and no studies performed in juvenile animals. The latter finding is of interest in view of a recent meta-analysis of clinical trials in paediatric patients undergoing congenital cardiac surgery[23], which showed no protective effect of RIPC. Our observation that IPC efficacy is reduced in senescent animals is in line with the finding that protective effects of IPC on endothelial function are lost in elderly patients[24].

Only 5% of the animals were female, indicating a sex bias in these data. Our subgroup analysis for sex differences was hampered as many studies did not report the sex of the animals used, and studies using groups of mixed sex did not present the data per sex. Our observation that IPC efficacy is lower in experimental groups of mixed sexes warrants further research on the effect of sex on IPC efficacy.

In terms of clinical application, non-invasive, safe and low-cost RIPC is favoured over invasive LIPC and clinical trials have therefore focused almost exclusively on RIPC. Conversely, the amount of animal study data available on LIPC far exceeds that of RIPC. This is partly due to the fact that investigators now use LIPC as a control intervention when assessing the efficacy of novel preconditioning strategies. However, basic studies on LIPC are still frequently performed. One might question the external validity of such experiments, since follow-up with a clinical trial on LIPC is considered unfeasible. Simultaneously, the amount of data available on RIPC efficacy seems limited in relation to the number of clinical trials on RIPC already performed or in progress. The question arises whether the amount and quality of the preclinical evidence on RIPC has been sufficient to optimally inform these clinical trials.

### 4.3 Internal validity

Our assessment of study quality and risk of bias illustrates that the field of cardiac IPC is no exception to the insufficient reporting of animal studies. This is a matter of concern, since there is growing evidence that lack of measures to reduce bias can severely influence primary study results (*e*.*g*. [10]). We found no difference in summary effect between studies reporting any form of randomization *versus* studies not reporting this. However, the interpretation these results is hampered by insufficient reporting: the subgroup of studies not reporting randomization likely consists of non-randomized studies, as well as studies that were randomized, but failed to report this. Concurrently, studies reporting randomization do not provide details necessary to assess whether or not randomization was actually achieved and at which phase of the experiment. The "randomized" subgroup therefore likely consists of truly randomized as well as non-randomized studies. The same holds true for blinding. The lack of reporting of randomization and blinding are especially worrying in view of the poor reporting of temperature regulation and equal baseline characteristics.

Furthermore, our analysis shows that assessment of the risk of bias is severely hampered by poor reporting. The small amount of detail provided on various aspects of experimental design resulted in most of the risk of bias items to be assessed as unclear. This is why the result of our meta-analysis, which did not indicate any effects of the level of bias on summary effects, is likely to be underpowered and should be interpreted with care. The importance of avoiding bias is emphasized once more by a meta-analysis of clinical trials of IPC reporting an overall protective effect of RIPC, but no effect in a subgroup of studies with blinded outcome assessment[7].

The justification of group sizes using a sample size calculation is very scarce, even though this key element of experimental design is obligatory for approval by most animal ethics committees. The sample size calculation should include the expected mean and variation of the primary outcome measure, as well as the effect size the authors aim to detect. Specifying this prevents changing the primary outcome based on the study results, thereby reducing the risk of bias due to selective outcome reporting[25]. Correct sample size calculation also prevents unethical use of animals due to overpowering (using more animals than necessary) or underpowering (using too few animals, especially relevant in case no effect of the intervention is found). Furthermore, knowing the planned sample size alerts the reader to potential problems, such as: Have the authors adequately adjusted their planned sample size for expected drop-outs? Were drop-outs replaced until a certain number of animals per group was reached? Was the experiment non-blinded and stopped as soon as a significant effect was reached?

### 4.4 Clinical relevance and recommendations

Our analysis shows that an IPC stimulus of 2–3 cycles of 4–5 minutes of ischemia, applied directly before index ischemia provides optimal protection in animal models. When comparing these findings to the design of clinical trials, we found that the number IPC cycles used is in the same range: in most clinical trials 3–5 cycles of 5 minutes of ischemia and reperfusion are applied[5–8]. Our analysis indicates that perhaps these protocols could be shortened to 2–3 cycles for optimal efficiency. Furthermore, our finding that RIPC and LIPC are equally effective supports the current focus of clinical trials on RIPC. Importantly, in many clinical trials the IPC stimulus is applied >45 minutes prior to the ischemic event, a time-point which, according to our analysis, is associated with reduced protection. This may partly explain the reduced efficacy of IPC found in clinical trials. Based on our analysis it is important for future clinical trials to apply the IPC stimulus <45 minutes prior to index ischemia.

In addition to possible sub-optimal timing of the IPC protocol, we postulate that the poor resemblance of the animals used to the patient population poses a major threat to successful translation of IPC to patients. Future studies using animal models should strive to improve the external validity in terms of comorbidity, sex and age, and should demonstrate efficacy of RIPC. Furthermore, our work clearly illustrates the urgent need to improve the reporting and quality of animal studies. Measures to reduce bias and increase external validity are essential for animal studies to yield relevant, robust data which benefits patients. As we show here, adequate reporting (*e*.*g*. using [26] [27]) is essential to allow researchers, clinicians and policy makers to assess the quality of the evidence presented in animal studies. Ultimately, differences in results between animal studies and clinical trials may be due to fundamental biological/physiological differences between humans and other species. However, this is very difficult to assess where poor reporting prevents an accurate assessment of the internal and external validity of many animal studies. Nevertheless, the development and use of experimental models for IPC in healthy human volunteers and/or patients may offer many benefits for the study of IPC efficacy, its mechanism of action and its translation to clinical practice.

### 4.5 Limitations of this review

We observed substantial heterogeneity in our meta-analysis. However, in meta-analysis of pre-clinical studies, the included studies may not aim to measure a single treatment effect. Instead, animal studies generally aim to assess differences in treatment effects under different circumstances. They are therefore by nature more heterogeneous than clinical trials, which often report lower heterogeneity. We used a random (rather than fixed) effects meta-analysis to account for this expected heterogeneity, and the effect sizes presented should be considered average effects, rather than precise estimates. Importantly, pooling of the studies based on study characteristics and quality allowed us to explore sources of their heterogeneity, and the insights provided in these sources should be considered the most important findings of our meta-analysis. We did not aim to provide precise effect estimates and the results of this review should be regarded as hypothesis-generating.

The statistical methods used to assess publication bias have high power due to the large number of included comparisons. Of note, Egger's regression test is vulnerable to type I errors when heterogeneity is high, or when sample sizes are very similar between studies, which may have influenced our analysis[28]. However, based on the outcome of all three methods, we consider the risk of publication bias to be small in our dataset.

## Supporting Information

S1 PRISMA Checklist PRIMSA Checklist. (PDF)

S1 File Text A, extended methods. Text B, search strategy. Figure A, schematic representation of ischemia-reperfusion injury and ischemic preconditioning. Figure B, individual effect estimates of all 785 included comparisons. Figure C, sensitivity analysis total duration IPC ischemia and IPC to IRI delay. Table A, study and comparison characteristics. Table B, study quality and risk of bias for individual studies. Table C, subgroup analysis risk of bias items. Text C, included studies reference list.
(PDF)

## Abbreviations

AAR: area at risk
CI: confidence interval
IPC: ischemic preconditioning
IRI: ischemia-reperfusion injury
IS: infarct size
LIPC: local ischemic preconditioning
MD: raw difference in means
MI: myocardial infarction
PRISMA: preferred reporting items for systematic reviews and meta-analyses
RIPC: remote ischemic preconditioning
SMD: standardized mean difference
