# Supplementary material for: Determinants of the Efficacy of Cardiac Ischemic Preconditioning: A Systematic Review and Meta-Analysis of Animal Studies
Source: PLoS One. 2015 Nov 18;10(11):e0142021. doi: 10.1371/journal.pone.0142021 (PMC4651366; doi:10.1371/journal.pone.0142021)
Supplement: S1 File — Figure A, schematic representation of ischemia-reperfusion injury and ischemic preconditioning. Figure B, individual effect estimates of all 785 included comparisons. Figure C, sensitivity analysis total duration IPC ischemia and IPC to IRI delay. Table A, study and comparison characteristics. Table B, study quality and risk of bias for individual studies. Table C, subgroup analysis risk of bias items. Text C, included studies reference list. (PDF) [file pone.0142021.s002.pdf]

S1 File - Wever et al. 2015 - Determinants of the efficacy of cardiac ischemic preconditioning: a systematic review and meta-analysis of animal studies

Text A - Detailed Methods

This systematic review is based on published results of animal experiments studying the effects of local or remote IPC on MI, which we identified using a systematic, computerized search in MEDLINE (via PubMed) and EMBASE. The inclusion criteria and method of analysis were pre-specified and documented in an *a priori* protocol (see http://www.dcn.ed.ac.uk/camarades//). This review is reported according to the PRISMA guidelines.

1.1 Amendments to the systematic review protocol

We extracted data on three additional study quality indicators, to obtain a comprehensive overview of study quality, and to allow for better comparison with systematic reviews in other fields of research. The additional items were: reporting of a conflict of interest statement (Y/N), reporting of ethical approval of the experiment (Y/N) and reporting of regulation of body temperature within a physiological range during surgery (Y/N). We did not perform meta-analysis on these items.

We omitted the planned subgroup analysis of total risk of bias score due to the poor reporting of many of the risk of bias items, since we expect this would severely hamper the analysis and would likely produce unreliable results.

1.2 Literature search strategy, inclusion and exclusion criteria

The full search strategies for PubMed and EMBASE are included in the supplemental material, and involved the search components “animal”[1, 2], “heart”, “ischemia reperfusion injury” and “preconditioning”. We searched the databases for published articles up to January 2nd 2014. No language or date restrictions were applied. If necessary, papers in languages other than English were translated by scientists (native speakers for that particular language) within the Radboud university medical center or the University of Edinburgh.

We included studies in the systematic review if they fulfilled all of the following criteria: 1) the study assessed the effect of IPC on myocardial infarction and reported the mean and variance of the infarct size as a percentage of the area at risk (IS/AAR%), as well as the number of animals that these figures were based on; 2) the study was performed in animals *in vivo;* 3) the study was an original full paper which presented unique data. We excluded studies if 1) experiments were performed only in genetically modified animals; 2) animals underwent any co-intervention other than sham IPC or administration of vehicle.

We used Early Review Organizing Software (EROS; Institute of Clinical Effectiveness and Health Policy, Buenos Aires, Argentina) to randomly allocate each reference to two independent reviewers, who screened it for inclusion on the basis of title and abstract. In case of doubt, the whole publication was evaluated. Two independent reviewers subsequently assessed full-text copies of all publications eligible for inclusion and included them if they met our prespecified inclusion criteria. Disagreements were solved by discussion.

1.3 Study characteristics and data extraction

In each publication, we identified all independent comparisons of the infarct size in IPC-treated animals *versus* controls. We extracted data on study design, including the animals used (species, strain, sex, age, weight, and the presence of comorbidities or other conditions), the myocardial infarction model (pre-operative opioid use, anesthetic agent used during surgery, class of anesthetic agent used, site of coronary occlusion, duration of index ischemia and timing of the outcome measure) and the IPC intervention (site of IPC, remote organ, duration of ischemic stimulus, duration of intermittent reperfusion periods, the number of cycles, and interval between IPC and myocardial index ischemia; see supplemental figure SF1). We also recorded bibliographic details such as author, journal, language and year of publication.

We extracted data on infarct size if raw data or group averages, standard deviation (SD) or standard error (SE) and number of animals per group (n) were reported, or could be recalculated. If two or more identical comparisons existed (*e.g.* two separate experiments, with the same control and experimental groups), they were analyzed separately. In case of missing outcome measure data, we attempted to contact authors for additional information. If the data could not be obtained, we used a conservative estimate if possible. In case the group size was reported as a range (*e.g.* 6-9) in combination with an SD, we used the lowest number of animals in our meta-analysis. In case the SE was reported, we used the highest number of animals to recalculate the most conservative estimate of the SD. If no conservative estimate could be made we excluded the comparison from the analysis. If data were presented only graphically, we measured them using digital image analysis software (ImageJ; http://rsbweb.nih.gov/ij/). If multiple experimental groups were compared to the same control group, we corrected the group size of the control group for the number of comparisons made (n/number of comparisons).

1.4 Data handling exceptions

For two comparisons (both from[3]) for which the number of IPC cycles was reported to be 12 or more, we used 12 as the number of cycles in our analysis.

For five comparisons (one each from [4-8]), the duration of the ischemic period of the IPC stimulus differed between cycles. We therefore used the average duration of an ischemic period (total ischemic time/# cycles) in our analysis. The same approach was used for four comparisons in which the duration of the intermittent reperfusion periods differed between cycles (one each from [7, 9-11])

For studies investigating "repeated IPC", *i.e.* applying an interval of one or several days between IPC stimuli, we used the duration of the intermittent reperfusion periods within one IPC stimulus as the intermittent reperfusion duration in our analysis (*e.g.* comparisons from [3, 10, 12-20]). *N.B.* in such cases, the number of IPC cycles used is the total of all IPC cycles in the entire protocol.

One study[8] states that the variance of their data are reported as SD, but are in the SE range. In our meta-analysis, entering these data as SD resulted in an enormous deviation of the study data in terms of variance and weight. We attempted to clarify this issue by contacting the authors, but received no response. We therefore treated the data conservatively and assumed that they were in fact reported as SE.

1.5 Assessment of methodological quality

We used the SYRCLE Risk of Bias tool[21] to assess the risk of bias in the included studies. Two independent reviewers assessed each publication and disagreements were solved by discussion. Concerning the number of excluded animals, we assumed that there had been no exclusion if the number of animals per group mentioned in the materials and methods section was identical to the number stated in the results section or figure legends. In addition, we extracted data on reporting of any measure of randomization and any measure of blinding, in order to distinguish between reporting of measures to report bias and actual risk of bias. We assessed reporting of a sample size calculation an additional study quality indicator.

1.6 Data synthesis and statistical analyses

Data were analyzed using STATA SE, version 11.2 (StataCorp, College Station, TX, USA). All data were extracted in the same unit of analysis (IS/AAR%). For each independent comparison, we calculated an effect size as a raw difference in means (MD; the mean of the experimental group minus the mean of the control group) of IS/AAR% and corresponding 95% confidence interval (CI). To account for anticipated heterogeneity, we pooled effect sizes using random effect meta-analysis, which takes into account the precision of individual studies and the variation between studies and weights each study accordingly. Heterogeneity was quantified using I^2 and T^2 statistics (see [22] for meta-analysis details).

We performed pre-specified subgroup analyses, using meta-regression, to explore sources of heterogeneity and assess the impact methodological and study quality indicators on the observed efficacy of IPC. Covariates assessed were: species, sex, comorbidity, opioid use pre-IPC, site of IPC, number of IPC cycles, duration of one ischemic cycle of IPC, total duration of IPC ischemia and duration of the delay between IPC and IR. For total duration of IPC ischemia, comparisons were stratified using increments (with increasing orders of magnitude) of $\log_{10}(0.4)$ minutes. For IPC to IR delay, comparisons were stratified using increments of $\log_{10}(0.8)$ minutes. The percentage of between-study variance explained by variables of interest was assessed using the T^2 and adjusted R^2 statistic.

We adjusted our significance level using the Holm-Bonferroni method[23] $(1-(1-\alpha)^{1/m})$ to account for multiple comparisons.

We assessed for potential publication bias by visually inspection for asymmetry in funnel plots, performing Duval and Tweedie's trim and fill analysis[24] and Egger's regression analysis[25] for small study effects.

1.7 Sensitivity analyses

To assess the robustness of our findings, sensitivity analysis was performed for the cut-off points for the subgroups of total IPC ischemia and IPC to IR delay. For total IPC ischemia, the increment between categories was changed from $\log_{10}(0.4)$ minutes to $\log_{10}(0.3)$ or $\log_{10}(0.5)$ minutes. For IPC to IR delay, the increment between categories was changed from $\log_{10}(0.8)$ minutes to $\log_{10}(0.7)$ or $\log_{10}(0.9)$ minutes.

Thirteen publications ([26-38]) reported data from a myocardial infarction model in which a coronary-to-carotid artery shunt was used for cardiac reperfusion. We decided to consider this procedure part of the infarction model, rather than a co-intervention, but investigated the effect of our decision by excluding these studies in a sensitivity analysis.

Post-hoc, we tested the robustness of our findings using the MD by re-running our analysis using the standardized difference in means (SMD).

References

1. de Vries RB, Hooijmans CR, Tillema A, Leenaars M, Ritskes-Hoitinga M. Updated version of the embase search filter for animal studies. *Laboratory animals*. 2014;48:88
2. Hooijmans CR, Tillema A, Leenaars M, Ritskes-Hoitinga M. Enhancing search efficiency by means of a search filter for finding all studies on animal experimentation in pubmed. *Laboratory animals*. 2010;44:170-175
3. Cohen MV, Yang XM, Downey JM. Conscious rabbits become tolerant to multiple episodes of ischemic preconditioning. *Circulation research*. 1994;74:998-1004
4. Galagudza MM, Syrenskii AV, Vlasov TD, Morozova LI, Egorova EI, Polumiskov VI. Effects of individual and combined administration of ethylmethylhydroxypyridine succinate and ischemic preconditioning on ischemic-reperfusive myocardial injury in rats. [russian]. *Eksperimental'naia i klinicheskaia farmakologiia*. 2009;72:22-26
5. Iliodromitis K, Farmakis D, Andreadou I, Zoga A, Bibli SI, Manolaki T, Dagres N, Iliodromitis EK, Anastasiou-Nana M, Kremastinos DT. Various models of cardiac conditioning in single or sequential periods of ischemia: Comparative effects on infarct size and intracellular signaling. *Int J Cardiol*. 2013;168:1336-1341
6. Liaudet L, Yang Z, Al-Affar EB, Szabo C. Myocardial ischemic preconditioning in rodents is dependent on poly (adp-ribose) synthetase. *Mol Med*. 2001;7:406-417
7. Liem DA, te Lintel Hekkert M, Manintveld OC, Boomsma F, Verdouw PD, Duncker DJ. Myocardium tolerant to an adenosine-dependent ischemic preconditioning stimulus can still be protected by stimuli that employ alternative signaling pathways. *American journal of physiology. Heart and circulatory physiology*. 2005;288:14

8. Shen B, Li J, Gao L, Zhang J, Yang B. Role of cc-chemokine receptor 5 on myocardial ischemia-reperfusion injury in rats. *Mol Cell Biochem*. 2013;378:137-144
9. Li Y, Kloner RA. Cardioprotective effects of ischemic preconditioning can be recaptured after they are lost. *J Am Coll Cardiol*. 1994;23:470-474
10. Sack S, Mohri M, Arras M, Schwarz ER, Schaper W. Ischaemic preconditioning--time course of renewal in the pig. *Cardiovascular research*. 1993;27:551-555
11. Vogt AM, Ando H, Arras M, Elsasser A. Lack of adenosine causes myocardial refractoriness. *J Am Coll Cardiol*. 1998;31:1134-1141
12. Li SJ, Wu YN, Kang Y, Yin YQ, Gao WZ, Liu YX, Lou JS. Effects of limb atraumatic ischemic preconditioning on apoptosis induced by myocardial ischemia-reperfusion injury in rats. [chinese]. *Chinese Pharmacological Bulletin*. 2009;25:100-104
13. Li SJ, Wu YN, Kang Y, Yin YQ, Gao WZ, Liu YX, Lou JS. Noninvasive limb ischemic preconditioning protects against myocardial i/r injury in rats. *J Surg Res*. 2010;164:162-168
14. Li SJ, Wu YN, Kang Y, Yin YQ, Gao WZ, Liu YX, Lou JS. Influence of limb atraumatic ischemic preconditioning on fibrolysis factors in myocardial ischemia/reperfusion injury in rats. [chinese]. *Chinese Pharmacological Bulletin*. 2008;24:1344-1347
15. Mullenheim J, Schlack W, Frassdorf J, Heinen A, Preckel B, Thamer V. Additive protective effects of late and early ischaemic preconditioning are mediated by the opening of katp channels in vivo. *Pflugers Arch*. 2001;442:178-187
16. Mullenheim J, Ebel D, Bauer M, Otto F, Heinen A, Frassdorf J, Preckel B, Schlack W. Sevoflurane confers additional cardioprotection after ischemic late preconditioning in rabbits. *Anesthesiology*. 2003;99:624-631
17. Wu YN, Zhu XH, Yuan HJ, Yu H, Li SJ, Kang Y, Jiao JJ, Gao WZ, Liu YX, Lou JS. Noninvasive limb ischemic preconditioning increased antioxidative ability of myocardium after ischemia-reperfusion injury in rats. [chinese]. *Chinese Pharmacological Bulletin*. 2008;24:1049-1052
18. Wu YN, Yu H, Zhu XH, Yuan HJ, Kang Y, Jiao JJ, Gao WZ, Liu YX, Lou JS. Noninvasive delayed limb ischemic preconditioning attenuates myocardial ischemia-reperfusion injury in rats by a mitochondrial k(atp) channel-dependent mechanism. *Physiol Res*. 2011;60:271-279
19. Zhu XH, Li YM, Yuan HJ, Luo Q, Wu YN, Kang Y, Jiao JJ, Gao WZ, Liu YX, Lou JS. Noninvasive limb ischemic preconditioning increased antioxidative ability of myocardium after ischemia-reperfusion injury in diabetic rats. [chinese]. *Chinese Pharmacological Bulletin*. 2009;25:239-243
20. Zhu XH, Yuan HJ, Wu YN, Kang Y, Jiao JJ, Gao WZ, Liu YX, Lou JS, Xia Z. Non-invasive limb ischemic pre-conditioning reduces oxidative stress and attenuates myocardium ischemia-reperfusion injury in diabetic rats. *Free Radic Res*. 2011;45:201-210
21. Hooijmans CR, Rovers MM, de Vries RB, Leenaars M, Ritskes-Hoitinga M, Langendam MW. Syrcle's risk of bias tool for animal studies. *BMC medical research methodology*. 2014;14:43
22. Vesterinen HM, Sena ES, Egan KJ, Hirst TC, Churolov L, Currie GL, Antonic A, Howells DW, Macleod MR. Meta-analysis of data from animal studies: A practical guide. *Journal of neuroscience methods*. 2014;221:92-102
23. Holm S. A simple sequentially rejective multiple test procedure. *Scand J Stat*. 1979;6:65-70
24. Duval S, Tweedie R. Trim and fill: A simple funnel-plot-based method of testing and adjusting for publication bias in meta-analysis. *Biometrics*. 2000;56:455-463
25. Egger M, Davey Smith G, Schneider M, Minder C. Bias in meta-analysis detected by a simple, graphical test. *Bmj*. 1997;315:629-634
26. Hatori N, Segawa D, Hinokiyama K, Kimura T, Iizuka Y, Ochi M, Tanaka S. Effects of ischemic preconditioning and synchronized coronary venous retroperfusion in an off-pump coronary artery bypass grafting model. *Artif Organs*. 2001;25:47-52
27. Hinokiyama K, Hatori N, Ochi M, Maehara T, Tanaka S. Myocardial protective effect of lidocaine during experimental off-pump coronary artery bypass grafting. *Ann Thorac Cardiovasc Surg*. 2003;9:36-42

28. Kitakaze M, Hori M, Morioka T, Minamino T, Takashima S, Sato H, Shinozaki Y, Chujo M, Mori H, Inoue M, et al. Infarct size-limiting effect of ischemic preconditioning is blunted by inhibition of 5'-nucleotidase activity and attenuation of adenosine release. *Circulation*. 1994;89:1237-1246
29. Kitakaze M, Hori M, Morioka T, Minamino T, Takashima S, Sato H, Shinozaki Y, Chujo M, Mori H, Inoue M, Kamada T. Alpha1-adrenoceptor activation mediates the infarct size-limiting effect of ischemic preconditioning through augmentation of 5'-nucleotidase activity. *Journal of Clinical Investigation*. 1994;93:2197-2205
30. Kitakaze M, Minamino T, Node K, Komamura K, Shinozaki Y, Chujo M, Mori H, Inoue M, Hori M, Kamada T. Role of activation of ectosolic 5'-nucleotidase in the cardioprotection mediated by opening of k+c channels. *The American journal of physiology*. 1996;270:H1744-1756
31. Kitakaze M, Node K, Asanuma H, Takashima S, Sakata Y, Asakura M, Sanada S, Shinozaki Y, Mori H, Kuzuya T, Hori M. Protein tyrosine kinase is not involved in the infarct size-limiting effect of ischemic preconditioning in canine hearts. *Circulation research*. 2000;87:303-308
32. Kitakaze M, Node K, Minamino T, Komamura K, Funaya H, Shinozaki Y, Chujo M, Mori H, Inoue M, Hori M, Kamada T. Role of activation of protein kinase c in the infarct size-limiting effect of ischemic preconditioning through activation of ecto-5'-nucleotidase. *Circulation*. 1996;93:781-791
33. Minamino T, Kitakaze M, Morioka T, Node K, Komamura K, Takeda H, Inoue M, Hori M, Kamada T. Cardioprotection due to preconditioning correlates with increased ecto-5'-nucleotidase activity. *The American journal of physiology*. 1996;270:H238-244
34. Node K, Kitakaze M, Minamino T, Tada M, Inoue M, Hori M, Kamada T. Activation of ecto-5'-nucleotidase by protein kinase c and its role in ischaemic tolerance in the canine heart. *British journal of pharmacology*. 1997;120:273-281
35. Sanada S, Asanuma H, Tsukamoto O, Minamino T, Node K, Takashima S, Fukushima T, Ogai A, Shinozaki Y, Fujita M, Hirata A, Okuda H, Shimokawa H, Tomoike H, Hori M, Kitakaze M. Protein kinase a as another mediator of ischemic preconditioning independent of protein kinase c. *Circulation*. 2004;110:51-57
36. Sanada S, Kitakaze M, Asanuma H, Harada K, Ogita H, Node K, Takashima S, Sakata Y, Asakura M, Shinozaki Y, Mori H, Kuzuya T, Hori M. Role of mitochondrial and sarcolemmal k(atp) channels in ischemic preconditioning of the canine heart. *American journal of physiology. Heart and circulatory physiology*. 2001;280:H256-263
37. Sanada S, Kitakaze M, Papst PJ, Hatanaka K, Asanuma H, Aki T, Shinozaki Y, Ogita H, Node K, Takashima S, Asakura M, Yamada J, Fukushima T, Ogai A, Kuzuya T, Mori H, Terada N, Yoshida K, Hori M. Role of phasic dynamism of p38 mitogen-activated protein kinase activation in ischemic preconditioning of the canine heart. *Circulation research*. 2001;88:175-180
38. Shintani Y, Node K, Asanuma H, Sanada S, Takashima S, Asano Y, Liao Y, Fujita M, Hirata A, Shinozaki Y, Fukushima T, Nagamachi Y, Okuda H, Kim J, Tomoike H, Hori M, Kitakaze M. Opening of ca2+-activated k+ channels is involved in ischemic preconditioning in canine hearts. *Journal of molecular and cellular cardiology*. 2004;37:1213-1218

Wever et al. 2015 - Determinants of the efficacy of cardiac ischemic preconditioning: a systematic review and meta-analysis of animal studies

Text B - Full search strategies

**Pubmed**	
Heart	heart[MH] OR myocardium[MH] OR heart[TIAB] OR hearts[TIAB] OR cardiac[TIAB] OR myocardial[TIAB] OR myocardium[TIAB] OR myocardia[TIAB] OR myocard[TIAB] OR cardioprotection[TIAB] OR cardioprotective[TIAB] OR coronary[TIAB]
Ischemia-Reperfusion Injury	ischemia[MH] OR "warm ischemia"[MH] OR "cold ischemia"[MH] OR "reperfusion injury"[MH] OR "myocardial ischemia"[MH] OR "coronary occlusion"[MH] OR "heart injuries"[MH] OR "cardiovascular diseases"[MH] OR transplantation[MH] OR "heart transplantation"[MH] OR "primary graft dysfunction"[MH] OR "graft survival"[MH] OR "graft rejection" [MH] OR transplants[MH] OR ((ischemia[TIAB] OR ischaemia[TIAB] OR ischemic[TIAB] OR ischaemic[TIAB]) AND ("reperfusion"[tiab] OR reoxygenation[tiab] OR re-oxygenation[tiab])) OR "reperfusion injury"[TIAB] OR "reperfusion injuries"[TIAB] OR "I/R"[TIAB] OR "IRI"[TIAB] OR occlusion[TIAB] OR transplantation[TIAB] OR transplantations[TIAB] OR transplant[TIAB] OR transplants[TIAB] OR graft[TIAB] OR grafts[TIAB]
Preconditioning	"ischemic preconditioning"[MH] OR "IPC"[TIAB] OR "RIPC"[TIAB] OR "brief ischemia"[TIAB] OR "brief ischaemia"[TIAB] OR preconditioning[TIAB] OR "pre conditioning"[TIAB] OR "pre-conditioning"[TIAB] OR "transient ischaemia"[TIAB] OR "transient ischemia"[TIAB] OR "intermittent ischaemia"[TIAB] OR "intermittent ischemia"[TIAB] OR "remote conditioning"[TIAB]
Animals	("animal experimentation"[MeSH Terms] OR "models, animal"[MeSH Terms] OR "invertebrates"[MeSH Terms] OR "Animals"[Mesh:noexp] OR "animal population groups"[MeSH Terms] OR "chordata"[MeSH Terms:noexp] OR "chordata, nonvertebrate"[MeSH Terms] OR "vertebrates"[MeSH Terms:noexp] OR "amphibians"[MeSH Terms] OR "birds"[MeSH Terms] OR "fishes"[MeSH Terms] OR "reptiles"[MeSH Terms] OR "mammals"[MeSH Terms:noexp] OR "primates"[MeSH Terms:noexp] OR "artiodactyla"[MeSH Terms] OR "carnivora"[MeSH Terms] OR "cetacea"[MeSH Terms] OR "chiroptera"[MeSH Terms] OR "elephants"[MeSH Terms] OR "hyraxes"[MeSH Terms] OR "insectivora"[MeSH Terms] OR "lagomorpha"[MeSH Terms] OR "marsupialia"[MeSH Terms] OR "monotremata"[MeSH Terms] OR "perissodactyla"[MeSH Terms] OR "rodentia"[MeSH Terms] OR "scandentia"[MeSH Terms] OR "sirenia"[MeSH Terms] OR "xenarthra"[MeSH Terms] OR "haplorhini"[MeSH Terms:noexp] OR "strepsirhini"[MeSH Terms] OR "platyrrhini"[MeSH Terms] OR "tarsii"[MeSH Terms] OR "catarrhini"[MeSH Terms:noexp] OR "cercopithecidae"[MeSH Terms] OR "hylobatidae"[MeSH Terms] OR "hominidae"[MeSH Terms:noexp] OR "gorilla gorilla"[MeSH Terms] OR "pan paniscus"[MeSH Terms] OR "pan troglodytes"[MeSH Terms] OR "pongo pygmaeus"[MeSH Terms]) OR ((animals[tiab] OR animal[tiab] OR mice[Tiab] OR mus[Tiab] OR mouse[Tiab] OR murine[Tiab] OR woodmouse[tiab] OR rats[Tiab] OR rat[Tiab] OR murinae[Tiab] OR muridae[Tiab] OR cottonrat[tiab] OR cottonrats[tiab] OR hamster[tiab] OR hamsters[tiab] OR cricetinae[tiab] OR rodentia[Tiab] OR rodent[Tiab] OR rodents[Tiab] OR pigs[Tiab] OR pig[Tiab] OR swine[tiab] OR swines[tiab] OR piglets[tiab] OR piglet[tiab] OR boar[tiab] OR boars[tiab] OR "sus scrofa"[tiab] OR ferrets[tiab] OR ferret[tiab] OR polecat[tiab] OR polecats[tiab] OR "mustela putorius"[tiab] OR "guinea pigs"[Tiab] OR "guinea

	pig"[Tiab] OR cavia[Tiab] OR callithrix[Tiab] OR marmoset[Tiab] OR marmosets[Tiab] OR cebuella[Tiab] OR hapale[Tiab] OR octodon[Tiab] OR chinchilla[Tiab] OR chinchillas[Tiab] OR gerbillinae[Tiab] OR gerbil[Tiab] OR gerbils[Tiab] OR jird[Tiab] OR jirds[Tiab] OR merione[Tiab] OR meriones[Tiab] OR rabbits[Tiab] OR rabbit[Tiab] OR hares[Tiab] OR hare[Tiab] OR diptera[Tiab] OR flies[Tiab] OR fly[Tiab] OR dipteral[Tiab] OR drosophila[Tiab] OR drosophilidae[Tiab] OR cats[Tiab] OR cat[Tiab] OR carus[Tiab] OR felis[Tiab] OR nematoda[Tiab] OR nematode[Tiab] OR nematodes[Tiab] OR sipunculida[Tiab] OR dogs[Tiab] OR dog[Tiab] OR canine[Tiab] OR canines[Tiab] OR canis[Tiab] OR sheep[Tiab] OR sheeps[Tiab] OR mouflon[Tiab] OR mouflons[Tiab] OR ovis[Tiab] OR goats[Tiab] OR goat[Tiab] OR capra[Tiab] OR capras[Tiab] OR rupicapra[Tiab] OR rupicapras[Tiab] OR chamois[Tiab] OR haplorhini[Tiab] OR monkey[Tiab] OR monkeys[Tiab] OR anthropoidea[Tiab] OR anthropoids[Tiab] OR saguinus[Tiab] OR tamarin[Tiab] OR tamarins[Tiab] OR leontopithecus[Tiab] OR hominidae[Tiab] OR ape[Tiab] OR apes[Tiab] OR "pan paniscus"[Tiab] OR bonobo[Tiab] OR bonobos[Tiab] OR "pan troglodytes"[Tiab] OR gibbon[Tiab] OR gibbons[Tiab] OR siamang[Tiab] OR siamangs[Tiab] OR nomascus[Tiab] OR symphalangus[Tiab] OR chimpanzee[Tiab] OR chimpanzees[Tiab] OR prosimian[Tiab] OR prosimians[Tiab] OR "bush baby"[Tiab] OR bush babies[Tiab] OR galagos[Tiab] OR galago[Tiab] OR pongidae[Tiab] OR gorilla[Tiab] OR gorillas[Tiab] OR "pongo pygmaeus"[Tiab] OR orangutan[Tiab] OR orangutans[Tiab] OR lemur[Tiab] OR lemurs[Tiab] OR lemuridae[Tiab] OR horse[Tiab] OR horses[Tiab] OR equus[Tiab] OR cow[Tiab] OR calf[Tiab] OR bull[Tiab] OR chicken[Tiab] OR chickens[Tiab] OR gallus[Tiab] OR quail[Tiab] OR bird[Tiab] OR birds[Tiab] OR quails[Tiab] OR poultry[Tiab] OR poultries[Tiab] OR fowl[Tiab] OR fowls[Tiab] OR reptile[Tiab] OR reptilia[Tiab] OR reptiles[Tiab] OR snakes[Tiab] OR snake[Tiab] OR lizard[Tiab] OR lizards[Tiab] OR alligator[Tiab] OR alligators[Tiab] OR crocodile[Tiab] OR crocodiles[Tiab] OR turtle[Tiab] OR turtles[Tiab] OR amphibian[Tiab] OR amphibians[Tiab] OR amphibia[Tiab] OR frog[Tiab] OR frogs[Tiab] OR bombina[Tiab] OR salientia[Tiab] OR toad[Tiab] OR toads[Tiab] OR "epidalea calamita"[Tiab] OR salamander[Tiab] OR salamanders[Tiab] OR eel[Tiab] OR eels[Tiab] OR fish[Tiab] OR fishes[Tiab] OR pisces[Tiab] OR catfish[Tiab] OR catfishes[Tiab] OR siluriformes[Tiab] OR arius[Tiab] OR heteropneustes[Tiab] OR sheatfish[Tiab] OR perch[Tiab] OR perches[Tiab] OR percidae[Tiab] OR perca[Tiab] OR trout[Tiab] OR trouts[Tiab] OR char[Tiab] OR chars[Tiab] OR salvelinus[Tiab] OR minnow[Tiab] OR cyprinidae[Tiab] OR carps[Tiab] OR carp[Tiab] OR zebrafish[Tiab] OR zebrafishes[Tiab] OR goldfish[Tiab] OR goldfishes[Tiab] OR guppy[Tiab] OR guppies[Tiab] OR chub[Tiab] OR chubs[Tiab] OR tinca[Tiab] OR barbels[Tiab] OR barbus[Tiab] OR pimephales[Tiab] OR promelas[Tiab] OR "poecilia reticulata"[Tiab] OR mullet[Tiab] OR mullets[Tiab] OR eel[Tiab] OR eels[Tiab] OR seahorse[Tiab] OR seahorses[Tiab] OR mugil curema[Tiab] OR atlantic cod[Tiab] OR shark[Tiab] OR sharks[Tiab] OR catshark[Tiab] OR anguilla[Tiab] OR salmonid[Tiab] OR salmonids[Tiab] OR whitefish[Tiab] OR whitefishes[Tiab] OR salmon[Tiab] OR salmons[Tiab] OR sole[Tiab] OR solea[Tiab] OR lamprey[Tiab] OR lampreys[Tiab] OR pumpkinseed[Tiab] OR sunfish[Tiab] OR sunfishes[Tiab] OR tilapia[Tiab] OR tilapias[Tiab] OR turbot[Tiab] OR turbots[Tiab] OR flatfish[Tiab] OR flatfishes[Tiab] OR sciuridae[Tiab] OR squirrel[Tiab] OR squirrels[Tiab] OR chipmunk[Tiab] OR chipmunks[Tiab] OR suslik[Tiab] OR susliks[Tiab] OR vole[Tiab] OR voles[Tiab] OR lemming[Tiab] OR lemmings[Tiab] OR muskrat[Tiab] OR muskrats[Tiab] OR lemmus[Tiab] OR otter[Tiab] OR otters[Tiab] OR marten[Tiab] OR martens[Tiab] OR martes[Tiab] OR weasel[Tiab] OR badger[Tiab] OR badgers[Tiab] OR ermine[Tiab] OR mink[Tiab] OR minks[Tiab] OR sable[Tiab] OR

	sables[Tiab] OR gulo[Tiab] OR gulos[Tiab] OR wolverine[Tiab] OR wolverines[Tiab] OR mustela[Tiab] OR llama[Tiab] OR llamas[Tiab] OR alpaca[Tiab] OR alpacas[Tiab] OR camelid[Tiab] OR camelids[Tiab] OR guanaco[Tiab] OR guanacos[Tiab] OR chiroptera[Tiab] OR chiropteras[Tiab] OR bat[Tiab] OR bats[Tiab] OR fox[Tiab] OR foxes[Tiab] OR iguana[Tiab] OR iguanas[Tiab] OR xenopus laevis[Tiab] OR parakeet[Tiab] OR parakeets[Tiab] OR parrot[Tiab] OR parrots[Tiab] OR donkey[Tiab] OR donkeys[Tiab] OR mule[Tiab] OR mules[Tiab] OR zebra[Tiab] OR zebras[Tiab] OR shrew[Tiab] OR shrews[Tiab] OR bison[Tiab] OR bisons[Tiab] OR buffalo[Tiab] OR buffaloes[Tiab] OR deer[Tiab] OR deers[Tiab] OR bear[Tiab] OR bears[Tiab] OR panda[Tiab] OR pandas[Tiab] OR “wild hog”[Tiab] OR “wild boar”[Tiab] OR fitchew[Tiab] OR fitch[Tiab] OR beaver[Tiab] OR beavers[Tiab] OR jerboa[Tiab] OR jerboas[Tiab] OR capybara[Tiab] OR capybaras[Tiab]) NOT medline[sb])

EMBASE	
Heart	exp heart/ or (heart or hearts or cardiac or myocardial or myocardium or myocardia or myocard or cardioprotection or cardioprotective or coronary).ti,ab.
Ischemia-Reperfusion Injury	exp reperfusion injury/ or exp cold ischemia/ or exp primary graft dysfunction/ or exp transplantation/ or (((ischemia or ischaemia or ischemia or ischaemia) and (reperfusion or reoxygenation or re-oxygenation)) or reperfusion injury or reperfusion injuries or I/r or IRI or occlusion or transplantation or transplantations or transplant or transplants or graft or grafts).ti,ab.
Preconditioning	exp ischemic preconditioning/ or (IPC or RIPC or brief ischemia or brief ischaemia or preconditioning or pre conditioning or pre-conditioning or transient ischaemia or transient ischemia or intermittent ischaemia or intermittent ischemia or remote conditioning).ti,ab.
Animals	exp animal experiment/ or exp animal model/ or exp experimental animal/ or exp transgenic animal/ or exp male animal/ or exp female animal/ or exp juvenile animal/ OR animal/ OR chordata/ OR vertebrate/ OR tetrapod/ OR exp fish/ OR amniote/ OR exp amphibia/ OR mammal/ OR exp reptile/ OR exp sauropsid/ OR therian/OR exp monotremate/ OR placental mammals/ OR exp marsupial/ OR Euarchontoglires/ OR exp Afrotheria/ OR exp Boreoeutheria/ OR exp Laurasiatheria/ OR exp Xenarthra/ OR primate/ OR exp Dermoptera/ OR exp Glires/ OR exp Scandentia/ OR Haplorhini/ OR exp prosimian/ OR simian/ OR exp tarsiiform/ OR Catarrhini/ OR exp Platyrrhini/ OR ape/ OR exp Cercopithecidae/ OR hominid/ OR exp hylobatidae/ OR exp chimpanzee/ OR exp gorilla/ OR exp orang utan/ OR (animal OR animals OR pisces OR fish OR fishes OR catfish OR catfishes OR sheatfish OR silurus OR arius OR heteropneustes OR clarias OR gariepinus OR fathead minnow OR fathead minnows OR pimephales OR promelas OR cichlidae OR trout OR trouts OR char OR chars OR salvelinus OR salmo OR oncorhynchus OR guppy OR guppies OR millionfish OR poecilia OR goldfish OR goldfishes OR carassius OR auratus OR mullet OR mullets OR mugil OR curema OR shark OR sharks OR cod OR cods OR gadus OR morhua OR carp OR carps OR cyprinus OR carpio OR killifish OR eel OR eels OR anguilla OR zander OR sander OR lucioperca OR stizostedion OR turbot OR turbots OR psetta OR flatfish OR flatfishes OR plaice OR pleuronectes OR platessa OR tilapia OR tilapias OR oreochromis OR sarotherodon OR common sole OR dover sole OR solea OR zebrafish OR zebrafishes OR danio OR rerio OR seabass OR dicentrarchus OR labrax OR morone OR lamprey OR lampreys OR petromyzon OR pumpkinseed OR

	pumpkinseeds OR lepomis OR gibbosus OR herring OR clupea OR harengus OR amphibia OR amphibian OR amphibians OR anura OR salientia OR frog OR frogs OR rana OR toad OR toads OR bufo OR xenopus OR laevis OR bombina OR epidalea OR calamita OR salamander OR salamanders OR newt OR newts OR triturus OR reptilia OR reptile OR reptiles OR bearded dragon OR pogona OR vitticeps OR iguana OR iguanas OR lizard OR lizards OR anguis fragilis OR turtle OR turtles OR snakes OR snake OR aves OR bird OR birds OR quail OR quails OR coturnix OR bobwhite OR colinus OR virginianus OR poultry OR poultries OR fowl OR fowls OR chicken OR chickens OR gallus OR zebra finch OR taeniopygia OR guttata OR canary OR canaries OR serinus OR canaria OR parakeet OR parakeets OR grasskeet OR parrot OR parrots OR psittacine OR psittacines OR shelduck OR tadorna OR goose OR geese OR branta OR leucopsis OR woodlark OR lullula OR flycatcher OR ficedula OR hypoleuca OR dove OR doves OR geopelia OR cuneata OR duck OR ducks OR greylag OR graylag OR anser OR harrier OR circus pygargus OR red knot OR great knot OR calidris OR canutus OR godwit OR limosa OR lapponica OR meleagris OR gallopavo OR jackdaw OR corvus OR monedula OR ruff OR philomachus OR pugnax OR lapwing OR peewit OR plover OR vanellus OR swan OR cygnus OR columbianus OR bewickii OR gull OR chroicocephalus OR ridibundus OR albifrons OR great tit OR parus OR aythya OR fuligula OR streptopelia OR risoria OR spoonbill OR platalea OR leucorodia OR blackbird OR turdus OR merula OR blue tit OR cyanistes OR pigeon OR pigeons OR columba OR pintail OR anas OR starling OR sturnus OR owl OR athene noctua OR pochard OR ferina OR cockatiel OR nymphicus OR hollandicus OR skylark OR alauda OR tern OR sterna OR teal OR crecca OR oystercatcher OR haematopus OR ostralegus OR shrew OR shrews OR sorex OR araneus OR crocidura OR russula OR european mole OR talpa OR chiroptera OR bat OR bats OR eptesicus OR serotinus OR myotis OR dasycneme OR daubentonii OR pipistrelle OR pipistrellus OR cat OR cats OR felis OR catus OR feline OR dog OR dogs OR canis OR canine OR canines OR otter OR otters OR lutra OR badger OR badgers OR meles OR fitchew OR fitch OR foumart or foulmart OR ferrets OR ferret OR polecat OR polecats OR mustela OR putorius OR weasel OR weasels OR fox OR foxes OR vulpes OR common seal OR phoca OR vitulina OR grey seal OR halichoerus OR horse OR horses OR equus OR equine OR equidae OR donkey OR donkeys OR mule OR mules OR pig OR pigs OR swine OR swines OR hog OR hogs OR boar OR boars OR porcine OR piglet OR piglets OR sus OR scrofa OR llama OR llamas OR lama OR glama OR deer OR deers OR cervus OR elaphus OR cow OR cows OR bos taurus OR bos indicus OR bovine OR bull OR bulls OR cattle OR bison OR bisons OR sheep OR sheeps OR ovis aries OR ovine OR lamb OR lambs OR mouflon OR mouflons OR goat OR goats OR capra OR caprine OR chamois OR rupicapra OR leporidae OR lagomorpha OR lagomorph OR rabbit OR rabbits OR oryctolagus OR cuniculus OR laprine OR hares OR lepus OR rodentia OR rodent OR rodents OR murinae OR mouse OR mice OR mus OR musculus OR murine OR woodmouse OR apodemus OR rat OR rats OR rattus OR norvegicus OR guinea pig OR guinea pigs OR cavia OR porcellus OR hamster OR hamsters OR mesocricetus OR cricetulus OR cricetus OR gerbil OR gerbils OR jird OR jirds OR meriones OR unguiculatus OR jerboa OR jerboas OR jaculus OR chinchilla OR chinchillas OR beaver OR beavers OR castor fiber OR castor canadensis OR sciuridae OR squirrel OR squirrels OR sciurus OR chipmunk OR chipmunks OR marmot OR marmots OR marmota OR suslik OR susliks OR spermophilus OR cynomys OR cottonrat OR cottonrats OR sigmodon OR vole OR voles OR microtus OR myodes OR glareolus OR primate OR primates OR prosimian OR prosimians OR lemur OR lemurs OR lemuridae OR loris OR bush baby OR bush babies OR bushbaby OR bushbabies OR galago OR galagos OR anthropoidea OR

	anthropoids OR simian OR simians OR monkey OR monkeys OR marmoset OR marmosets OR callithrix OR cebuella OR tamarin OR tamarins OR saguinus OR leontopithecus OR squirrel monkey OR squirrel monkeys OR saimiri OR night monkey OR night monkeys OR owl monkey OR owl monkeys OR douroucoulis OR aotus OR spider monkey OR spider monkeys OR ateles OR baboon OR baboons OR papio OR rhesus monkey OR macaque OR macaca OR mulatta OR cynomolgus OR fascicularis OR green monkey OR green monkeys OR chlorocebus OR vervet OR vervets OR pygerythrus OR hominoidea OR ape OR apes OR hylobatidae OR gibbon OR gibbons OR siamang OR siamangs OR nomascus OR symphalangus OR hominidae OR orangutan OR orangutans OR pongo OR chimpanzee OR chimpanzees OR pan troglodytes OR bonobo OR bonobos OR pan paniscus OR gorilla OR gorillas OR troglodytes).ti,ab.

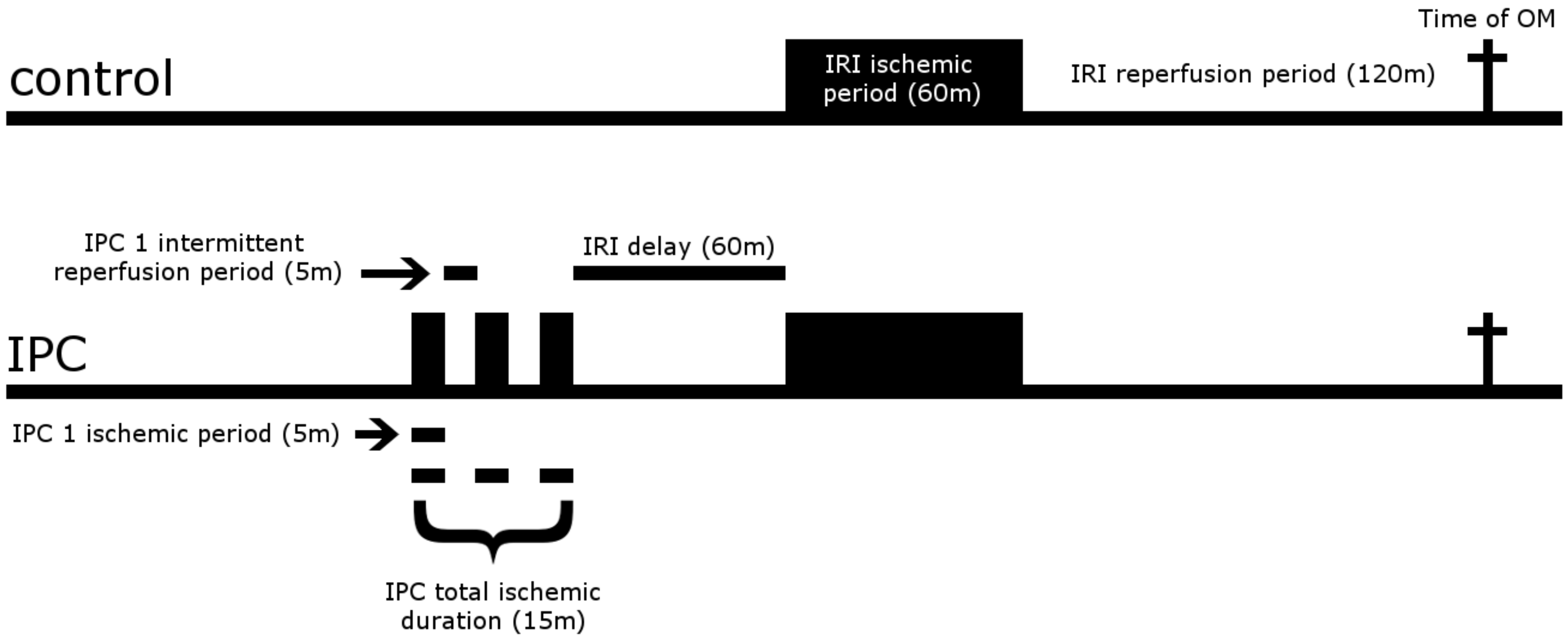

Supplemental Figure A| Ischemia-reperfusion injury (IRI) and ischemic preconditioning (IPC) characteristics for a typical control and experimental group. Control animals undergo a waiting period, followed by 60 minutes of coronary occlusion (ischemic period) and 120 minutes of reperfusion. In the experimental group, the applied IPC protocol consists of 3 cycles of 5 minutes of ischemia, interspersed by 5 minutes of reperfusion. The total duration of IPC ischemia is 15 minutes. The delay to myocardial IRI in this protocol is 60 minutes.

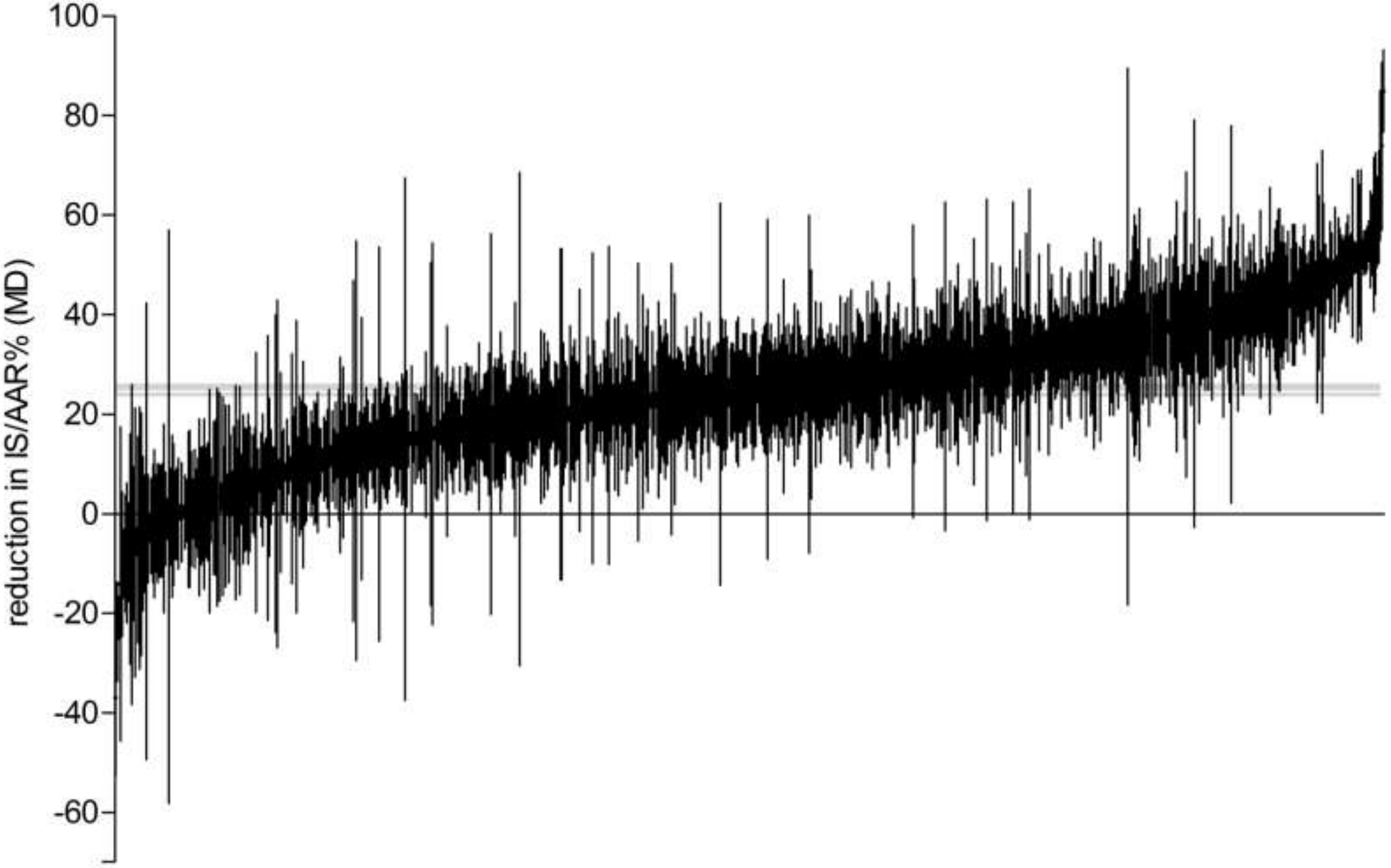

Supplemental Figure B| Individual effect estimates of all 785 included comparisons. Data are presented as raw difference in means (MD) in infarct size as a percentage of the area at risk (IS/AAR%). Comparisons are sorted by MD from low to high. The vertical error bars represent the 95% confidence intervals for the individual estimates. Horizontal white line and grey bar represent the pooled effect estimate and its 95% confidence interval.

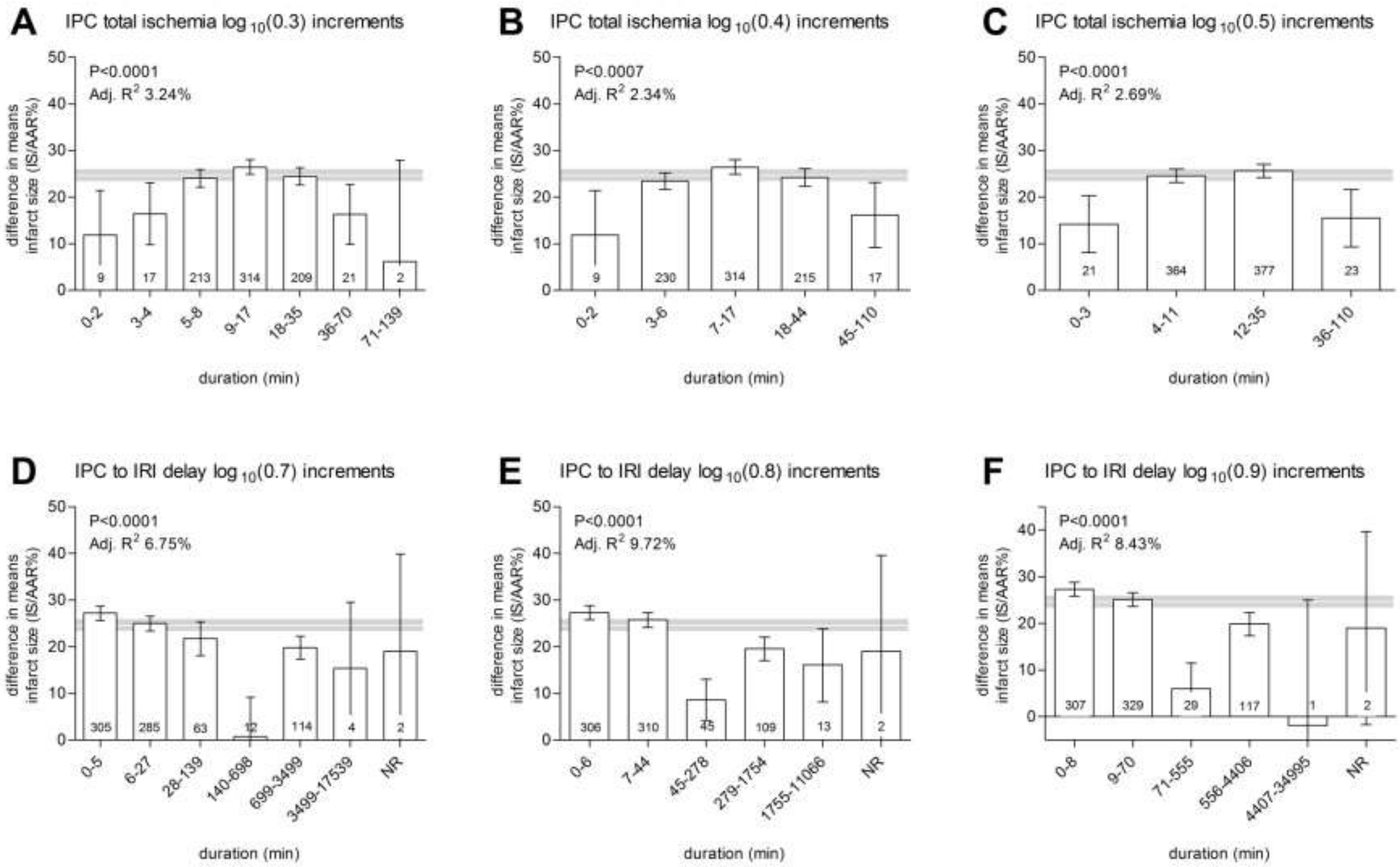

Supplemental Figure C| Sensitivity analysis total duration IPC ischemia (A-C) and IPC to IRI delay (D-F). Data are presented as raw difference in means (MD) in infarct size as a percentage of the area at risk (IS/AAR%). Horizontal white line and grey bar represent the pooled effect estimate and its 95% confidence interval. The number of comparisons contributing data is indicated in each bar. Panels B and D represent the original subgroups, other panels represent alternative subgroups based on slightly smaller (A and D) or larger (C and F) log increments.

Supplemental table A - study and comparison characteristics - page 1 of 26

comparison	species	opioids pre-IPC	sex (m/f)	condition / comorbidity	site of IPC	remote organ	IPC # ischemic cycli	IPC ischemic cycle (min)	IPC total ischemia (min)	IPC to IRI delay (min)
Alcindor2004[1]	dog	NR	m/f	none	local	NA	4	5	20	5
Alizadeh2011[2]	rat	NR	m	none	local	NA	1	5	5	5
Alkhulaifi1993_ip0.5[3]	rat	NR	m	none	local	NA	1	5	5	0.5
Alkhulaifi1993_ip1[3]	rat	NR	m	none	local	NA	1	5	5	1
Amour2009[4]	rabbit	NR	m	none	local	NA	1	5	5	15
Andreadou2004[5]	rabbit	NR	m	none	local	NA	2	5	10	10
Andreadou2006[6]	rabbit	NR	m	none	local	NA	2	5	10	10
Andreadou2011_1cy[7]	rabbit	NR	m	none	local	NA	1	5	5	10
Andreadou2011_4cy[7]	rabbit	NR	m	none	local	NA	4	4	16	10
Aouam2005_1cy[8]	rabbit	NR	m	none	local	NA	1	5	5	10
Aouam2005_6cy[8]	rabbit	NR	m	none	local	NA	6	4	24	4
Argaud2004[9]	rabbit	yes	m	none	local	NA	1	5	5	5
Argaud2005a[10]	rabbit	yes	m	none	local	NA	1	5	5	5
Argaud2005b[11]	rabbit	NR	m	none	local	NA	1	5	5	5
Argaud2005c[12]	rabbit	NR	m	none	local	NA	1	5	5	5
Argaud2008[13]	rabbit	NR	m	none	local	NA	1	5	5	5
Auchampach1992[14]	dog	NR	m/f	none	local	NA	1	5	5	10
Auchampach1993[15]	dog	NR	m/f	none	local	NA	1	5	5	10
Auchampach2004[16]	dog	NR	m/f	none	local	NA	4	5	20	10
Aye1999_1cy[17]	rat	NR	m	none	local	NA	1	3	3	10
Aye1999_3cy[17]	rat	NR	m	none	local	NA	3	3	9	5
Baines1997_1cy[18]	rabbit	NR	m/f	none	local	NA	1	5	5	10
Baines1997_4cy[18]	rabbit	NR	m/f	none	local	NA	4	5	20	10
Baines1999[19]	rabbit	NR	m/f	none	local	NA	1	5	5	10
Barbosa1996_01.25ip[20]	rat	NR	f	none	local	NA	1	1	1	5
Barbosa1996_02.5ip[20]	rat	NR	f	none	local	NA	1	3	3	5
Barbosa1996_05ip[20]	rat	NR	f	none	local	NA	1	5	5	5
Barbosa1996_10ip[20]	rat	NR	f	none	local	NA	1	10	10	5
Barbosa1996_2cy[20]	rat	NR	f	none	local	NA	2	5	10	5
Barbosa1996_2cy10ip[20]	rat	NR	f	none	local	NA	2	10	20	5
Barbosa1996_4cy[20]	rat	NR	f	none	local	NA	4	5	20	5

comparison	species	opioids pre-IPC	sex (m/f)	condition / comorbidity	site of IPC	remote organ	IPC # ischemic cycli	IPC ischemic cycle (min)	IPC total ischemia (min)	IPC to IRI delay (min)
Basalay2012_exp1[21]	rat	NR	m	none	remote	hind limb	1	15	15	10
Basalay2012_exp2[21]	rat	NR	m	none	remote	hind limb	1	15	15	10
Basalay2012_exp3[21]	rat	NR	m	none	remote	hind limb	1	15	15	10
Baumert2007[22]	pig	NR	NR	none	local	NA	4	5	20	5
Baxter1994[23]	rabbit	yes	m	none	local	NA	4	5	20	1440
Baxter1995[24]	rabbit	yes	m	none	local	NA	4	5	20	1440
Baxter1997_1cy[25]	rabbit	yes	m	none	local	NA	1	5	5	2880
Baxter1997_2cy[25]	rabbit	yes	m	none	local	NA	2	5	10	2880
Baxter1997_4cy[25]	rabbit	yes	m	none	local	NA	4	5	20	2880
Baxter1997_ip24h[25]	rabbit	yes	m	none	local	NA	4	5	20	1440
Baxter1997_ip48h[25]	rabbit	yes	m	none	local	NA	4	5	20	2880
Baxter1997_ip72h[25]	rabbit	yes	m	none	local	NA	4	5	20	4320
Baxter1997_ip96h[25]	rabbit	yes	m	none	local	NA	4	5	20	5760
Belosjorow1999[26]	rabbit	NR	NR	none	local	NA	1	5	5	10
Belosjorow2003[27]	rabbit	NR	NR	none	local	NA	1	5	5	10
Bencsik2010[28]	rat	NR	NR	none	local	NA	5	4	20	1440
Bernardo1999[29]	rabbit	NR	m	none	local	NA	4	5	20	1440
Bibli2013[30]	rabbit	NR	m	none	local	NA	2	5	10	10
Blokhin2008a[31]	rat	NR	m	none	local	NA	1	5	5	5
Blokhin2008b_20is[32]	rat	NR	m	none	local	NA	3	5	15	5
Blokhin2008b_30is[32]	rat	NR	m	none	local	NA	3	5	15	5
Blokhin2008b_40is[32]	rat	NR	m	none	local	NA	3	5	15	5
Blokhin2008b_50is[32]	rat	NR	m	none	local	NA	3	5	15	5
Blokhin2008b_60is[32]	rat	NR	m	none	local	NA	3	5	15	5
Boengler2007_old[33]	mouse	NR	m/f	senescence	local	NA	1	10	10	10
Boengler2007_young[33]	mouse	NR	m/f	none	local	NA	1	10	10	10
Brandenburger2012[34]	rat	NR	NR	none	remote	hind limb	4	5	20	5
Bukhari1995[35]	sheep	NR	m/f	none	local	NA	3	5	15	5
Burckhartt1995_ip2h[36]	rabbit	NR	NR	none	local	NA	1	5	5	120
Burckhartt1995_ip4h[36]	rabbit	NR	NR	none	local	NA	1	5	5	240
Burns1996_adult[37]	sheep	NR	m/f	none	local	NA	3	5	15	5

comparison	species	opioids pre-IPC	sex (m/f)	condition / comorbidity	site of IPC	remote organ	IPC # ischemic cycli	IPC ischemic cycle (min)	IPC total ischemia (min)	IPC to IRI delay (min)
Burns1996_old[37]	sheep	NR	m/f	senescence	local	NA	3	5	15	5
Cai2008[38]	mouse	NR	m	none	local	NA	1	10	10	5
Cai2013[39]	mouse	NR	m	none	remote	hind limb	3	5	15	1440
Canyon2005[40]	rat	NR	m	none	local	NA	3	3	9	3
Cason1997[41]	rabbit	NR	NR	none	local	NA	1	5	5	15
Chazov2001[42]	rat	NR	m	none	local	NA	3	5	15	5
Chen1997[43]	rabbit	NR	m	none	local	NA	4	5	20	10
Chen2005[44]	rat	NR	NR	none	remote	hind limb	4	5	20	5
Chen2008[45]	rat	NR	m	none	local	NA	2	3	6	5
Cheng2009[46]	rat	NR	m	none	local	NA	3	5	15	5
Cheng2010a[47]	rat	NR	m	none	local	NA	4	5	20	5
Cheng2010b[48]	rat	NR	m	none	local	NA	4	5	20	5
Chiari2002[49]	rabbit	yes	m/f	brain death	local	NA	2	5	10	10
Chien1996[50]	rabbit	NR	m	none	local	NA	1	5	5	10
Chien1999[51]	rabbit	NR	m	none	local	NA	1	5	5	10
Cohen1991[52]	rabbit	NR	NR	none	local	NA	1	5	5	10
Cohen1994_1cy[53]	rabbit	none	NR	none	local	NA	1	5	5	10
Cohen1994_rep[53]	rabbit	none	NR	none	local	NA	12	5	60	10
Cohen1994_rep+1cy[53]	rabbit	none	NR	none	local	NA	12	5	60	10
Cohen1999[54]	rabbit	yes	m/f	none	local	NA	1	5	5	10
Cohen2000[55]	rabbit	yes	m/f	none	local	NA	1	5	5	10
Colantonio2004[56]	dog	NR	NR	none	local	NA	1	10	10	10
Dai2003[57]	rat	NR	f	none	local	NA	3	3	9	5
Dai2009_healthy[58]	rat	NR	f	none	local	NA	3	3	9	5
Dai2009_hyperten[58]	rat	NR	f	hypertension	local	NA	3	3	9	5
Dairaku2002[59]	rat	NR	m	none	local	NA	3	3	9	3
Daleau2001_120is[60]	rabbit	NR	m	none	local	NA	2	5	10	10
Daleau2001_30is[60]	rabbit	NR	m	none	local	NA	2	5	10	10
Das2006[61]	rabbit	NR	m	none	local	NA	1	5	5	10
Das2007[62]	rabbit	NR	m	none	local	NA	1	5	5	10
Das2012[63]	rabbit	NR	m	none	local	NA	1	5	5	10

Supplemental table A - study and comparison characteristics - page 4 of 26

comparison	species	opioids pre-IPC	sex (m/f)	condition / comorbidity	site of IPC	remote organ	IPC # ischemic cycli	IPC ischemic cycle (min)	IPC total ischemia (min)	IPC to IRI delay (min)
Dawn2002[64]	rabbit	NR	m	none	local	NA	6	4	24	1440
Dawn2004a[65]	mouse	NR	NR	none	local	NA	6	4	24	1440
Dawn2004b[66]	mouse	NR	NR	none	local	NA	6	4	24	1440
Demiryurek2005a[67]	rat	NR	m	none	local	NA	1	5	5	5
Demiryurek2005b[68]	rat	NR	m	none	local	NA	1	5	5	5
DePaulis2013[69]	rat	NR	m	none	local	NA	3	3	9	5
Depre2010_ip24h[70]	pig	NR	NR	none	local	NA	2	10	20	1440
Depre2010_ip60[70]	pig	NR	NR	none	local	NA	6	10	60	60
Deuchar2007[71]	rat	NR	m	none	local	NA	3	3	9	5
DeZeeuw2001_10ip[72]	pig	NR	m/f	none	remote	brain	1	10	10	20
DeZeeuw2001_30ip[72]	pig	NR	m/f	none	remote	brain	1	30	30	30
Ding2000[73]	rabbit	NR	m	none	local	NA	2	5	10	10
Ding2001[74]	rabbit	NR	m	none	remote	RAO	1	10	10	10
Dong2004[75]	rat	NR	m	none	remote	hind limb	1	10	10	10
Ebel2003_diabetic[76]	rabbit	NR	m	diabetes	local	NA	1	5	5	1440
Ebel2003_healthy1[76]	rabbit	NR	m	none	local	NA	1	5	5	1440
Ebel2003_healthy2[76]	rabbit	NR	m	none	local	NA	1	5	5	1440
Ebel2003_hypergly[76]	rabbit	NR	m	hyperglycaemia	local	NA	1	5	5	1440
Ebel2009_diabetic[77]	rat	NR	m	diabetes	local	NA	3	3	9	5
Ebel2009_healthy[77]	rat	NR	m	none	local	NA	3	3	9	5
Eckle2006_1cy[78]	mouse	NR	m/f	none	local	NA	1	5	5	5
Eckle2006_2cy[78]	mouse	NR	m/f	none	local	NA	2	5	10	5
Eckle2006_3cy[78]	mouse	NR	m/f	none	local	NA	3	5	15	5
Eckle2006_4cy[78]	mouse	NR	m/f	none	local	NA	4	5	20	5
Eckle2012[79]	mouse	NR	m	none	local	NA	4	5	20	5
Erikson1996[80]	dog	NR	NR	none	local	NA	4	5	20	5
Fang2008[81]	rat	NR	m	none	local	NA	3	5	15	5
Farhat2001_braindead[82]	rabbit	NR	NR	brain death	local	NA	1	5	5	5
Farhat2001_healthy[82]	rabbit	NR	NR	none	local	NA	1	5	5	5
Fisher2002_ip15m[83]	mouse	NR	m	none	local	NA	3	5	15	15
Fisher2002_ip24h[83]	mouse	NR	m	none	local	NA	3	5	15	1440

comparison	species	opioids pre-IPC	sex (m/f)	condition / comorbidity	site of IPC	remote organ	IPC # ischemic cycli	IPC ischemic cycle (min)	IPC total ischemia (min)	IPC to IRI delay (min)
Flack3rd1991[84]	pig	NR	m/f	none	local	NA	4	5	20	5
Flaherty2008[85]	mouse	NR	m	none	local	NA	6	4	24	4
Flynn2005_1cy[86]	rabbit	NR	m	none	local	NA	1	5	5	10
Flynn2005_2cy[86]	rabbit	NR	m	none	local	NA	1	5	5	10
Fryer1998[87]	rat	NR	m	none	local	NA	1	5	5	5
Fryer1999_1cy[88]	rat	NR	m	none	local	NA	1	5	5	5
Fryer1999_3cy[88]	rat	NR	m	none	local	NA	3	5	15	5
Fryer2000[89]	rat	NR	m	none	local	NA	1	5	5	5
Fryer2001a[90]	rat	NR	m	none	local	NA	1	5	5	5
Fryer2001b[91]	rat	NR	m	none	local	NA	1	5	5	5
Fryer2001c[92]	rat	NR	m	none	local	NA	1	5	5	5
Fryer2002[93]	rat	NR	m	none	local	NA	1	5	5	5
Fukuma2000[94]	rabbit	NR	m	none	local	NA	1	5	5	5
Galagudza2007a_diabetic[95]	rat	NR	m	diabetes	local	NA	1	5	5	5
Galagudza2007a_healthy[95]	rat	NR	m	none	local	NA	1	5	5	5
Galagudza2007b_1cy[96]	rat	NR	m	none	local	NA	1	5	5	5
Galagudza2007b_3cy[96]	rat	NR	m	none	local	NA	3	5	15	5
Galagudza2007b_RIPC[96]	rat	NR	m	none	remote	MAO	1	5	5	15
Galagudza2009[97]	rat	NR	m	none	local	NA	3	4	12	5
Gao2000[98]	mouse	NR	m/f	none	local	NA	3	5	15	10
Gao2003_ip10[99]	rat	NR	m	none	local	NA	3	5	15	10
Gao2003_ip24h[99]	rat	NR	m	none	local	NA	3	5	15	1440
Gao2012_IPC[100]	rat	NR	m	none	local	NA	3	5	15	5
Gao2012_RIPC[100]	rat	NR	m	none	remote	hind limb	3	5	15	5
Ge2011_exp1[101]	mouse	NR	NR	hyperglycaemia	local	NA	4	5	20	5
Ge2011_exp2[101]	mouse	NR	NR	hyperglycaemia	local	NA	4	5	20	5
Gerczuk2012[102]	rat	NR	m	none	local	NA	3	3	9	5
Gho1996_IPC[103]	rat	NR	m	none	local	NA	1	15	15	10
Gho1996_MAO[103]	rat	NR	m	none	remote	MAO	1	15	15	10
Gho1996_RAO[103]	rat	NR	m	none	remote	RAO	1	15	15	10
Gomez2011[104]	mouse	yes	m	none	local	NA	1	5	5	5

Supplemental table A - study and comparison characteristics - page 6 of 26

comparison	species	opioids pre-IPC	sex (m/f)	condition / comorbidity	site of IPC	remote organ	IPC # ischemic cycli	IPC ischemic cycle (min)	IPC total ischemia (min)	IPC to IRI delay (min)
Gomoll1996_02ip[105]	ferret	NR	m	none	local	NA	1	2	2	10
Gomoll1996_05ip[105]	ferret	NR	m	none	local	NA	1	5	5	10
Gomoll1996_10ip[105]	ferret	NR	m	none	local	NA	1	10	10	10
Gomoll1996_75is[105]	ferret	NR	m	none	local	NA	1	5	5	10
Gomoll1996_90is[105]	ferret	NR	m	none	local	NA	1	5	5	10
Goto1993[106]	rabbit	NR	m	none	local	NA	2	2	4	5
Gourine2005[107]	rat	NR	m	none	local	NA	3	5	15	5
Gozal2005[108]	rabbit	NR	NR	none	local	NA	1	5	5	10
Griol-Charhbili2005[109]	mouse	NR	m	none	local	NA	3	3	9	5
Gross1992[110]	dog	NR	m/f	none	local	NA	1	5	5	10
Gross2009[111]	dog	NR	m/f	none	local	NA	1	5	5	10
Grover1992[112]	dog	NR	m/f	none	local	NA	1	5	5	10
Grover1996[113]	dog	NR	m/f	none	local	NA	1	5	5	10
Grund1997_1cy[114]	pig	NR	m/f	none	local	NA	1	10	10	30
Grund1997_2cy[114]	pig	NR	m/f	none	local	NA	2	10	20	30
Gu2008[115]	dog	NR	m/f	hyperglycaemia	local	NA	4	5	20	5
Gumina1999_1cy[116]	dog	NR	m/f	none	local	NA	1	5	5	10
Gumina1999_4cy[116]	dog	NR	m/f	none	local	NA	4	5	20	5
Gumina1999_60is[116]	dog	NR	m/f	none	local	NA	1	5	5	10
Gumina2001[117]	dog	NR	m/f	none	local	NA	1	5	5	10
Gumina2005_1cy[118]	dog	NR	m/f	none	local	NA	1	10	10	10
Gumina2005_1cyip2h[118]	dog	NR	m/f	none	local	NA	1	10	10	120
Gumina2005_2cy[118]	dog	NR	m/f	none	local	NA	2	10	20	10
Guo1998_early[119]	mouse	NR	m	none	local	NA	6	4	24	10
Guo1998_late24hR[119]	mouse	NR	m	none	local	NA	6	4	24	1440
Guo1998_late4R[119]	mouse	NR	m	none	local	NA	6	4	24	1440
Guo1999_ip10[120]	mouse	NR	m	none	local	NA	6	4	24	10
Guo1999_ip24h[120]	mouse	NR	m	none	local	NA	6	4	24	1440
Guo2001_1cy[121]	mouse	NR	m	none	local	NA	1	4	4	10
Guo2001_6cy[121]	mouse	NR	m	none	local	NA	6	4	24	4
Guo2007[122]	rat	NR	NR	none	local	NA	2	5	10	5

comparison	species	opioids pre-IPC	sex (m/f)	condition / comorbidity	site of IPC	remote organ	IPC # ischemic cycli	IPC ischemic cycle (min)	IPC total ischemia (min)	IPC to IRI delay (min)
Guo2012_exp1[123]	mouse	NR	m	none	local	NA	6	4	24	1440
Guo2012_exp2[123]	mouse	NR	m	none	local	NA	6	4	24	1440
Guo2012_exp3[123]	mouse	NR	m	none	local	NA	6	4	24	1440
Gysembergh1998[124]	rabbit	yes	NR	none	local	NA	1	5	5	5
Haessler1994_iso[125]	rabbit	NR	NR	none	local	NA	1	5	5	10
Haessler1994_kxy[125]	rabbit	NR	NR	none	local	NA	1	5	5	10
Haessler1994_pen[125]	rabbit	NR	NR	none	local	NA	1	5	5	10
Haessler1997[126]	rabbit	NR	NR	none	local	NA	1	5	5	10
Hale1992_exp1[127]	rabbit	NR	m	none	local	NA	2	5	10	5
Hale1992_exp2[127]	rabbit	NR	m	none	local	NA	2	5	10	5
Hale1999_1cy[128]	rabbit	NR	NR	none	local	NA	1	5	5	5
Hale1999_2cy[128]	rabbit	NR	NR	none	local	NA	2	7	14	5
Halkos2004[129]	dog	yes	NR	none	local	NA	1	5	5	10
Hampton2003[130]	mouse	NR	NR	none	local	NA	3	5	15	1440
Haruna1998[131]	rabbit	NR	f	none	local	NA	1	5	5	10
Hatori2001[132]	pig	NR	m/f	none	local	NA	1	5	5	15
Hausenloy2012[133]	pig	yes	NR	none	remote	hind limb	4	5	20	5
He2009[134]	rabbit	NR	f	none	local	NA	3	5	15	5
Heinen2011_IPC[135]	rat	NR	m	none	local	NA	4	5	20	5
Heinen2011_IPC+RIPC[135]	rat	NR	m	none	local+remote	hind limb	4	5	20	5
Heinen2011_RIPC[135]	rat	NR	m	none	remote	hind limb	4	5	20	5
HemetsbergerH2008[136]	pig	NR	NR	none	local	NA	2	5	10	5
HemetsbergerR2008[137]	pig	NR	NR	none	local	NA	2	5	10	5
Hiasa2001[138]	rat	NR	m	none	local	NA	4	5	20	10
Hibert2013[139]	rat	NR	m	none	remote	hind limb	1	10	10	10
Hide1996[140]	rabbit	yes	m	none	local	NA	1	5	5	15
Hinokiyama2003[141]	pig	NR	m/f	none	local	NA	1	5	5	15
Hiroi2010_1ip[142]	rat	NR	m	none	local	NA	4	1	4	1440
Hiroi2010_3ip[142]	rat	NR	m	none	local	NA	4	3	12	1440
Hoshida1994[143]	dog	NR	m/f	none	local	NA	4	5	20	5
Htun1998[144]	pig	yes	m	none	local	NA	2	10	20	30

comparison	species	opioids pre-IPC	sex (m/f)	condition / comorbidity	site of IPC	remote organ	IPC # ischemic cycli	IPC ischemic cycle (min)	IPC total ischemia (min)	IPC to IRI delay (min)
Hu2002_adult[145]	rat	NR	m	none	remote	MAO	6	4	24	1440
Hu2002_old[145]	rat	NR	m	senescence	remote	MAO	6	4	24	1440
Huang1999[146]	pig	NR	NR	none	local	NA	2	10	20	10
Huang2004[147]	rabbit	NR	NR	none	local	NA	2	10	20	10
Huang2011[148]	rabbit	NR	NR	none	local	NA	2	10	20	10
Huang2013[149]	rat	NR	NR	none	local	NA	2	10	20	10
Iliodromitis1994_1cy10[150]	rabbit	NR	m/f	none	local	NA	1	5	5	10
Iliodromitis1994_1cy60[150]	rabbit	NR	m/f	none	local	NA	1	5	5	60
Iliodromitis1994_2cy[150]	rabbit	NR	m/f	none	local	NA	2	5	10	60
Iliodromitis1994_3cy[150]	rabbit	NR	m/f	none	local	NA	3	5	15	10
Iliodromitis1996a_1ip[151]	rabbit	NR	NR	none	local	NA	1	1	1	10
Iliodromitis1996a_5ip[151]	rabbit	NR	NR	none	local	NA	1	5	5	10
Iliodromitis1996b_2cy45[152]	rabbit	NR	NR	none	local	NA	2	5	10	10
Iliodromitis1996b_2cy60[152]	rabbit	NR	NR	none	local	NA	2	5	10	10
Iliodromitis1996b_2cy75[152]	rabbit	NR	NR	none	local	NA	2	5	10	10
Iliodromitis1996b_ip60[152]	rabbit	NR	NR	none	local	NA	1	5	5	60
Iliodromitis1996b_ip65[152]	rabbit	NR	NR	none	local	NA	1	5	5	65
Iliodromitis1996b_ip70[152]	rabbit	NR	NR	none	local	NA	1	5	5	70
Iliodromitis1996b_ip75[152]	rabbit	NR	NR	none	local	NA	1	5	5	75
Iliodromitis1996b_ip80[152]	rabbit	NR	NR	none	local	NA	1	5	5	80
Iliodromitis1997_1cy[153]	rabbit	NR	NR	none	local	NA	1	5	5	10
Iliodromitis1997_2cy[153]	rabbit	NR	NR	none	local	NA	2	5	10	10
Iliodromitis1997_4cy[153]	rabbit	NR	NR	none	local	NA	4	5	20	10
Iliodromitis1997_6cy[153]	rabbit	NR	NR	none	local	NA	6	5	30	10
Iliodromitis1997_8cy[153]	rabbit	NR	NR	none	local	NA	8	5	40	10
Iliodromitis2002a_1cy[154]	rabbit	NR	m	none	local	NA	1	5	5	10
Iliodromitis2002a_3cy[154]	rabbit	NR	m	none	local	NA	3	5	15	10
Iliodromitis2002b[155]	rabbit	NR	m	none	local	NA	1	5	5	10
Iliodromitis2003_3cy[156]	rabbit	NR	m	none	local	NA	3	5	15	10
Iliodromitis2003_3cy90[156]	rabbit	NR	m	none	local	NA	3	5	15	90
Iliodromitis2003_8cy[156]	rabbit	NR	m	none	local	NA	8	5	40	10

comparison	species	opioids pre-IPC	sex (m/f)	condition / comorbidity	site of IPC	remote organ	IPC # ischemic cycli	IPC ischemic cycle (min)	IPC total ischemia (min)	IPC to IRI delay (min)
Iliodromitis2004[157]	rabbit	NR	m	none	local	NA	3	5	15	10
Iliodromitis2006a[158]	rabbit	NR	m	none	local	NA	2	5	10	10
Iliodromitis2006b_exp1[159]	rabbit	NR	m	hypercholesterolemia	local	NA	2	5	10	10
Iliodromitis2006b_exp2[159]	rabbit	NR	m	hypercholesterolemia	local	NA	2	5	10	10
Iliodromitis2008_3cy[160]	rabbit	NR	m	none	local	NA	3	5	15	10
Iliodromitis2008_8cy[160]	rabbit	NR	m	none	local	NA	8	5	40	10
Iliodromitis2013_20ip[161]	rabbit	NR	m	none	local	NA	1	20	20	12
Iliodromitis2013_5ip+20ip[161]	rabbit	NR	m	none	local	NA	3	10	30	12
Iliodromitis2013_5ip20is[161]	rabbit	NR	m	none	local	NA	2	5	10	10
Iliodromitis2013_5ip40is[161]	rabbit	NR	m	none	local	NA	2	5	10	10
Imagawa1997[162]	rabbit	yes	m	none	local	NA	4	5	20	2880
Imagawa1998[163]	rabbit	yes	m	none	local	NA	1	5	5	10
Imagawa1999[164]	rabbit	yes	m	none	local	NA	4	5	20	2880
Ismaeil1999[165]	rabbit	NR	NR	none	local	NA	1	5	5	15
Iwamoto1991[166]	rabbit	NR	m	none	local	NA	4	5	20	5
Jaberansari2001_2cy[167]	pig	NR	m/f	none	local	NA	2	5	10	1440
Jaberansari2001_4cy[167]	pig	NR	m/f	none	local	NA	4	5	20	1440
Jancso2004_2cy[168]	pig	NR	m/f	none	local	NA	2	2	4	1440
Jancso2004_4cy[168]	pig	NR	m/f	none	local	NA	4	5	20	1440
Jansco2005[169]	rabbit	NR	NR	none	local	NA	4	5	20	1440
Ji2013_diabetic[170]	rat	NR	m	diabetes	local	NA	2	5	10	5
Ji2013_healthy[170]	rat	NR	m	none	local	NA	2	5	10	5
Jiao2002[171]	rat	NR	m	none	local	NA	2	5	10	10
Jung2000_healthy[172]	rabbit	NR	m	none	local	NA	2	5	10	10
Jung2000_hyperchol[172]	rabbit	NR	m	hypercholesterolemia	local	NA	2	5	10	10
Junhua1997[173]	rabbit	NR	NR	none	local	NA	4	5	20	1440
Kalakech2013_mouse[174]	mouse	NR	m	none	remote	hind limb	4	5	20	5
Kalakech2013_rat[174]	rat	NR	m	none	remote	hind limb	4	5	20	5
Kapadia1997[175]	pig	NR	NR	none	local	NA	2	10	20	15
Kara 2004[176]	rat	NR	m	none	local	NA	1	5	5	5
Kara2006[177]	rat	NR	m	none	local	NA	1	5	5	5

comparison	species	opioids pre-IPC	sex (m/f)	condition / comorbidity	site of IPC	remote organ	IPC # ischemic cycli	IPC ischemic cycle (min)	IPC total ischemia (min)	IPC to IRI delay (min)
Kariya1997[178]	rabbit	NR	m	none	local	NA	1	5	5	5
Katakam2007_lean[179]	rat	NR	m	none	local	NA	1	5	5	5
Katakam2007_obese[179]	rat	NR	m	obese	local	NA	1	5	5	5
Kersten1997[180]	dog	NR	NR	none	local	NA	4	5	20	5
Kersten1998[181]	dog	NR	NR	hyperglycaemia	local	NA	4	5	20	5
Kersten2000_diabetic[182]	dog	NR	NR	diabetes	local	NA	4	5	20	5
Kersten2000_healthy[182]	dog	NR	NR	none	local	NA	4	5	20	5
Kingma1999[183]	rabbit	NR	m	none	local	NA	2	5	10	10
Kingma2011[184]	dog	yes	m/f	none	remote	RAO	4	5	20	5
Kingma2013[185]	dog	yes	m/f	none	local	NA	4	5	20	5
Kirsch2000_braindead[186]	rabbit	NR	NR	brain death	local	NA	1	3	3	3
Kirsch2000_healthy[186]	rabbit	NR	NR	none	local	NA	1	3	3	3
Kis2003[187]	rabbit	yes	m	none	local	NA	4	5	20	1440
Kitakaze1994a[188]	dog	NR	NR	none	local	NA	4	5	20	5
Kitakaze1994b_anesth[189]	dog	NR	NR	none	local	NA	4	5	20	5
Kitakaze1994b_concious[189]	dog	NR	NR	none	local	NA	4	5	20	5
Kitakaze1996a[190]	dog	NR	NR	none	local	NA	4	5	20	5
Kitakaze1996b[191]	dog	NR	NR	none	local	NA	4	5	20	5
Kitakaze2000[192]	dog	NR	NR	none	local	NA	4	5	20	5
Kocoglu2008[193]	rat	NR	m	none	local	NA	1	5	5	5
Kohler2007[194]	mouse	NR	NR	none	local	NA	4	5	20	5
Kojima1995[195]	rat	NR	m	none	local	NA	1	5	5	10
Kolettis2013[196]	rat	NR	m	none	local	NA	2	5	10	10
Koneru2007[197]	rat	NR	m	none	local	NA	4	4	16	4
Koning1994a_ip120[198]	pig	NR	NR	none	local	NA	1	10	10	120
Koning1994a_ip15[198]	pig	NR	NR	none	local	NA	1	10	10	15
Koning1994a_ip180[198]	pig	NR	NR	none	local	NA	1	10	10	180
Koning1994a_ip240[198]	pig	NR	NR	none	local	NA	1	10	10	240
Koning1994a_ip60[198]	pig	NR	NR	none	local	NA	1	10	10	60
Koning1994b_ip15[199]	pig	NR	NR	none	local	NA	1	10	10	15
Koning1994b_ip60[199]	pig	NR	NR	none	local	NA	1	10	10	60

comparison	species	opioids pre-IPC	sex (m/f)	condition / comorbidity	site of IPC	remote organ	IPC # ischemic cycli	IPC ischemic cycle (min)	IPC total ischemia (min)	IPC to IRI delay (min)
Kositprapa2001[200]	rabbit	NR	m	none	local	NA	4	5	20	1440
Kouchi1998[201]	rabbit	NR	f	none	local	NA	1	5	5	10
Kremastinos2000_healthy[202]	rabbit	NR	m	none	local	NA	1	5	5	10
Kremastinos2000_hyperchol[202]	rabbit	NR	m	hypercholesterolemia	local	NA	1	5	5	10
Kristensen2004[203]	pig	NR	NR	none	local	NA	2	10	20	30
Kudej2006_ip10[204]	pig	yes	NR	none	local	NA	2	10	20	10
Kudej2006_ip24h[204]	pig	yes	NR	none	local	NA	2	10	20	1440
Kunuthur2012_exp1[205]	mouse	NR	m	none	local	NA	1	5	5	5
Kunuthur2012_exp2[205]	mouse	NR	m	none	local	NA	1	5	5	5
Kuzmin2000[206]	rat	NR	m	none	local	NA	3	5	15	5
Kuzume2004_1cy[207]	rabbit	NR	NR	none	local	NA	1	5	5	10
Kuzume2004_2cy[207]	rabbit	NR	NR	none	local	NA	2	5	10	10
Kuzuya1993_ip03h[208]	dog	NR	m/f	none	local	NA	4	5	20	1440
Kuzuya1993_ip05[208]	dog	NR	m/f	none	local	NA	4	5	20	5
Kuzuya1993_ip12h[208]	dog	NR	m/f	none	local	NA	4	5	20	180
Kuzuya1993_ip24h[208]	dog	NR	m/f	none	local	NA	4	5	20	720
Landim2013_healthy[209]	rat	NR	m	none	local	NA	3	3	9	3
Landim2013_hyperchol[209]	rat	NR	m	hypercholesterolemia	local	NA	3	3	9	3
Lang2006_IPC[210]	rat	NR	m	none	local	NA	3	5	15	5
Lang2006_RIPC[210]	rat	NR	m	none	remote	RAO	1	10	10	20
Lange2006[211]	rabbit	NR	m	none	local	NA	1	5	5	30
Lankford2006[212]	mouse	NR	m	none	local	NA	3	5	15	10
Larsen2007[213]	pig	NR	f	none	local	NA	2	5	10	5
Lasley1995[214]	rabbit	NR	m/f	none	local	NA	1	5	5	10
Lazou2006[215]	rabbit	NR	m	none	local	NA	1	5	5	10
Lee2000[216]	dog	NR	NR	none	local	NA	1	5	5	10
Lee2004_bif1cy[217]	rabbit	NR	NR	none	local	NA	1	5	5	10
Lee2004_bif3cy[217]	rabbit	NR	NR	none	local	NA	3	5	15	10
Lee2004_bif5cy[217]	rabbit	NR	NR	none	local	NA	5	5	25	10
Lee2004_bif7cy[217]	rabbit	NR	NR	none	local	NA	7	5	35	10
Lee2004_trif1cy[217]	rabbit	NR	NR	none	local	NA	1	5	5	10

comparison	species	opioids pre-IPC	sex (m/f)	condition / comorbidity	site of IPC	remote organ	IPC # ischemic cycli	IPC ischemic cycle (min)	IPC total ischemia (min)	IPC to IRI delay (min)
Lee2004_trif3cy[217]	rabbit	NR	NR	none	local	NA	3	5	15	10
Lee2004_trif5cy[217]	rabbit	NR	NR	none	local	NA	5	5	25	10
Li_G1990_01cy[218]	dog	NR	m	none	local	NA	1	5	5	10
Li_G1990_06cy[218]	dog	NR	m	none	local	NA	6	5	30	10
Li_G1990_12cy[218]	dog	NR	m	none	local	NA	12	5	60	10
Li_Q2003[219]	mouse	NR	m	none	local	NA	6	4	24	1440
Li_R2009[220]	rat	NR	m	none	local	NA	3	5	15	5
Li_S2008_IPC[221]	rat	NR	NR	none	local	NA	3	5	15	5
Li_S2008_RIPC[221]	rat	NR	NR	none	remote	hind limb	9	5	45	1440
Li_S2009_IPC[222]	rat	NR	NR	none	local	NA	3	5	15	5
Li_S2009_RIPC[222]	rat	NR	NR	none	remote	hind limb	9	5	45	1440
Li_S2010_IPC[223]	rat	NR	m	none	local	NA	3	5	15	5
Li_S2010_RIPC[223]	rat	NR	m	none	remote	hind limb	9	5	45	1440
Li_W2003[224]	rabbit	NR	m	none	local	NA	4	5	20	1440
Li_X2012a[225]	pig	NR	NR	none	local	NA	3	5	15	5
Li_X2012b[226]	pig	NR	m/f	none	local	NA	3	5	15	5
Li_Y1992a_ip05[227]	rat	NR	f	none	local	NA	3	3	9	5
Li_Y1992a_ip1h[227]	rat	NR	f	none	local	NA	3	3	9	60
Li_Y1992a_ip2h[227]	rat	NR	f	none	local	NA	3	3	9	120
Li_Y1992a_ip3h[227]	rat	NR	f	none	local	NA	3	3	9	180
Li_Y1992b[228]	rat	NR	f	none	local	NA	3	5	15	5
Li_Y1993a[229]	rat	NR	f	none	local	NA	3	3	9	5
Li_Y1993b[230]	rat	NR	f	none	local	NA	3	3	9	5
Li_Y1994_ip05[231]	rat	NR	f	none	local	NA	3	3	9	5
Li_Y1994_ip1h[231]	rat	NR	f	none	local	NA	3	3	9	60
Li_Y1994_ip60ip[231]	rat	NR	f	none	local	NA	6	3	18	5
Li_Y1995[232]	rat	NR	f	none	local	NA	3	3	9	5
Li_Y2011[233]	mouse	NR	m	none	local	NA	3	5	15	1440
Liao2013_convent[234]	mouse	NR	m	none	local	NA	3	5	15	5
Liao2013_lungrec[234]	mouse	NR	m	none	local	NA	3	5	15	5
Liaudet2001_mouse[235]	mouse	NR	NR	none	local	NA	4	5	20	10

comparison	species	opioids pre-IPC	sex (m/f)	condition / comorbidity	site of IPC	remote organ	IPC # ischemic cycli	IPC ischemic cycle (min)	IPC total ischemia (min)	IPC to IRI delay (min)
Liaudet2001_rat[235]	rat	NR	m	none	local	NA	4	5	20	5
Lie2010[236]	pig	NR	f	none	local	NA	2	10	20	30
Liem2001_1cy[237]	rat	NR	m	none	local	NA	1	15	15	10
Liem2001_3cy[237]	rat	NR	m	none	local	NA	3	3	9	3
Liem2005_01cyip70[238]	rat	NR	m	none	local	NA	1	15	15	70
Liem2005_04cyip70[238]	rat	NR	m	none	local	NA	4	15	60	70
Liem2005_2cy[238]	rat	NR	m	none	local	NA	2	15	30	10
Liem2005_3cy[238]	rat	NR	m	none	local	NA	3	3	9	5
Liem2005_6cy[238]	rat	NR	m	none	local	NA	6	15	90	10
Liem2005_7cy[238]	rat	NR	m	none	local	NA	7	10	70	5
Liem2005_ip010[238]	rat	NR	m	none	local	NA	1	15	15	10
Liem2005_ip090[238]	rat	NR	m	none	local	NA	1	15	15	90
Liem2005_ip175[238]	rat	NR	m	none	local	NA	1	15	15	175
Liem2005_IPC+RIPC[238]	rat	NR	m	none	local+remote	MAO	6	15	90	10
Liem2005_RIPC[238]	rat	NR	m	none	remote	MAO	2	15	30	10
Lim2007_30is[239]	mouse	NR	m/f	none	local	NA	1	5	5	5
Lim2007_45is[239]	mouse	NR	m/f	none	local	NA	1	5	5	5
Lim2010[240]	mouse	NR	m	none	remote	hind limb	3	5	15	5
Liu1993a[241]	rat	NR	m	diabetes	local	NA	3	5	15	5
Liu1993b[242]	rat	NR	m	none	local	NA	3	5	15	5
Liu1994[243]	rabbit	NR	m/f	none	local	NA	1	5	5	10
Liu2005[244]	rat	NR	m	none	local	NA	3	5	15	1440
Liu2008[245]	rat	NR	m	none	local	NA	5	4	20	4
LiuG1992a[246]	rabbit	NR	m/f	none	local	NA	1	5	5	10
LiuG1992b[247]	rabbit	NR	m	none	local	NA	1	5	5	10
LiuY1992_1cy[248]	rat	NR	m	none	local	NA	1	5	5	10
LiuY1992_3cy[248]	rat	NR	m	none	local	NA	3	5	15	5
Loke1998[249]	dog	NR	m/f	none	local	NA	3	5	15	10
Lott1996[250]	rat	NR	m	none	local	NA	2	5	10	5
Lotz2011[251]	rabbit	NR	m	none	remote	RAO	3	10	30	10
Lou2003[252]	rat	none	m	none	local	NA	3	5	15	5

comparison	species	opioids pre-IPC	sex (m/f)	condition / comorbidity	site of IPC	remote organ	IPC # ischemic cycli	IPC ischemic cycle (min)	IPC total ischemia (min)	IPC to IRI delay (min)
Lu2009[253]	mouse	NR	NR	none	local	NA	3	5	15	5
Lu2011[254]	rat	NR	m	none	local	NA	3	5	15	5
Lu2012_1cy[255]	rat	NR	m	none	remote	hind limb	1	5	5	5
Lu2012_3cy[255]	rat	NR	m	none	remote	hind limb	3	5	15	5
Luo2004[256]	rat	NR	m	none	local	NA	4	3	12	1440
Manintveld2005[257]	rat	NR	m	none	local	NA	1	15	15	10
Manintveld2011_1cy[258]	rat	NR	NR	none	local	NA	1	15	15	10
Manintveld2011_3cy[258]	rat	NR	NR	none	local	NA	3	3	9	5
Marber1993[259]	rabbit	yes	NR	none	local	NA	4	5	20	1440
Martin1997[260]	pig	NR	m/f	none	local	NA	1	10	10	10
Matsuda1993[261]	dog	NR	m/f	none	local	NA	4	5	20	5
Matsumura2000[262]	rat	NR	m	none	local	NA	1	3	3	10
McVey1999a[263]	rat	NR	m	none	local	NA	3	3	9	5
McVey1999b[264]	dog	NR	NR	none	local	NA	1	5	5	10
Mei1998[265]	dog	NR	NR	none	local	NA	1	5	5	10
Miki1996[266]	rabbit	NR	m	none	local	NA	1	2	2	5
Miki1998a[267]	rabbit	NR	m/f	none	local	NA	1	5	5	10
Miki1998b[268]	rabbit	NR	m	none	local	NA	1	5	5	5
Miki1999_histo[269]	rabbit	none	m/f	none	local	NA	4	5	20	1440
Miki1999_TTC[269]	rabbit	none	m/f	none	local	NA	4	5	20	1440
Miller1999_2cy[270]	mouse	NR	NR	none	local	NA	3	5	15	5
Miller1999_3cy[270]	mouse	NR	NR	none	local	NA	2	5	10	5
Minamino1996_ip005[271]	dog	NR	m/f	none	local	NA	4	5	20	5
Minamino1996_ip030[271]	dog	NR	m/f	none	local	NA	4	5	20	30
Minamino1996_ip060[271]	dog	NR	m/f	none	local	NA	4	5	20	60
Minamino1996_ip120[271]	dog	NR	m/f	none	local	NA	4	5	20	120
Miura1991_1cy[272]	rabbit	NR	m	none	local	NA	1	5	5	5
Miura1991_2cy[272]	rabbit	NR	m	none	local	NA	2	5	10	5
Miura1991_2ip[272]	rabbit	NR	m	none	local	NA	2	2	4	5
Miura1992[273]	rabbit	NR	m	none	local	NA	1	2	2	5
Miura1998_1cy[274]	rabbit	NR	m	none	local	NA	1	5	5	5

comparison	species	opioids pre-IPC	sex (m/f)	condition / comorbidity	site of IPC	remote organ	IPC # ischemic cycli	IPC ischemic cycle (min)	IPC total ischemia (min)	IPC to IRI delay (min)
Miura1998_2cy[274]	rabbit	NR	m	none	local	NA	2	5	10	5
Mizumura1995[275]	dog	NR	m/f	none	local	NA	1	5	5	10
Mizumura1996_2.5ip[276]	dog	NR	m/f	none	local	NA	1	3	3	10
Mizumura1996_5ip[276]	dog	NR	m/f	none	local	NA	1	5	5	10
Mizumura1997_2.5ip[277]	dog	NR	m/f	none	local	NA	1	3	3	10
Mizumura1997_5ip[277]	dog	NR	m/f	none	local	NA	1	5	5	10
Morgan1999[278]	rabbit	NR	NR	none	local	NA	3	5	15	120
Mortensen2006[279]	pig	none	NR	none	local	NA	2	10	20	30
Mullenheim2001a[280]	rabbit	NR	m	none	local	NA	1	5	5	10
Mullenheim2001b[281]	rabbit	NR	m	none	local	NA	1	5	5	1440
Mullenheim2001c_ip10[282]	rabbit	NR	m	none	local	NA	1	5	5	10
Mullenheim2001c_ip24h[282]	rabbit	NR	m	none	local	NA	1	5	5	1440
Mullenheim2001c_ip24hip[282]	rabbit	NR	m	none	local	NA	2	5	10	10
Mullenheim2003_ip24h[283]	rabbit	none	m	none	local	NA	1	5	5	1440
Mullenheim2003_ip24hip[283]	rabbit	none	m	none	local	NA	2	5	10	10
Munch-Ellingsen1997[284]	rabbit	NR	m/f	none	local	NA	1	5	5	10
Munch-Ellingsen1998[285]	rabbit	NR	m/f	none	local	NA	1	5	5	10
Murry1986_3his[286]	dog	NR	m/f	none	local	NA	4	5	20	5
Murry1986_40is[286]	dog	NR	m/f	none	local	NA	4	5	20	5
Murry1991_ip120[287]	dog	NR	m/f	none	local	NA	1	15	15	120
Murry1991_ip5[287]	dog	NR	m/f	none	local	NA	1	15	15	5
Nadtochiy2009[288]	mouse	NR	m	none	local	NA	3	5	15	5
Nakae2000_2.5ip[289]	rabbit	NR	m	none	local	NA	1	3	3	20
Nakae2000_5ip[289]	rabbit	NR	m	none	local	NA	1	5	5	20
Nakano1997_3ip[290]	rabbit	NR	m	none	local	NA	1	3	3	5
Nakano1997_5ip[290]	rabbit	NR	m	none	local	NA	1	5	5	5
Naumenko2010[291]	rat	NR	m	none	local	NA	3	3	9	3
Nawada1997[292]	rabbit	NR	f	none	local	NA	1	5	5	10
Neckar2002_2cy[293]	rat	NR	m	none	local	NA	2	5	10	5
Neckar2002_5cy[293]	rat	NR	m	none	local	NA	5	5	25	5
Nieszner2002_diabetic[294]	rabbit	NR	m	diabetes	local	NA	3	2	6	2

comparison	species	opioids pre-IPC	sex (m/f)	condition / comorbidity	site of IPC	remote organ	IPC # ischemic cycli	IPC ischemic cycle (min)	IPC total ischemia (min)	IPC to IRI delay (min)
Nieszner2002_healthy[294]	rabbit	NR	m	none	local	NA	3	2	6	2
Nishihara2006[295]	rat	NR	m	none	local	NA	1	5	5	5
Nithipatikom2006a[296]	dog	NR	m/f	none	local	NA	1	5	5	10
Nithipatikom2006b[297]	dog	NR	m/f	none	local	NA	1	5	5	10
Node1997[298]	dog	NR	m/f	none	local	NA	4	5	20	5
Nozawa1999[299]	rabbit	NR	NR	none	local	NA	1	2	2	5
Nozawa2003_ip20[300]	rat	NR	m	none	local	NA	2	5	10	20
Nozawa2003_ip5[300]	rat	NR	m	none	local	NA	2	5	10	5
Okamura1999[301]	rat	NR	m	none	local	NA	3	3	9	3
Okubo2000[302]	rabbit	NR	m	none	local	NA	4	5	20	1440
Okubo2004a[303]	rabbit	NR	m	none	local	NA	4	5	20	10
Okubo2004b_ip10[304]	rabbit	NR	m	none	local	NA	4	5	20	10
Okubo2004b_ip24h[304]	rabbit	NR	m	none	local	NA	4	5	20	1440
Ovize1992_60is[305]	dog	yes	m/f	none	local	NA	4	3	12	5
Ovize1992_90is[305]	dog	yes	m/f	none	local	NA	4	3	12	5
Ovize1995_05ip[306]	pig	NR	m/f	none	local	NA	1	5	5	10
Ovize1995_10ip[306]	pig	NR	m/f	none	local	NA	1	10	10	10
Pagel2007[307]	rabbit	NR	m	none	local	NA	3	5	15	5
Patel2001[308]	rat	NR	m	none	local	NA	1	5	5	5
Patel2002_1cy[309]	rat	NR	m	none	local	NA	1	15	15	5
Patel2002_3cy[309]	rat	NR	m	none	local	NA	3	5	15	5
Patel2005[310]	rat	NR	m	none	local	NA	3	5	15	1440
Peart2003[311]	dog	NR	NR	none	local	NA	1	5	5	10
Pell1998[312]	rabbit	yes	m	none	remote	RAO	1	10	10	10
Perricone2013[313]	mouse	NR	m	none	local	NA	3	5	15	5
Petrischev2006_IPC[314]	rat	NR	NR	none	local	NA	4	3	12	6
Petrischev2006_RIPC[314]	rat	NR	NR	none	remote	MAO	1	20	20	15
Ping1999[315]	rabbit	NR	NR	none	local	NA	6	4	24	1440
Ping2002[316]	mouse	NR	NR	none	local	NA	6	4	24	1440
Piot1997[317]	rat	NR	f	none	local	NA	5	5	25	5
Poulsen2014[318]	pig	NR	f	none	local	NA	2	10	20	30

comparison	species	opioids pre-IPC	sex (m/f)	condition / comorbidity	site of IPC	remote organ	IPC # ischemic cycli	IPC ischemic cycle (min)	IPC total ischemia (min)	IPC to IRI delay (min)
Przyklenk1993[319]	dog	yes	NR	none	remote	LCX	4	5	20	5
Przyklenk1995[320]	dog	NR	NR	none	local	NA	4	5	20	5
Przyklenk1996[321]	dog	NR	NR	none	local	NA	4	5	20	5
Przyklenk1997[322]	dog	NR	NR	none	local	NA	1	10	10	30
Przyklenk2001_adult[323]	rabbit	NR	NR	none	local	NA	1	5	5	10
Przyklenk2001_middle[323]	rabbit	NR	NR	middle-age	local	NA	1	5	5	10
Przyklenk2001_old[323]	rabbit	NR	NR	senescence	local	NA	1	5	5	10
Przyklenk2003_adult[324]	rabbit	NR	m/f	none	local	NA	1	5	5	10
Przyklenk2003_old[324]	rabbit	NR	m/f	senescence	local	NA	1	5	5	10
Qian1996[325]	rat	NR	NR	none	local	NA	1	5	5	5
Qian1999_1cy[326]	rat	NR	m	none	local	NA	1	5	5	10
Qian1999_1cy24h[326]	rat	NR	m	none	local	NA	1	5	5	1440
Qian1999_2cy24h[326]	rat	NR	m	none	local	NA	2	5	10	1440
Qian1999_3cy[326]	rat	NR	m	none	local	NA	3	5	15	10
Qian1999_3cy24h[326]	rat	NR	m	none	local	NA	3	5	15	1440
Qian1999_3cy48h[326]	rat	NR	m	none	local	NA	3	5	15	2880
Qiu1997a[327]	rabbit	NR	m	none	local	NA	6	4	24	1440
Qiu1997b_25cy[328]	pig	NR	m/f	none	local	NA	25	2	50	1440
Qiu1997b_ip24h[328]	pig	NR	m/f	none	local	NA	10	2	20	1440
Qiu1997b_ip25[328]	pig	NR	m/f	none	local	NA	10	2	20	25
Rajesh2003_ip10[329]	rat	NR	m	cardiac hypertrophy	local	NA	4	3	12	10
Rajesh2003_ip24h[329]	rat	NR	m	cardiac hypertrophy	local	NA	4	3	12	1440
Rajesh2003_ip5h[329]	rat	NR	m	cardiac hypertrophy	local	NA	4	3	12	300
Rajesh2004_healthy[330]	rat	NR	m	none	local	NA	4	3	12	10
Rajesh2004_hypertr[330]	rat	NR	m	cardiac hypertrophy	local	NA	4	3	12	10
Raphael2005[331]	rabbit	NR	NR	none	local	NA	1	5	5	10
Redel2009_3cy[332]	mouse	NR	m	none	local	NA	3	5	15	40
Redel2009_6cy[332]	mouse	NR	m	none	local	NA	6	5	30	40
Reffelmann2003[333]	rabbit	NR	m	none	local	NA	1	5	5	10
Ren2004[334]	mouse	NR	m/f	none	local	NA	6	4	24	1440
Richard1993[335]	rat	NR	m	none	local	NA	3	5	15	5

comparison	species	opioids pre-IPC	sex (m/f)	condition / comorbidity	site of IPC	remote organ	IPC # ischemic cycli	IPC ischemic cycle (min)	IPC total ischemia (min)	IPC to IRI delay (min)
Richard1994[336]	rat	NR	m	none	local	NA	3	5	15	5
Rioufol1997[337]	pig	NR	NR	none	local	NA	1	10	10	10
Rivo2006[338]	rabbit	NR	NR	none	local	NA	1	5	5	10
Roesner2007[339]	pig	yes	m	none	local	NA	1	10	10	15
Roesner2010[340]	pig	yes	m	none	local	NA	1	10	10	15
Rohmann1994[341]	pig	NR	m/f	none	local	NA	1	10	10	15
Sack1993_ip30[342]	pig	NR	m	none	local	NA	2	10	20	30
Sack1993_ip4dip[342]	pig	NR	m	none	local	NA	4	10	40	30
Sack1993_ip60[342]	pig	NR	m	none	local	NA	2	10	20	60
Sack1993_ip60ip[342]	pig	NR	m	none	local	NA	4	10	40	30
Sanada2001a_exp1[343]	dog	NR	NR	none	local	NA	4	5	20	5
Sanada2001a_exp2[343]	dog	NR	NR	none	local	NA	4	5	20	5
Sanada2001b[344]	dog	NR	NR	none	local	NA	4	5	20	5
Sanada2004[345]	dog	NR	NR	none	local	NA	1	5	5	25
Sandhu1997_3cy[346]	rabbit	NR	m/f	none	local	NA	3	5	15	10
Sandhu1997_lstab[346]	rabbit	NR	m/f	none	local	NA	1	5	5	10
Sandhu1997_sstab[346]	rabbit	NR	m/f	none	local	NA	1	5	5	10
Sanz1995[347]	pig	NR	NR	none	local	NA	2	5	10	5
Sarkar2012[348]	mouse	NR	NR	none	local	NA	2	5	10	5
Sasamori2006[349]	dog	NR	m/f	none	local	NA	4	5	20	5
Sato2007_45is[350]	rat	NR	m	none	local	NA	12	2	24	1440
Sato2007_60is[350]	rat	NR	m	none	local	NA	12	2	24	1440
Sbarouni2006_healthy[351]	rabbit	NR	f	none	local	NA	2	5	10	10
Sbarouni2006_ovx[351]	rabbit	NR	f	ovariectomy	local	NA	2	5	10	10
Schmidt2014[352]	pig	NR	m/f	none	remote	hind limb	4	5	20	5
Schoemaker2000_IPC[353]	rat	NR	m	none	local	NA	1	15	15	10
Schoemaker2000_RIPC[353]	rat	NR	m	none	remote	MAO	1	15	15	10
Schott1990[354]	pig	NR	m	none	local	NA	1	10	10	30
Schultz1995[355]	rat	NR	m	none	local	NA	3	5	15	5
Schultz1996[356]	rat	NR	m	none	local	NA	3	5	15	5
Schultz1997a[357]	rat	NR	NR	none	local	NA	1	5	5	10

comparison	species	opioids pre-IPC	sex (m/f)	condition / comorbidity	site of IPC	remote organ	IPC # ischemic cycli	IPC ischemic cycle (min)	IPC total ischemia (min)	IPC to IRI delay (min)
Schultz1997b[358]	rat	NR	m	none	local	NA	3	5	15	5
Schultz1997c[359]	rat	NR	m	none	local	NA	3	5	15	5
Schultz1997d[360]	rat	NR	m	none	local	NA	3	5	15	5
Schultz1998a_1cy[361]	rat	NR	m	none	local	NA	1	5	5	20
Schultz1998a_3cy[361]	rat	NR	m	none	local	NA	3	5	15	5
Schultz1998b_exp1[362]	rat	NR	m	none	local	NA	3	5	15	5
Schultz1998b_exp2[362]	rat	NR	m	none	local	NA	3	5	15	5
Schultz1998c[363]	rat	NR	m	none	local	NA	3	5	15	5
Schulz1994[364]	pig	NR	m/f	none	local	NA	1	10	10	15
Schwartz1997[365]	dog	none	m/f	none	local	NA	1	5	5	10
Schwartz2001_2cy[366]	dog	NR	m/f	none	local	NA	2	10	20	10
Schwartz2001_ip10[366]	dog	NR	m/f	none	local	NA	1	10	10	10
Schwartz2001_ip2h[366]	dog	NR	m/f	none	local	NA	1	10	10	120
Schwartz2001_ip3h[366]	dog	NR	m/f	none	local	NA	1	10	10	180
Schwartz2001_ip5h[366]	dog	NR	m/f	none	local	NA	1	10	10	300
Schwartz2002[367]	pig	NR	m/f	none	local	NA	2	5	10	10
Schwarz1997[368]	pig	NR	m	none	local	NA	2	10	20	30
Schwarz1998_ip24h[369]	pig	NR	m	none	local	NA	2	10	20	1440
Schwarz1998_ip30[369]	pig	NR	m	none	local	NA	2	10	20	30
Schwarz1998_ip5h[369]	pig	NR	m	none	local	NA	2	10	20	300
Schwarz1998_ip60[369]	pig	NR	m	none	local	NA	2	10	20	60
Schwarz1998_ip90[369]	pig	NR	m	none	local	NA	2	10	20	90
Sebbag1996[370]	dog	yes	m/f	none	local	NA	4	5	20	5
Shahid2008[371]	rat	NR	m	none	remote	hind limb	1	15	15	10
Shattock1996[372]	pig	NR	m	none	local	NA	2	8	16	8
Shekarforoush2011[373]	rat	NR	m	none	local	NA	1	5	5	NR
Sheldrick1999[374]	rabbit	NR	m	none	local	NA	1	5	5	10
Shen2008[375]	pig	NR	f	none	local	NA	2	10	20	1440
Shen2013[376]	rat	NR	m	none	local	NA	3	7	21	10
Shinmura2000[377]	rabbit	NR	m	none	local	NA	6	4	24	1440
Shinmura2002[378]	rabbit	NR	m	none	local	NA	6	4	24	1440

comparison	species	opioids pre-IPC	sex (m/f)	condition / comorbidity	site of IPC	remote organ	IPC # ischemic cycli	IPC ischemic cycle (min)	IPC total ischemia (min)	IPC to IRI delay (min)
Shintani2004[379]	dog	NR	NR	none	local	NA	4	5	20	5
Singh2000[380]	rat	NR	m/f	none	local	NA	4	5	20	1440
Singh2004[381]	rat	NR	m/f	none	remote	RAO	4	5	20	10
Sivarajah2005[382]	rat	NR	m	none	local	NA	2	5	10	5
Sivarajah2006[383]	rat	NR	m	none	local	NA	2	5	10	5
Smits1998[384]	pig	NR	m/f	none	local	NA	2	5	10	10
Smul2011[385]	rabbit	NR	m	none	local	NA	1	5	5	30
Speechly-Dick1994a[386]	rat	NR	m	none	local	NA	1	5	5	10
Speechly-Dick1994b_healthy[387]	rat	NR	m	none	local	NA	1	5	5	10
Speechly-Dick1994b_hypert[387]	rat	NR	m	hypertension	local	NA	1	5	5	10
Strohm2000[388]	pig	NR	m	none	local	NA	2	10	20	10
Su2006[389]	rat	NR	m	none	local	NA	2	5	10	5
Suzuki1998_2ip[390]	rabbit	NR	m	none	local	NA	1	2	2	5
Suzuki1998_3ip[390]	rabbit	NR	m	none	local	NA	1	3	3	5
Takano1998a[391]	rabbit	NR	m	none	local	NA	6	4	24	1440
Takano1998b[392]	rabbit	NR	m	none	local	NA	6	4	24	1440
Takano2000a[393]	rabbit	NR	m	none	local	NA	6	4	24	1440
Takano2000b[394]	rabbit	NR	m	none	local	NA	6	4	24	1440
Takaoka1999_IPC[395]	rabbit	NR	m	none	local	NA	1	5	5	20
Takaoka1999_RIPC[395]	rabbit	NR	m	none	remote	RAO	1	10	10	20
Takasaki1998[396]	rat	NR	m	none	local	NA	3	5	15	5
Talukder2010_f30is[397]	mouse	NR	f	none	local	NA	3	5	15	5
Talukder2010_f60is[397]	mouse	NR	f	none	local	NA	3	5	15	5
Talukder2010_m30is[397]	mouse	NR	m	none	local	NA	3	5	15	5
Talukder2010_m60is[397]	mouse	NR	m	none	local	NA	3	5	15	5
Tanaka1994a_4cy24h[398]	rabbit	NR	m	none	local	NA	4	5	20	1440
Tanaka1994a_4cy48h[398]	rabbit	NR	m	none	local	NA	4	5	20	2880
Tanaka1994a_ip24h[398]	rabbit	NR	m	none	local	NA	1	5	5	1440
Tanaka1994a_ip2h[398]	rabbit	NR	m	none	local	NA	1	5	5	120
Tanaka1994a_ip5[398]	rabbit	NR	m	none	local	NA	1	5	5	5
Tanaka1994b[399]	rabbit	NR	m	none	local	NA	1	5	5	5

comparison	species	opioids pre-IPC	sex (m/f)	condition / comorbidity	site of IPC	remote organ	IPC # ischemic cycli	IPC ischemic cycle (min)	IPC total ischemia (min)	IPC to IRI delay (min)
Tang2004[400]	rabbit	NR	m	none	local	NA	6	4	24	1440
Tang2005_exp1[401]	rabbit	NR	m	none	local	NA	6	4	24	1440
Tang2005_exp2[401]	rabbit	NR	m	none	local	NA	6	4	24	1440
Tang2005_hyperchol[401]	rabbit	NR	m	hypercholesterolemia	local	NA	6	4	24	1440
Tang2006_ip2_30is[402]	rat	NR	m	none	local	NA	12	2	24	2
Tang2006_ip2_45is[402]	rat	NR	m	none	local	NA	12	2	24	2
Tang2006_ip2_60is[402]	rat	NR	m	none	local	NA	12	2	24	2
Tang2006_ip24h_30is[402]	rat	NR	m	none	local	NA	12	2	24	1440
Tang2006_ip24h_45is[402]	rat	NR	m	none	local	NA	12	2	24	1440
Tang2006_ip24h_60is[402]	rat	NR	m	none	local	NA	12	2	24	1440
Tanhehco2000[403]	rabbit	NR	m	none	local	NA	2	5	10	10
Tanno2000_1cy[404]	rat	NR	m	none	local	NA	1	5	5	5
Tanno2000_2cyexp1[404]	rat	NR	m	none	local	NA	2	5	10	5
Tanno2000_2cyexp2[404]	rat	NR	m	none	local	NA	2	5	10	5
Tanno2000_2cyexp3[404]	rat	NR	m	none	local	NA	2	5	10	5
Tanno2000_2cyexp4[404]	rat	NR	m	none	local	NA	2	5	10	5
Thornton1990[405]	rabbit	NR	m/f	none	local	NA	2	5	10	10
Thornton1992[406]	rabbit	NR	m/f	none	local	NA	2	5	10	10
Thornton1993a[407]	rabbit	NR	m/f	none	local	NA	1	5	5	10
Thornton1993b[408]	rabbit	NR	m/f	none	local	NA	1	5	5	10
Thornton1993c[409]	rabbit	NR	m/f	none	local	NA	2	5	10	10
Tissier2001[410]	rabbit	NR	m	none	local	NA	6	4	24	1440
Tissier2002_3drep[411]	rabbit	NR	m	none	local	NA	6	4	24	1440
Tissier2002_3drep_hist[411]	rabbit	NR	m	none	local	NA	6	4	24	1440
Tissier2002_3hrep[411]	rabbit	NR	m	none	local	NA	6	4	24	1440
Toller1999[412]	dog	NR	m/f	none	local	NA	1	2	2	60
Toombs1993a[413]	rabbit	NR	m/f	none	local	NA	1	5	5	10
Toufektsian2003[414]	rat	NR	m	none	local	NA	1	5	5	5
Toyoda2000_30is[415]	sheep	NR	m/f	none	local	NA	1	5	5	5
Toyoda2000_45is[415]	sheep	NR	m/f	none	local	NA	1	5	5	5
Toyoda2000_60is[415]	sheep	NR	m/f	none	local	NA	1	5	5	5

comparison	species	opioids pre-IPC	sex (m/f)	condition / comorbidity	site of IPC	remote organ	IPC # ischemic cycli	IPC ischemic cycle (min)	IPC total ischemia (min)	IPC to IRI delay (min)
Tranter2010[416]	mouse	NR	m/f	none	local	NA	6	4	24	1440
Tsovolas2008[417]	rabbit	NR	m	none	local	NA	2	5	10	10
Tsuchida1992[418]	rabbit	NR	m	none	local	NA	1	5	5	5
Tsuchida1994a[419]	rabbit	NR	m/f	none	local	NA	1	5	5	10
Tsuchida1994b[420]	rabbit	NR	m/f	none	local	NA	1	5	5	120
Tsuchida1998_20is[421]	rat	NR	m	none	local	NA	2	5	10	5
Tsuchida1998_30is[421]	rat	NR	m	none	local	NA	2	5	10	5
Tsuchida1998_40is[421]	rat	NR	m	none	local	NA	2	5	10	5
Turan2008[422]	rat	NR	m	none	local	NA	1	5	5	10
Ueda1999_healthy[423]	rabbit	NR	NR	none	local	NA	1	5	5	10
Ueda1999_hyperchol[423]	rabbit	NR	NR	hypercholesterolemia	local	NA	1	5	5	10
Uematsu1998[424]	sheep	NR	m/f	none	local	NA	1	5	5	5
Valtchanova2004_ip24h[425]	rat	NR	m	none	local	NA	3	5	15	1440
Valtchanova2004_ip5[425]	rat	NR	m	none	local	NA	3	5	15	5
Valtchanova-Matchouganska2003[426]	rat	NR	m	none	local	NA	3	5	15	5
VandenDoel1998_030is[427]	rat	NR	m	none	local	NA	1	15	15	10
VandenDoel1998_045is[427]	rat	NR	m	none	local	NA	1	15	15	10
VandenDoel1998_060is[427]	rat	NR	m	none	local	NA	1	15	15	10
VandenDoel1998_120is[427]	rat	NR	m	none	local	NA	1	15	15	10
VanderHeide1994[428]	dog	yes	m/f	none	local	NA	4	5	20	5
VanWinkle1994b[429]	pig	yes	NR	none	local	NA	2	10	20	10
VanWylen1994[430]	dog	yes	m/f	none	local	NA	2	5	10	10
Vetterlein2006[431]	rat	NR	m	none	local	NA	3	5	15	10
Vincent2012[432]	mouse	NR	m	none	local	NA	3	1	3	10
Virag2013[433]	mouse	NR	f	none	local	NA	3	5	15	5
Vladic2011_mouse[434]	mouse	NR	m	hyperglycaemia	local	NA	4	5	20	5
Vladic2011_rbhealthy[434]	rabbit	NR	m	hyperglycaemia	local	NA	1	5	5	15
Vladic2011_rbhypergly[434]	rabbit	NR	m	hyperglycaemia	local	NA	1	5	5	15
Vogt1998_ip30[435]	pig	NR	m	none	local	NA	2	10	20	30
Vogt1998_ip60[435]	pig	NR	m	none	local	NA	2	10	20	60
Vogt1998_ip60ip[435]	pig	NR	m	none	local	NA	4	10	40	30

comparison	species	opioids pre-IPC	sex (m/f)	condition / comorbidity	site of IPC	remote organ	IPC # ischemic cycli	IPC ischemic cycle (min)	IPC total ischemia (min)	IPC to IRI delay (min)
Vogt2001_1cy[436]	pig	NR	m	none	local	NA	1	10	10	30
Vogt2001_2cy[436]	pig	NR	m	none	local	NA	2	10	20	30
Wall1994[437]	rabbit	NR	m/f	none	local	NA	1	5	5	10
Walsh1994[438]	rabbit	NR	NR	none	local	NA	1	5	5	10
Wang2001[439]	rat	NR	m	none	remote	MAO	1	30	30	1440
Wang2002[440]	rat	NR	m	none	remote	MAO	1	25	25	15
Wang2009[441]	rat	NR	m	none	remote	hind limb	3	5	15	5
WangS2004[442]	sheep	NR	m	none	local	NA	3	5	15	10
WangY2004_ip24h[443]	rabbit	NR	m	none	local	NA	6	4	24	1440
WangY2004_ip72h[443]	rabbit	NR	m	none	local	NA	6	4	24	4320
Watanabe2006[444]	rat	NR	m	coronary stenosis	local	NA	2	5	10	5
Weber2005[445]	rat	NR	m	none	local	NA	3	5	15	5
Weber2008[446]	rat	NR	m	none	local	NA	1	5	5	1440
Weinbrenner2002_IPC[447]	rat	NR	m	none	local	NA	3	5	15	5
Weinbrenner2002_RIPC05[447]	rat	NR	m	none	remote	IAO	1	5	5	10
Weinbrenner2002_RIPC10[447]	rat	NR	m	none	remote	IAO	1	10	10	10
Weinbrenner2002_RIPC15[447]	rat	NR	m	none	remote	IAO	1	15	15	10
Weinbrenner2004_1cy[448]	rat	NR	m	none	local	NA	1	5	5	5
Weinbrenner2004_3cy[448]	rat	NR	m	none	local	NA	3	5	15	5
Weinbrenner2004_RIPC[448]	rat	NR	m	none	remote	IAO	1	15	15	10
Wolfe1993_ip30[449]	rat	NR	m	none	local	NA	4	5	20	30
Wolfe1993_ip360[449]	rat	NR	m	none	local	NA	4	5	20	360
Wolfe1993_ip5[449]	rat	NR	m	none	local	NA	4	5	20	5
Wolfe1993_ip60[449]	rat	NR	m	none	local	NA	4	5	20	60
Wolfrum2002[450]	rat	NR	m	none	remote	MAO	1	15	15	15
Wolfrum2005[451]	rat	NR	m	none	remote	MAO	1	15	15	15
Wong2010[452]	rat	NR	m	none	local	NA	3	5	15	5
Wong2012_IPC1[453]	rat	NR	m	none	local	NA	3	5	15	5
Wong2012_IPC2[453]	rat	NR	m	none	local	NA	3	5	15	5
Wong2012_RIPC1[453]	rat	NR	m	none	remote	hind limb	3	5	15	5
Wong2012_RIPC2[453]	rat	NR	m	none	remote	hind limb	3	5	15	5

comparison	species	opioids pre-IPC	sex (m/f)	condition / comorbidity	site of IPC	remote organ	IPC # ischemic cycli	IPC ischemic cycle (min)	IPC total ischemia (min)	IPC to IRI delay (min)
Wu2001[454]	rabbit	NR	m	none	local	NA	1	5	5	5
Wu2008a[455]	rat	NR	m	diabetes	local	NA	3	5	15	5
Wu2008b_IPC[456]	rat	NR	m	none	local	NA	3	5	15	5
Wu2008b_RIPC[456]	rat	NR	m	none	remote	hind limb	9	5	45	1440
Wu2009[457]	rat	NR	m	diabetes	local	NA	3	5	15	5
Wu2011_IPC[458]	rat	NR	m	none	local	NA	3	5	15	5
Wu2011_RIPC[458]	rat	NR	m	none	remote	hind limb	9	5	45	1440
Wu2013[459]	rabbit	NR	NR	none	local	NA	2	10	20	10
Xiang2011_early[460]	mouse	NR	m	none	local	NA	3	4	12	4
Xiang2011_late24hR[460]	mouse	NR	m	none	local	NA	3	4	12	1440
Xiang2011_late40R[460]	mouse	NR	m	none	local	NA	3	4	12	1440
Xiao2001_ip24h[461]	rat	NR	m	none	local	NA	6	4	24	1440
Xiao2001_ip48h[461]	rat	NR	m	none	local	NA	6	4	24	2880
Xiao2001_ip72h[461]	rat	NR	m	none	local	NA	6	4	24	4320
Xiong2011[462]	rat	NR	m	none	local	NA	3	5	15	5
Xu2001[463]	rabbit	NR	m/f	none	local	NA	3	5	15	20
Xu2002[464]	rabbit	NR	m/f	none	local	NA	1	5	5	10
Xu2006[465]	rabbit	NR	m/f	none	local	NA	1	5	5	10
Xu2010[466]	rat	NR	m	none	local	NA	3	5	15	1440
Xu2013[467]	rabbit	NR	m/f	none	local	NA	1	5	5	10
Xuan2007[468]	mouse	NR	NR	none	local	NA	6	4	24	4
Yada2006[469]	dog	yes	m/f	none	local	NA	4	5	20	5
Yamada2002_2ip[470]	rabbit	NR	m	none	local	NA	1	2	2	10
Yamada2002_5ip[470]	rabbit	NR	m	none	local	NA	1	5	5	10
Yamasaki1997_05ip[471]	rabbit	NR	m	none	local	NA	1	5	5	5
Yamasaki1997_10ip[471]	rabbit	NR	m	none	local	NA	1	10	10	5
Yamasaki1997_15ip[471]	rabbit	NR	m	none	local	NA	1	15	15	5
Yamasaki2000_45is[472]	rabbit	NR	m	none	local	NA	1	5	5	5
Yamasaki2000_50is[472]	rabbit	NR	m	none	local	NA	1	5	5	5
Yamasaki2000_ip005[472]	rabbit	NR	m	none	local	NA	1	5	5	5
Yamasaki2000_ip040[472]	rabbit	NR	m	none	local	NA	1	5	5	40

comparison	species	opioids pre-IPC	sex (m/f)	condition / comorbidity	site of IPC	remote organ	IPC # ischemic cycli	IPC ischemic cycle (min)	IPC total ischemia (min)	IPC to IRI delay (min)
Yamasaki2000_ip120[472]	rabbit	NR	m	none	local	NA	1	5	5	120
Yamashita1998_ip10[473]	rat	NR	m	none	local	NA	4	3	12	10
Yamashita1998_ip24h[473]	rat	NR	m	none	local	NA	4	3	12	1440
Yamashita1998_ip3h[473]	rat	NR	m	none	local	NA	4	3	12	180
Yamashita2000[474]	rat	NR	m	none	local	NA	4	3	12	1440
Yang1993_1cy[475]	rabbit	NR	m/f	none	local	NA	1	5	5	120
Yang1993_2cy[475]	rabbit	NR	m/f	none	local	NA	2	5	10	10
Yang1996[476]	rabbit	none	NR	none	local	NA	4	5	20	1440
Yang2006[477]	pig	NR	NR	none	local	NA	3	10	30	5
Yang2010[478]	macaque	NR	m	none	local	NA	2	10	20	10
Yang2011[479]	rat	NR	m	none	local	NA	4	3	12	5
YangX2013[480]	rat	NR	m	none	local	NA	3	5	15	5
YangZ2013[481]	mouse	NR	NR	none	local	NA	2	5	10	5
Yao1993a[482]	dog	NR	m/f	none	local	NA	1	10	10	10
Yao1993b[483]	dog	NR	m/f	none	local	NA	1	10	10	10
Yao1994a_ip10[484]	dog	NR	m/f	none	local	NA	1	10	10	10
Yao1994a_ip60[484]	dog	NR	m/f	none	local	NA	1	10	10	60
Yao1994b_10ip[485]	dog	NR	m/f	none	local	NA	1	10	10	10
Yao1994b_3ip[485]	dog	NR	m/f	none	local	NA	1	3	3	10
Yao1997[486]	dog	NR	m/f	none	local	NA	1	10	10	60
Yellon1992[487]	rat	NR	m	none	local	NA	1	5	5	10
Yorozuya2004_1cy[488]	rabbit	NR	m	none	local	NA	1	5	5	5
Yorozuya2004_4cy[488]	rabbit	NR	m	none	local	NA	4	5	20	5
Ytrehus1994[489]	rabbit	NR	m/f	none	local	NA	1	5	5	10
Yu2007[490]	rat	NR	m	none	local	NA	3	5	15	1440
Yu2010[491]	rat	NR	NR	none	local	NA	12	5	60	NR
Yu2011[492]	rat	NR	m	none	local	NA	2	5	10	5
Zacharowski2007[493]	rat	none	m	none	local	NA	3	2	6	10
Zhang2004[494]	rat	NR	m	none	local	NA	3	5	15	5
Zhang2010[495]	rat	NR	f	none	local	NA	2	5	10	5
ZhangS2006[496]	rat	NR	m	none	local	NA	3	5	15	5

comparison	species	opioids pre-IPC	sex (m/f)	condition / comorbidity	site of IPC	remote organ	IPC # ischemic cycli	IPC ischemic cycle (min)	IPC total ischemia (min)	IPC to IRI delay (min)
ZhangX2006[497]	rabbit	NR	m/f	none	local	NA	3	5	15	5
Zhao2003[498]	dog	yes	m/f	none	local	NA	1	5	5	10
Zhao2012_ip24h[499]	rat	NR	m	none	local	NA	3	5	15	1440
Zhao2012_ip5[499]	rat	NR	m	none	local	NA	3	5	15	5
Zhu2007[500]	mouse	NR	m	none	local	NA	3	5	15	15
Zhu2009_IPC[501]	rat	NR	NR	diabetes	local	NA	3	5	15	5
Zhu2009_RIPC[501]	rat	NR	NR	diabetes	remote	hind limb	9	5	45	5
Zhu2011_IPC[502]	rat	NR	m	diabetes	local	NA	3	5	15	5
Zhu2011_RIPC[502]	rat	NR	m	diabetes	remote	hind limb	9	5	45	5
Zhu2013[503]	rat	NR	m	none	remote	hind limb	3	5	15	5

Meta-analysis includes data from 785 independent comparisons identified from 503 studies. Multiple comparisons from a single study are indicated by an underscore followed by a comparison code. IPC = ischemic preconditioning, m = male, f = female, IRI = ischemia-reperfusion injury, NR = not reported, NA = not applicable, IAO = infrarenal aorta occlusion, RAO = renal artery occlusion, MAO = mesenteric artery occlusion, LCX = left circumflex coronary artery occlusion.

Supplemental Table B - study quality and risk of bias - page 1 of 12

study	reporting - randomization	reporting - blinding	reporting - powercalculation	bias - random group allocation	bias - groups similar at baseline	bias - blinded group allocation	bias - random housing	bias - blind intervention	bias - random outcome assessment	bias - blind outcome assessment	bias - reporting of drop-outs	bias - other
Alcindor2004[1]	Y	N	N	?	?	?	?	?	?	?	L	L
Alizadeh2011[2]	N	N	N	?	L	?	?	?	?	?	H	L
Alkhulaifi1993[3]	Y	N	N	?	?	?	?	?	?	?	?	L
Amour2009[4]	Y	N	N	?	L	?	?	?	?	?	H	H
Andreadou2004[5]	Y	N	N	?	?	?	?	?	?	?	L	L
Andreadou2006[6]	Y	N	N	?	?	?	?	?	?	?	L	L
Andreadou2011[7]	Y	N	N	?	?	?	?	?	?	?	?	L
Aouam2005[8]	Y	N	N	?	L	?	?	?	?	?	H	H
Argaud2004[9]	Y	N	N	?	L	?	?	?	?	?	?	L
Argaud2005a[10]	Y	N	N	?	L	?	?	?	?	?	?	L
Argaud2005b[11]	Y	Y	N	?	L	?	?	?	?	?	L	L
Argaud2005c[12]	N	N	N	?	L	?	?	?	?	?	L	L
Argaud2008[13]	N	N	N	?	?	?	?	?	?	?	?	L
Auchampach1992[14]	N	N	N	?	?	?	?	?	?	?	?	L
Auchampach1993[15]	Y	N	N	?	?	?	?	?	?	?	L	L
Auchampach2004[16]	Y	N	N	?	?	?	?	?	?	?	H	L
Aye1999[17]	N	N	N	?	L	?	?	?	?	?	?	L
Baines1997[18]	N	N	N	?	?	?	?	?	?	?	?	L
Baines1999[19]	N	N	N	?	?	?	?	?	?	?	?	L
Barbosa1996[20]	Y	N	N	?	L	?	?	?	?	?	?	L
Basalay2012[21]	N	N	N	?	L	?	?	?	?	?	H	L
Baumert2007[22]	Y	Y	Y	?	?	?	?	?	?	L	L	L
Baxter1994[23]	Y	N	N	?	?	?	?	?	?	?	L	L
Baxter1995[24]	N	N	N	?	?	?	?	?	?	?	L	L
Baxter1997[25]	Y	Y	N	?	?	?	?	?	?	?	?	L
Belosjorow1999[26]	N	N	N	?	?	?	?	?	?	?	L	L
Belosjorow2003[27]	N	N	N	?	?	?	?	?	?	?	L	L
Bencsik2010[28]	N	N	N	?	?	?	?	?	?	?	?	L
Bernardo1999[29]	Y	N	N	?	H	?	?	?	?	?	?	L
Bibli2013[30]	Y	N	N	?	L	?	?	?	?	?	L	L
Blokhin2008a[31]	N	N	N	?	?	?	?	?	?	?	L	L
Blokhin2008b[32]	N	N	N	?	?	?	?	?	?	?	H	L
Boengler2007[33]	N	N	N	?	H	?	?	?	?	?	H	L
Brandenburger2012[34]	Y	N	N	?	?	?	?	?	?	?	?	L
Bukhari1995[35]	Y	N	N	?	?	?	?	?	?	?	?	L
Burckhartt1995[36]	Y	N	N	?	?	?	?	?	?	?	L	L
Burns1996[37]	N	N	N	?	?	?	?	?	?	?	L	L
Cai2008[38]	N	N	N	?	L	?	?	?	?	?	?	L
Cai2013[39]	N	N	N	?	?	?	?	?	?	?	?	L
Canyon2005[40]	Y	N	N	?	?	?	?	?	?	?	H	L
Cason1997[41]	Y	N	N	L	?	?	?	?	?	?	L	L
Chazov2001[42]	N	N	N	?	?	?	?	?	?	?	?	L
Chen1997[43]	N	N	N	?	?	?	?	?	?	?	?	L
Chen2005[44]	Y	Y	N	?	?	?	?	?	?	L	L	L
Chen2008[45]	Y	N	N	?	L	?	?	?	?	?	?	L

study	reporting - randomization	reporting - blinding	reporting - powercalculation	bias - random group allocation	bias - groups similar at baseline	bias - blinded group allocation	bias - random housing	bias - blind intervention	bias - random outcome assessment	bias - blind outcome assessment	bias - reporting of drop-outs	bias - other
Cheng2009[46]	Y	N	N	?	?	?	?	?	?	?	?	L
Cheng2010a[47]	N	N	N	?	?	?	?	?	?	?	?	L
Cheng2010b[48]	N	N	N	?	?	?	?	?	?	?	?	L
Chiari2002[49]	N	N	N	?	?	?	?	?	?	?	L	L
Chien1996[50]	Y	N	N	?	L	?	?	?	?	?	L	L
Chien1999[51]	Y	N	N	?	L	?	?	?	?	?	H	L
Cohen1991[52]	Y	N	N	?	?	?	?	?	?	?	L	L
Cohen1994[53]	Y	N	N	H	?	?	?	?	?	?	L	L
Cohen1999[54]	Y	N	N	?	?	?	?	?	?	?	L	L
Cohen2000[55]	Y	Y	N	?	?	?	?	?	?	?	L	L
Colantonio2004[56]	Y	Y	N	?	?	?	?	?	?	?	?	L
Dai2003[57]	Y	N	N	?	L	?	?	?	?	?	L	L
Dai2009[58]	Y	N	N	?	L	?	?	?	?	?	L	L
Dairaku2002[59]	N	N	N	?	?	?	?	?	?	?	?	L
Daleau2001[60]	N	N	N	?	L	?	?	?	?	?	?	L
Das2006[61]	N	N	N	?	L	?	?	?	?	?	?	L
Das2007[62]	N	N	N	?	L	?	?	?	?	?	?	L
Das2012[63]	Y	Y	N	?	L	?	?	?	?	L	L	L
Dawn2002[64]	N	N	N	?	?	?	?	?	?	?	L	L
Dawn2004a[65]	N	N	N	?	?	?	?	?	?	?	L	L
Dawn2004b[66]	N	N	N	?	?	?	?	?	?	?	H	L
Demiryurek2005a[67]	N	N	N	?	L	?	?	?	?	?	L	L
Demiryurek2005b[68]	N	N	N	?	L	?	?	?	?	?	?	L
DePaulis2013[69]	Y	N	N	?	L	?	?	?	?	?	?	L
Depre2010[70]	N	N	N	?	?	?	?	?	?	?	L	L
Deuchar2007[71]	N	N	N	?	?	?	?	?	?	?	?	L
DeZeeuw2001[72]	N	N	N	?	?	?	?	?	?	?	L	L
Ding2000[73]	N	N	N	?	?	?	?	?	?	?	L	L
Ding2001[74]	N	N	N	?	L	?	?	?	?	?	?	L
Dong2004[75]	N	N	N	?	?	?	?	?	?	?	?	L
Ebel2003[76]	Y	N	N	?	L	?	?	?	?	?	?	L
Ebel2009[77]	Y	N	N	?	L	?	?	?	?	?	?	L
Eckle2006[78]	N	N	N	?	?	?	?	?	?	?	?	L
Eckle2012[79]	N	N	N	?	?	?	?	?	?	?	?	L
Erikson1996[80]	Y	N	N	?	H	?	?	?	?	?	L	L
Fang2008[81]	Y	Y	N	?	?	?	?	?	?	?	?	L
Farhat2001[82]	Y	N	N	?	?	?	?	?	?	?	?	L
Fisher2002[83]	N	N	N	?	?	?	?	?	?	?	?	L
Flack3rd1991[84]	N	N	N	?	?	?	?	?	?	?	?	L
Flaherty2008[85]	N	N	N	?	L	?	?	?	?	?	H	L
Flynn2005[86]	Y	Y	N	?	?	?	?	?	?	?	?	L
Fryer1998[87]	Y	N	N	?	L	?	?	?	?	?	?	L
Fryer1999[88]	Y	N	N	?	L	?	?	?	?	?	?	L
Fryer2000[89]	Y	N	N	?	?	?	?	?	?	?	?	L
Fryer2001a[90]	N	N	N	?	L	?	?	?	?	?	?	L

study	reporting - randomization	reporting - blinding	reporting - powercalculation	bias - random group allocation	bias - groups similar at baseline	bias - blinded group allocation	bias - random housing	bias - blind intervention	bias - random outcome assessment	bias - blind outcome assessment	bias - reporting of drop-outs	bias - other
Fryer2001b[91]	N	N	N	?	L	?	?	?	?	?	?	L
Fryer2001c[92]	N	N	N	?	L	?	?	?	?	?	?	L
Fryer2002[93]	Y	N	N	?	L	?	?	?	?	?	?	L
Fukuma2000[94]	N	N	N	?	L	?	?	?	?	?	?	L
Galagudza2007a[95]	N	N	N	?	L	?	?	?	?	?	L	L
Galagudza2007b[96]	N	N	N	?	?	?	?	?	?	?	L	L
Galagudza2009[97]	N	N	N	?	?	?	?	?	?	?	L	L
Gao2000[98]	N	Y	N	?	?	?	?	?	?	L	?	L
Gao2003[99]	Y	N	N	?	?	?	?	?	?	?	?	L
Gao2012[100]	Y	N	N	?	?	?	?	?	?	?	L	L
Ge2011[101]	Y	N	N	?	?	?	?	?	?	?	?	L
Gerczuk2012[102]	Y	N	N	?	L	?	?	?	?	?	L	L
Gho1996[103]	N	N	N	?	?	?	?	?	?	?	L	L
Gomez2011[104]	Y	N	N	?	?	?	?	?	?	?	?	L
Gomoll1996[105]	N	N	N	?	L	?	?	?	?	?	?	L
Goto1993[106]	Y	N	N	?	L	?	?	?	?	?	L	L
Gourine2005[107]	Y	N	N	?	L	?	?	?	?	?	L	L
Gozal2005[108]	Y	N	N	L	?	?	?	?	?	?	?	L
Griol-Charhbili2005[109]	N	Y	N	?	L	?	?	?	?	?	?	L
Gross1992[110]	Y	N	N	?	?	?	?	?	?	?	L	L
Gross2009[111]	N	N	N	H	?	?	?	?	?	?	L	L
Grover1992[112]	N	N	N	?	?	?	?	?	?	?	?	L
Grover1996[113]	N	N	N	?	?	?	?	?	?	?	L	L
Grund1997[114]	Y	N	N	?	?	?	?	?	?	?	L	L
Gu2008[115]	Y	N	N	?	?	?	?	?	?	?	L	L
Gumina1999[116]	Y	N	N	?	?	?	?	?	?	?	L	L
Gumina2001[117]	Y	N	N	?	?	?	?	?	?	?	L	L
Gumina2005[118]	N	N	N	?	?	?	?	?	?	?	L	L
Guo1998[119]	N	N	N	?	L	?	?	?	?	?	?	L
Guo1999[120]	N	N	N	?	?	?	?	?	?	?	?	L
Guo2001[121]	N	N	N	?	L	?	?	?	?	?	?	L
Guo2007[122]	N	N	N	?	?	?	?	?	?	?	?	L
Guo2012[123]	N	N	N	?	L	?	?	?	?	?	L	L
Gysembergh1998[124]	N	N	N	?	?	?	?	?	?	?	?	L
Haessler1994[125]	Y	N	N	?	?	?	?	?	?	?	L	L
Haessler1997[126]	Y	N	N	?	?	?	?	?	?	?	L	L
Hale1992[127]	Y	N	N	?	L	?	?	?	?	?	H	L
Hale1999[128]	Y	N	N	?	L	?	?	?	?	?	H	H
Halkos2004[129]	Y	N	N	?	?	?	?	?	?	?	?	L
Hampton2003[130]	N	Y	N	?	?	?	?	?	?	?	L	L
Haruna1998[131]	Y	N	N	?	L	?	?	?	?	?	?	L
Hatori2001[132]	Y	Y	N	L	?	?	?	?	?	?	L	L
Hausenloy2012[133]	Y	N	N	?	?	?	?	?	?	?	?	L
He2009[134]	Y	N	N	?	?	?	?	?	?	?	L	L
Heinen2011[135]	Y	N	N	?	L	?	?	?	?	?	?	L

Supplemental Table B - study quality and risk of bias - page 4 of 12

study	reporting - randomization	reporting - blinding	reporting - powercalculation	bias - random group allocation	bias - groups similar at baseline	bias - blinded group allocation	bias - random housing	bias - blind intervention	bias - random outcome assessment	bias - blind outcome assessment	bias - reporting of drop-outs	bias - other
HemetsbergerH2008[136]	N	N	N	?	?	?	?	?	?	?	?	L
HemetsbergerR2008[137]	N	N	N	?	?	?	?	?	?	?	?	L
Hiasa2001[138]	Y	N	N	?	L	?	?	?	?	?	?	L
Hibert2013[139]	N	N	N	?	?	?	?	?	?	?	?	L
Hide1996[140]	N	N	N	?	L	?	?	?	?	?	L	L
Hinokiyama2003[141]	Y	Y	N	L	?	?	?	?	?	?	L	L
Hiroi2010[142]	N	N	N	?	?	?	?	?	?	?	?	L
Hoshida1994[143]	N	N	N	?	?	?	?	?	?	?	H	L
Htun1998[144]	N	N	N	?	?	?	?	?	?	?	?	L
Hu2002[145]	Y	N	N	?	?	?	?	?	?	?	L	L
Huang1999[146]	N	N	N	?	?	?	?	?	?	?	L	L
Huang2004[147]	Y	N	N	?	?	?	?	?	?	?	L	L
Huang2011[148]	Y	N	N	?	?	?	?	?	?	?	L	L
Huang2013[149]	Y	N	N	?	?	?	?	?	?	?	L	H
Iliodromitis1994[150]	Y	N	N	?	?	?	?	?	?	?	L	L
Iliodromitis1996a[151]	N	N	N	?	?	?	?	?	?	?	?	L
Iliodromitis1996b[152]	N	N	N	?	?	?	?	?	?	?	?	L
Iliodromitis1997[153]	Y	N	N	?	L	?	?	?	?	?	L	L
Iliodromitis2002a[154]	N	N	N	?	L	?	?	?	?	?	?	L
Iliodromitis2002b[155]	N	N	N	?	L	?	?	?	?	?	L	L
Iliodromitis2003[156]	N	N	N	?	L	?	?	?	?	?	?	L
Iliodromitis2004[157]	N	N	N	?	L	?	?	?	?	?	?	L
Iliodromitis2006a[158]	N	N	N	?	L	?	?	?	?	?	?	L
Iliodromitis2006b[159]	N	N	N	?	L	?	?	?	?	?	?	L
Iliodromitis2008[160]	N	N	N	?	L	?	?	?	?	?	L	L
Iliodromitis2013[161]	Y	Y	N	?	?	?	?	?	?	L	?	L
Imagawa1997[162]	Y	N	N	?	?	?	?	?	?	?	L	L
Imagawa1998[163]	N	N	N	?	L	?	?	?	?	?	L	L
Imagawa1999[164]	Y	N	N	?	L	?	?	?	?	?	L	L
Ismaeil1999[165]	Y	N	N	L	?	?	?	?	?	?	L	L
Iwamoto1991[166]	N	N	N	?	L	?	?	?	?	?	L	L
Jaberansari2001[167]	Y	N	N	?	?	?	?	?	?	?	?	L
Jancso2004[168]	N	N	N	?	?	?	?	?	?	?	?	L
Jansco2005[169]	Y	N	N	?	?	?	?	?	?	?	L	L
Ji2013[170]	Y	Y	N	?	?	?	?	?	?	?	L	L
Jiao2002[171]	Y	N	N	?	L	?	?	?	?	?	?	L
Jung2000[172]	Y	Y	N	?	L	?	?	?	?	?	H	L
Junhua1997[173]	N	N	N	?	?	?	?	?	?	?	?	L
Kalakech2013[174]	N	Y	N	?	?	?	?	?	?	L	L	L
Kapadia1997[175]	N	N	N	?	?	?	?	?	?	?	H	H
Kara 2004[176]	N	N	N	?	L	?	?	?	?	?	?	L
Kara2006[177]	Y	N	N	?	L	?	?	?	?	?	L	L
Kariya1997[178]	Y	N	N	?	L	?	?	?	?	?	L	L
Katakam2007[179]	Y	N	N	?	?	?	?	?	?	?	?	L
Kersten1997[180]	Y	N	N	?	?	?	?	?	?	?	L	L

study	reporting - randomization	reporting - blinding	reporting - powercalculation	bias - random group allocation	bias - groups similar at baseline	bias - blinded group allocation	bias - random housing	bias - blind intervention	bias - random outcome assessment	bias - blind outcome assessment	bias - reporting of drop-outs	bias - other
Kersten1998[181]	Y	N	N	?	?	?	?	?	?	?	L	L
Kersten2000[182]	N	N	N	?	?	?	?	?	?	?	L	L
Kingma1999[183]	N	N	N	?	L	?	?	?	?	?	?	L
Kingma2011[184]	Y	N	Y	?	?	?	?	?	?	?	L	H
Kingma2013[185]	Y	N	Y	?	?	?	?	?	?	?	L	L
Kirsch2000[186]	Y	N	N	?	?	?	?	?	?	?	?	L
Kis2003[187]	N	N	N	?	L	?	?	?	?	?	?	L
Kitakaze1994a[188]	N	N	N	?	?	?	?	?	?	?	?	L
Kitakaze1994b[189]	N	Y	N	?	?	?	?	?	?	L	L	L
Kitakaze1996a[190]	N	Y	N	?	?	?	?	?	?	L	L	H
Kitakaze1996b[191]	Y	N	N	?	?	?	?	?	?	?	L	H
Kitakaze2000[192]	N	Y	N	?	?	?	?	?	?	L	L	L
Kocoglu2008[193]	Y	Y	N	L	L	?	?	?	?	L	?	L
Kohler2007[194]	N	N	N	?	?	?	?	?	?	?	?	L
Kojima1995[195]	N	N	N	?	?	?	?	?	?	?	?	H
Kolettis2013[196]	N	N	Y	?	?	?	?	?	?	?	L	L
Koneru2007[197]	Y	N	N	?	?	?	?	?	?	?	?	L
Koning1994a[198]	Y	N	N	?	?	?	?	?	?	?	?	L
Koning1994b[199]	N	N	N	?	?	?	?	?	?	?	?	L
Kositprapa2001[200]	Y	N	N	?	L	?	?	?	?	?	?	L
Kouchi1998[201]	Y	N	N	?	L	?	?	?	?	?	?	L
Kremastinos2000[202]	Y	N	N	?	L	?	?	?	?	?	L	L
Kristensen2004[203]	N	N	N	?	?	?	?	?	?	?	?	L
Kudej2006[204]	N	N	N	?	?	?	?	?	?	?	?	L
Kunuthur2012[205]	N	N	N	?	L	?	?	?	?	?	?	L
Kuzmin2000[206]	N	N	N	?	L	?	?	?	?	?	L	L
Kuzume2004[207]	Y	Y	N	?	?	?	?	?	?	L	L	L
Kuzuya1993[208]	N	N	N	?	?	?	?	?	?	?	L	L
Landim2013[209]	N	N	N	?	?	?	?	?	?	?	L	L
Lang2006[210]	Y	N	N	?	?	?	?	?	?	?	?	L
Lange2006[211]	Y	N	N	?	L	?	?	?	?	?	L	L
Lankford2006[212]	N	N	N	?	?	?	?	?	?	?	?	L
Larsen2007[213]	Y	Y	Y	L	L	?	?	?	?	L	?	H
Lasley1995[214]	N	N	N	?	?	?	?	?	?	?	?	L
Lazou2006[215]	Y	N	N	?	?	?	?	?	?	?	?	L
Lee2000[216]	N	Y	N	?	L	?	?	?	?	L	?	L
Lee2004[217]	N	N	N	?	?	?	?	?	?	?	L	L
Li_G1990[218]	Y	N	N	?	L	?	?	?	?	?	L	L
Li_Q2003[219]	N	N	N	?	L	?	?	?	?	?	L	L
Li_R2009[220]	N	N	N	H	L	H	H	H	H	H	?	L
Li_S2008[221]	N	N	N	?	?	?	?	?	?	?	?	L
Li_S2009[222]	N	N	N	?	?	?	?	?	?	?	?	L
Li_S2010[223]	Y	N	N	?	L	?	?	?	?	?	?	L
Li_W2003[224]	N	N	N	?	L	?	?	?	?	?	L	L
Li_X2012a[225]	N	N	N	?	?	?	?	?	?	?	?	L

Supplemental Table B - study quality and risk of bias - page 6 of 12

study	reporting - randomization	reporting - blinding	reporting - powercalculation	bias - random group allocation	bias - groups similar at baseline	bias - blinded group allocation	bias - random housing	bias - blind intervention	bias - random outcome assessment	bias - blind outcome assessment	bias - reporting of drop-outs	bias - other
Li_X2012b[226]	Y	Y	N	?	?	?	?	?	?	?	L	L
Li_Y1992a[227]	Y	N	N	?	?	?	?	?	?	?	?	H
Li_Y1992b[228]	Y	Y	N	?	?	?	?	H	?	?	?	H
Li_Y1993a[229]	Y	Y	N	?	L	?	?	L	?	?	?	L
Li_Y1993b[230]	Y	Y	N	?	L	?	?	L	?	?	?	L
Li_Y1994[231]	Y	N	N	?	L	?	?	?	?	?	?	L
Li_Y1995[232]	Y	N	N	?	L	?	?	?	?	?	?	L
Li_Y2011[233]	N	N	N	?	?	?	?	?	?	?	?	L
Liao2013[234]	Y	N	N	L	?	?	?	?	?	?	H	L
Liaudet2001[235]	N	N	N	?	?	?	?	?	?	?	?	L
Lie2010[236]	Y	N	N	?	L	?	?	?	?	L	?	L
Liem2001[237]	N	N	N	?	L	?	?	?	?	?	L	L
Liem2005[238]	N	N	N	?	L	?	?	?	?	?	?	L
Lim2007[239]	N	N	N	?	?	?	?	?	?	?	?	L
Lim2010[240]	Y	N	N	?	L	?	?	?	?	?	?	L
Liu1993a[241]	Y	N	N	?	L	?	?	?	?	?	?	L
Liu1993b[242]	Y	N	N	?	L	?	?	?	?	?	L	L
Liu1994[243]	N	N	N	?	?	?	?	?	?	?	L	L
Liu2005[244]	Y	N	N	?	?	?	?	?	?	?	?	L
Liu2008[245]	N	N	N	?	?	?	?	?	?	?	L	L
LiuG1992a[246]	Y	N	N	?	?	?	?	?	?	?	?	H
LiuG1992b[247]	Y	N	N	?	?	?	?	?	?	?	?	L
LiuY1992[248]	Y	N	N	?	?	?	?	?	?	?	?	L
Loke1998[249]	Y	N	N	?	?	?	?	?	?	?	?	L
Lott1996[250]	Y	N	N	?	L	?	?	?	?	?	?	L
Lotz2011[251]	Y	N	N	?	L	?	?	?	?	?	?	H
Lou2003[252]	Y	N	N	?	?	?	?	?	?	?	?	L
Lu2009[253]	N	N	N	?	?	?	?	?	?	?	?	L
Lu2011[254]	Y	N	N	?	L	?	?	?	?	?	?	L
Lu2012[255]	Y	N	N	?	L	?	?	?	?	?	L	L
Luo2004[256]	Y	N	N	?	?	?	?	?	?	?	?	L
Manintveld2005[257]	N	N	N	?	L	?	?	?	?	?	?	L
Manintveld2011[258]	N	N	N	?	?	?	?	?	?	?	?	L
Marber1993[259]	N	N	N	H	?	?	?	?	?	?	H	H
Martin1997[260]	N	N	N	?	?	?	?	?	?	?	?	L
Matsuda1993[261]	N	N	N	?	?	?	?	?	?	?	H	L
Matsumura2000[262]	N	N	N	?	L	?	?	?	?	?	L	L
McVey1999a[263]	N	N	N	?	L	?	?	?	?	?	L	L
McVey1999b[264]	N	N	N	?	?	?	?	?	?	?	?	L
Mei1998[265]	Y	N	N	?	?	?	?	?	?	?	L	L
Miki1996[266]	Y	N	N	?	L	?	?	?	?	?	L	L
Miki1998a[267]	N	N	N	?	?	?	?	?	?	?	L	L
Miki1998b[268]	N	N	N	?	L	?	?	?	?	?	L	L
Miki1999[269]	N	N	N	?	?	?	?	?	?	?	L	H
Miller1999[270]	Y	Y	N	?	?	?	?	?	?	L	?	L

Supplemental Table B - study quality and risk of bias - page 7 of 12

study	reporting - randomization	reporting - blinding	reporting - powercalculation	bias - random group allocation	bias - groups similar at baseline	bias - blinded group allocation	bias - random housing	bias - blind intervention	bias - random outcome assessment	bias - blind outcome assessment	bias - reporting of drop-outs	bias - other
Minamino1996[271]	N	N	N	?	?	?	?	?	?	?	?	L
Miura1991[272]	Y	N	N	?	L	?	?	?	?	?	L	L
Miura1992[273]	N	N	N	?	L	?	?	?	?	?	L	L
Miura1998[274]	Y	N	N	H	L	?	?	?	?	?	L	L
Mizumura1995[275]	Y	N	N	?	?	?	?	?	?	?	L	L
Mizumura1996[276]	N	N	N	?	?	?	?	?	?	?	L	L
Mizumura1997[277]	Y	N	N	?	?	?	?	?	?	?	?	L
Morgan1999[278]	Y	N	N	?	?	?	?	?	?	?	?	L
Mortensen2006[279]	N	N	N	?	?	?	?	?	?	?	?	L
Mullenheim2001a[280]	N	N	N	?	L	?	?	?	?	?	?	L
Mullenheim2001b[281]	Y	N	N	?	L	?	?	?	?	?	?	L
Mullenheim2001c[282]	Y	N	N	?	L	?	?	?	?	?	?	L
Mullenheim2003[283]	N	N	N	?	?	?	?	?	?	?	?	L
Munch-Ellingsen1997[284]	N	N	N	?	?	?	?	?	?	?	L	L
Munch-Ellingsen1998[285]	N	N	N	?	?	?	?	?	?	?	L	L
Murry1986[286]	Y	N	N	?	?	?	?	?	?	?	L	L
Murry1991[287]	N	N	N	?	?	?	?	?	?	?	H	H
Nadtochiy2009[288]	N	N	N	?	L	?	?	?	?	?	?	L
Nakae2000[289]	N	N	N	?	L	?	?	?	?	?	L	L
Nakano1997[290]	N	N	N	?	L	?	?	?	?	?	?	L
Naumenko2010[291]	Y	N	N	?	?	?	?	?	?	?	?	L
Nawada1997[292]	N	N	N	?	L	?	?	?	?	?	L	L
Neckar2002[293]	N	N	N	?	L	?	?	?	?	?	L	L
Nieszner2002[294]	N	N	N	?	?	?	?	?	?	?	?	L
Nishihara2006[295]	N	N	N	?	L	?	?	?	?	?	?	L
Nithipatikom2006a[296]	N	N	N	H	?	?	?	?	?	?	?	L
Nithipatikom2006b[297]	N	N	N	H	?	?	?	?	?	?	?	L
Node1997[298]	Y	N	N	?	?	?	?	?	?	?	L	L
Nozawa1999[299]	Y	Y	N	?	?	L	?	L	?	L	L	L
Nozawa2003[300]	N	N	N	?	L	?	?	?	?	?	?	L
Okamura1999[301]	Y	N	N	?	L	?	?	?	?	?	?	L
Okubo2000[302]	Y	N	N	?	L	?	?	?	?	?	L	L
Okubo2004a[303]	Y	N	N	?	L	?	?	?	?	?	H	L
Okubo2004b[304]	N	N	N	?	L	?	?	?	?	?	?	L
Ovize1992[305]	Y	N	N	?	?	?	?	?	?	?	L	L
Ovize1995[306]	Y	N	N	?	?	?	?	?	?	?	L	L
Pagel2007[307]	Y	N	N	?	L	?	?	?	?	?	L	L
Patel2001[308]	Y	N	N	?	L	?	?	?	?	?	?	L
Patel2002[309]	Y	N	N	?	L	?	?	?	?	?	?	L
Patel2005[310]	N	N	N	?	L	?	?	?	?	?	L	L
Peart2003[311]	Y	N	N	?	?	?	?	?	?	?	?	L
Pell1998[312]	Y	Y	N	?	L	?	?	?	?	L	?	L
Perricone2013[313]	N	Y	N	?	?	?	?	?	?	L	?	L
Petrischev2006[314]	N	N	N	?	?	?	?	?	?	?	?	L
Ping1999[315]	N	N	N	?	L	?	?	?	?	?	L	L

study	reporting - randomization	reporting - blinding	reporting - powercalculation	bias - random group allocation	bias - groups similar at baseline	bias - blinded group allocation	bias - random housing	bias - blind intervention	bias - random outcome assessment	bias - blind outcome assessment	bias - reporting of drop-outs	bias - other
Ping2002[316]	N	N	N	?	?	?	?	?	?	?	?	L
Piot1997[317]	Y	N	N	?	?	?	?	?	?	?	?	L
Poulsen2014[318]	N	N	N	?	?	?	?	?	?	?	L	L
Przyklenk1993[319]	Y	Y	N	?	?	?	?	?	?	L	?	L
Przyklenk1995[320]	Y	N	N	?	?	?	?	?	?	?	L	L
Przyklenk1996[321]	Y	Y	N	?	?	?	?	?	?	L	H	H
Przyklenk1997[322]	Y	N	N	?	?	?	?	?	?	?	L	L
Przyklenk2001[323]	Y	N	N	?	?	?	?	?	?	?	?	L
Przyklenk2003[324]	Y	Y	N	?	?	?	?	?	?	L	?	L
Qian1996[325]	Y	N	N	?	?	?	?	?	?	?	?	L
Qian1999[326]	N	N	N	?	L	?	?	?	?	?	?	L
Qiu1997a[327]	N	N	N	?	L	?	?	?	?	?	L	L
Qiu1997b[328]	N	N	Y	?	?	?	?	?	?	?	L	L
Rajesh2003[329]	Y	N	N	?	L	?	?	?	?	?	?	L
Rajesh2004[330]	N	N	N	?	L	?	?	?	?	?	?	L
Raphael2005[331]	Y	N	N	?	?	?	?	?	?	?	L	L
Redel2009[332]	Y	Y	N	?	L	?	?	?	?	L	L	L
Reffelmann2003[333]	Y	N	N	?	L	?	?	?	?	?	?	H
Ren2004[334]	N	N	N	?	?	?	?	?	?	?	L	L
Richard1993[335]	N	N	N	?	L	?	?	?	?	?	?	H
Richard1994[336]	N	N	N	?	?	?	?	?	?	?	?	L
Rioufol1997[337]	Y	N	N	L	?	?	?	?	?	?	L	L
Rivo2006[338]	Y	N	N	?	?	?	?	?	?	?	L	L
Roesner2007[339]	Y	N	N	?	L	?	?	?	?	L	L	L
Roesner2010[340]	N	Y	N	?	L	?	?	?	?	L	L	L
Rohmann1994[341]	N	N	N	?	?	?	?	?	?	?	L	L
Sack1993[342]	N	N	N	?	L	?	?	?	?	?	?	L
Sanada2001a[343]	N	N	N	?	?	?	?	?	?	?	L	L
Sanada2001b[344]	Y	Y	N	?	?	?	?	?	?	L	?	L
Sanada2004[345]	N	N	N	?	?	?	?	?	?	?	L	L
Sandhu1997[346]	N	N	N	?	?	?	?	?	?	?	?	L
Sanz1995[347]	Y	Y	N	?	?	?	?	?	L	L	L	L
Sarkar2012[348]	N	N	N	?	?	?	?	?	?	?	?	L
Sasamori2006[349]	Y	N	N	?	?	?	?	?	?	?	?	L
Sato2007[350]	N	N	N	?	L	?	?	?	?	?	L	L
Sbarouni2006[351]	N	N	N	?	L	?	?	?	?	?	H	L
Schmidt2014[352]	Y	Y	N	?	?	?	?	?	?	L	L	L
Schoemaker2000[353]	N	N	N	?	L	?	?	?	?	?	?	L
Schott1990[354]	Y	N	N	?	L	?	?	?	?	?	L	L
Schultz1995[355]	Y	N	N	?	L	?	?	?	?	?	L	L
Schultz1996[356]	Y	N	N	?	L	?	?	?	?	?	L	L
Schultz1997a[357]	Y	N	N	?	?	?	?	?	?	?	?	L
Schultz1997b[358]	Y	N	N	?	L	?	?	?	?	?	L	L
Schultz1997c[359]	Y	N	N	?	L	?	?	?	?	?	?	H
Schultz1997d[360]	Y	N	N	?	L	?	?	?	?	?	?	H

Supplemental Table B - study quality and risk of bias - page 9 of 12

study	reporting - randomization	reporting - blinding	reporting - powercalculation	bias - random group allocation	bias - groups similar at baseline	bias - blinded group allocation	bias - random housing	bias - blind intervention	bias - random outcome assessment	bias - blind outcome assessment	bias - reporting of drop-outs	bias - other
Schultz1998a[361]	N	N	N	?	L	?	?	?	?	?	L	L
Schultz1998b[362]	N	N	N	?	L	?	?	?	?	?	L	L
Schultz1998c[363]	N	N	N	?	H	?	?	?	?	?	?	H
Schulz1994[364]	Y	N	N	?	?	?	?	?	?	?	?	L
Schwartz1997[365]	N	N	N	?	?	?	?	?	?	?	L	L
Schwartz2001[366]	N	N	N	?	?	?	?	?	?	?	L	L
Schwartz2002[367]	Y	N	N	?	?	?	?	?	?	?	L	L
Schwarz1997[368]	Y	N	N	?	L	?	?	?	?	?	L	L
Schwarz1998[369]	Y	N	N	?	L	?	?	?	?	?	L	H
Sebbag1996[370]	N	N	N	?	?	?	?	?	?	?	L	L
Shahid2008[371]	Y	Y	N	?	?	?	?	?	?	?	?	L
Shattock1996[372]	N	N	N	?	?	?	?	?	?	?	?	L
Shekarforoush2011[373]	Y	N	N	?	L	?	?	?	?	?	?	L
Sheldrick1999[374]	N	N	N	?	L	?	?	?	?	?	?	L
Shen2008[375]	N	N	N	?	L	?	?	?	?	?	L	L
Shen2013[376]	Y	Y	N	?	L	?	?	?	?	L	L	L
Shinmura2000[377]	N	N	N	?	L	?	?	?	?	?	L	L
Shinmura2002[378]	N	N	N	?	L	?	?	?	?	?	L	L
Shintani2004[379]	N	Y	N	?	?	?	?	?	?	L	L	L
Singh2000[380]	N	N	N	?	?	?	?	?	?	?	?	L
Singh2004[381]	N	N	N	?	?	?	?	?	?	?	?	L
Sivarajah2005[382]	N	N	N	?	L	?	?	?	?	?	L	L
Sivarajah2006[383]	N	N	N	?	L	?	?	?	?	?	H	L
Smits1998[384]	N	N	N	?	?	?	?	?	?	?	L	L
Smul2011[385]	Y	Y	Y	?	L	?	?	?	?	L	L	L
Speechly-Dick1994a[386]	N	N	N	?	L	?	?	?	?	?	L	L
Speechly-Dick1994b[387]	N	N	N	?	L	?	?	?	?	?	?	L
Strohm2000[388]	N	N	N	?	?	?	?	?	?	?	L	L
Su2006[389]	N	N	N	?	?	?	?	?	?	?	?	L
Suzuki1998[390]	Y	N	N	?	L	?	?	?	?	?	H	L
Takano1998a[391]	N	N	N	?	L	?	?	?	?	?	L	H
Takano1998b[392]	N	N	N	?	L	?	?	?	?	?	L	H
Takano2000a[393]	N	N	N	?	L	?	?	?	?	?	L	H
Takano2000b[394]	N	N	N	?	L	?	?	?	?	?	H	L
Takaoka1999[395]	N	N	N	?	L	?	?	?	?	?	L	L
Takasaki1998[396]	N	Y	N	?	L	?	?	?	?	L	L	L
Talukder2010[397]	N	N	N	?	?	?	?	?	?	?	L	L
Tanaka1994a[398]	N	N	N	?	L	?	?	?	?	?	L	L
Tanaka1994b[399]	N	N	N	?	L	?	?	?	?	?	L	L
Tang2004[400]	N	N	N	?	L	?	?	?	?	?	L	L
Tang2005[401]	N	N	N	?	?	?	?	?	?	?	L	L
Tang2006[402]	N	N	N	?	L	?	?	?	?	?	L	L
Tanhehco2000[403]	N	N	N	?	L	?	?	?	?	?	?	L
Tanno2000[404]	Y	N	N	?	L	?	?	?	?	?	?	L
Thornton1990[405]	N	N	N	?	?	?	?	?	?	?	?	L

study	reporting - randomization	reporting - blinding	reporting - powercalculation	bias - random group allocation	bias - groups similar at baseline	bias - blinded group allocation	bias - random housing	bias - blind intervention	bias - random outcome assessment	bias - blind outcome assessment	bias - reporting of drop-outs	bias - other
Thornton1992[406]	N	N	N	H	?	H	?	?	?	?	L	L
Thornton1993a[407]	N	N	N	H	?	?	?	?	?	?	?	L
Thornton1993b[408]	Y	N	N	?	?	?	?	?	?	?	?	L
Thornton1993c[409]	N	N	N	?	?	?	?	?	?	?	L	L
Tissier2001[410]	Y	N	N	?	L	?	?	?	?	?	L	L
Tissier2002[411]	Y	N	N	?	L	?	?	?	?	?	?	L
Toller1999[412]	Y	N	N	?	?	?	?	?	?	?	L	L
Toombs1993a[413]	N	Y	N	?	?	?	?	?	?	L	?	L
Toufektsian2003[414]	Y	N	N	?	L	?	?	?	?	?	?	L
Toyoda2000[415]	Y	N	N	?	?	?	?	?	?	?	L	L
Tranter2010[416]	N	N	Y	?	?	?	?	?	?	?	?	L
Tsovolas2008[417]	Y	N	N	?	?	?	?	?	?	?	?	L
Tsuchida1992[418]	N	N	N	?	L	?	?	?	?	?	L	L
Tsuchida1994a[419]	Y	N	N	?	?	?	?	?	?	?	L	L
Tsuchida1994b[420]	Y	N	N	?	?	?	?	?	?	?	L	L
Tsuchida1998[421]	Y	N	N	?	L	?	?	?	?	?	L	L
Turan2008[422]	N	N	N	?	?	?	?	?	?	?	?	L
Ueda1999[423]	Y	N	N	?	?	?	?	?	?	?	?	L
Uematsu1998[424]	Y	N	N	?	?	?	?	?	?	?	L	L
Valtchanova2004[425]	N	N	N	?	?	?	?	?	?	?	?	L
Valtchanova-Matchouganska2003[426]	Y	N	N	?	?	?	?	?	?	?	?	L
VandenDoel1998[427]	N	N	N	?	L	?	?	?	?	?	L	L
VanderHeide1994[428]	N	N	N	?	?	?	?	?	?	?	L	L
VanWinkle1994b[429]	N	N	N	?	?	?	?	?	?	?	L	L
VanWylen1994[430]	N	N	N	?	?	?	?	?	?	?	L	L
Vetterlein2006[431]	N	N	N	?	L	?	?	?	?	?	?	L
Vincent2012[432]	Y	N	N	?	?	?	?	?	?	?	?	L
Virag2013[433]	N	N	N	?	?	?	?	?	?	?	?	L
Vladic2011[434]	Y	N	N	?	L	?	?	?	?	?	?	L
Vogt1998[435]	N	N	N	?	L	?	?	?	?	?	L	L
Vogt2001[436]	N	Y	N	?	?	?	?	?	?	?	?	L
Wall1994[437]	Y	N	N	?	?	?	?	?	?	?	?	H
Walsh1994[438]	N	N	N	?	?	?	?	?	?	H	?	L
Wang2001[439]	Y	N	N	?	L	?	?	?	?	?	L	L
Wang2002[440]	Y	N	N	?	L	?	?	?	?	?	L	L
Wang2009[441]	Y	N	N	?	?	?	?	?	?	?	?	L
WangS2004[442]	Y	N	N	?	L	?	?	?	?	?	L	L
WangY2004[443]	N	N	N	?	L	?	?	?	?	?	?	H
Watanabe2006[444]	N	N	N	?	L	?	?	?	?	?	L	L
Weber2005[445]	N	N	N	?	?	?	?	?	?	?	?	H
Weber2008[446]	Y	N	Y	L	?	?	?	?	?	?	?	L
Weinbrenner2002[447]	N	N	N	?	?	?	?	?	?	?	?	L
Weinbrenner2004[448]	N	N	N	?	H	?	?	?	?	?	?	L
Wolfe1993[449]	N	N	N	?	?	?	?	?	?	?	L	L
Wolfrum2002[450]	Y	N	N	?	L	?	?	?	?	?	?	L

study	reporting - randomization	reporting - blinding	reporting - powercalculation	bias - random group allocation	bias - groups similar at baseline	bias - blinded group allocation	bias - random housing	bias - blind intervention	bias - random outcome assessment	bias - blind outcome assessment	bias - reporting of drop-outs	bias - other
Wolfrum2005[451]	Y	N	N	?	?	?	?	?	?	?	?	L
Wong2010[452]	N	N	Y	?	L	?	?	?	?	?	L	L
Wong2012[453]	N	N	Y	?	L	?	?	?	?	?	?	L
Wu2001[454]	Y	N	N	?	L	?	?	?	?	?	L	L
Wu2008a[455]	Y	N	N	?	L	?	?	?	?	?	?	L
Wu2008b[456]	Y	N	N	?	L	?	?	?	?	?	?	L
Wu2009[457]	Y	N	N	?	L	?	?	?	?	?	?	L
Wu2011[458]	Y	N	N	?	L	?	?	?	?	?	?	L
Wu2013[459]	Y	N	N	?	?	?	?	?	?	?	L	H
Xiang2011[460]	Y	Y	N	?	?	?	?	?	?	L	?	L
Xiao2001[461]	Y	N	N	?	?	?	?	?	?	?	?	L
Xiong2011[462]	Y	Y	Y	?	?	?	?	?	?	L	L	L
Xu2001[463]	Y	N	N	?	?	?	?	?	?	?	L	L
Xu2002[464]	N	Y	N	?	?	?	?	?	?	L	?	L
Xu2006[465]	Y	N	N	?	?	?	?	?	?	?	L	L
Xu2010[466]	Y	N	N	?	?	?	?	?	?	?	L	L
Xu2013[467]	Y	N	N	?	?	?	?	?	?	?	L	L
Xuan2007[468]	N	N	N	?	?	?	?	?	?	?	?	L
Yada2006[469]	N	N	N	?	?	?	?	?	?	?	?	L
Yamada2002[470]	N	N	N	?	L	?	?	?	?	?	L	L
Yamasaki1997[471]	Y	N	N	?	L	?	?	?	?	?	L	L
Yamasaki2000[472]	N	N	N	?	L	?	?	?	?	?	L	L
Yamashita1998[473]	N	N	N	?	L	?	?	?	?	?	?	L
Yamashita2000[474]	N	N	N	?	L	?	?	?	?	?	L	L
Yang1993[475]	N	N	N	?	?	?	?	?	?	?	L	L
Yang1996[476]	Y	N	N	H	?	?	?	?	?	?	L	L
Yang2006[477]	Y	N	N	?	?	?	?	?	?	?	L	L
Yang2010[478]	N	N	N	?	L	?	?	?	?	?	L	L
Yang2011[479]	Y	N	N	?	?	?	?	?	?	?	L	L
YangX2013[480]	N	Y	N	?	L	?	?	?	?	L	?	L
YangZ2013[481]	N	N	N	?	?	?	?	?	?	?	?	L
Yao1993a[482]	Y	N	N	?	?	?	?	?	?	?	L	L
Yao1993b[483]	Y	N	N	?	?	?	?	?	?	?	L	L
Yao1994a[484]	Y	N	N	?	?	?	?	?	?	?	L	L
Yao1994b[485]	N	N	N	?	?	?	?	?	?	?	L	L
Yao1997[486]	Y	N	N	?	?	?	?	?	?	?	L	L
Yellon1992[487]	N	N	N	H	L	?	?	?	?	?	L	L
Yorozuya2004[488]	Y	Y	N	?	L	?	?	?	?	L	L	L
Ytrehus1994[489]	N	N	N	?	?	?	?	?	?	?	?	L
Yu2007[490]	Y	N	N	?	?	?	?	?	?	?	?	H
Yu2010[491]	Y	N	N	?	?	?	?	?	?	?	?	L
Yu2011[492]	N	N	N	?	L	?	?	?	?	?	H	L
Zacharowski2007[493]	N	Y	N	?	L	?	?	?	?	L	L	L
Zhang2004[494]	Y	N	N	?	?	?	?	?	?	?	L	H
Zhang2010[495]	N	N	N	?	?	?	?	?	?	?	?	L

study	reporting - randomization	reporting - blinding	reporting - powercalculation	bias - random group allocation	bias - groups similar at baseline	bias - blinded group allocation	bias - random housing	bias - blind intervention	bias - random outcome assessment	bias - blind outcome assessment	bias - reporting of drop-outs	bias - other
ZhangS2006[496]	N	N	N	?	?	?	?	?	?	?	?	L
ZhangX2006[497]	Y	N	N	?	?	?	?	?	?	?	L	L
Zhao2003[498]	Y	N	N	?	?	?	?	?	?	?	?	H
Zhao2012[499]	N	N	N	?	?	?	?	?	?	?	?	L
Zhu2007[500]	N	Y	N	?	?	?	?	?	?	L	?	L
Zhu2009[501]	Y	N	N	?	L	?	?	?	?	?	?	L
Zhu2011[502]	Y	N	N	?	L	?	?	?	?	?	?	H
Zhu2013[503]	Y	Y	N	?	L	?	?	?	?	L	?	L

Meta-analysis includes data from 785 independent comparisons identified from 503 studies. Y = yes, N = not reported, ? = unclear risk of bias, L = low risk of bias, H = high risk of bias.

Wever et al. 2015 - Determinants of the efficacy of cardiac ischemic preconditioning: a systematic review and meta-analysis of animal studies

Supplemental table C - Meta-analysis study quality

	# pub	# comp	(%)	MD	[95%CI]
**all** (T^2 164.4, I^2 94.7%)	503	785	(100)	24.6	[23.5, 25.6]
**reported randomization**					
P>0.11, adj. R^2 = 0.42%					
not reported	258	420	(54)	25.3	[24.0, 26.7]
yes	245	365	(47)	23.7	[21.7, 25.7]
rodents					
P>0.98, adj. R^2 = -0.33%					
not reported	198	200	(58)	26.8	[24.9, 28.7]
yes	185	144	(42)	26.8	[24.9, 28.7]
non-rodents					
P>0.10, adj. R^2 = 0.80%					
not reported	60	220	(50)	23.9	[22.0, 25.8]
yes	60	221	(50)	21.7	[19.8, 23.6]
**reported blinding**					
P>0.69, adj. R^2 = -0.15%					
not reported	447	707	(90)	24.5	[23.5, 25.5]
yes	56	78	(10)	25.2	[21.9, 28.5]
rodents					
P>0.43, adj. R^2 = -0.03%					
not reported	345	315	(92)	27.0	[25.4, 28.5]
yes	38	29	(8)	24.9	[23.3, 26.4]
non-rodents					
P>0.17, adj. R^2 = 0.21%					
not reported	102	392	(89)	22.4	[21.0, 23.8]
yes	18	49	(11)	25.3	[23.9, 26.7]
**random group allocation (selection bias)**					
P>0.92, adj. R^2 = -0.28%					
high	11	14	(2)	23.2	[16.0, 30.3]
low	10	11	(1)	25.0	[13.9, 36.0]
unclear	482	760	(97)	24.6	[17.4, 31.8]
**groups similar at baseline (selection bias)**					
P>0.01, adj. R^2 = 1.24%					
high	5	8	(1)	27.1	[17.4, 36.8]
low	207	344	(44)	26.2	[16.4, 36.0]
unclear	291	433	(55)	23.2	[13.5, 33.0]
**blinded group allocation (selection bias)**					
not analyzed					
high	2	2	(0.3)	30.5	[11.6, 49.4]
low	1	1	(0.1)	16.2	[-18.1, 50.5]
unclear	500	782	(99)	24.6	[5.6, 43.5]

Supplemental table C (continued) - Meta-analysis study quality

	# pub	# comp	(%)	MD	[95%CI]
**random housing (performance bias)**					
not analyzed					
high	1	1	(0.1)	31.0	[4.8, 57.2]
low	0	0	(0)	NA	[NA]
unclear	502	784	(99)	24.6	[-1.6, 50.8]
**blind intervention (performance bias)**					
not analyzed					
high	2	2	(0.3)	30.4	[10.6, 50.3]
low	3	3	(0.4)	27.1	[0.8, 53.3]
unclear	498	780	(99)	24.5	[4.7, 44.4]
**random outcome assessment (detection bias)**					
not analyzed					
high	1	1	(0.1)	31.0	[4.8, 57.2]
low	1	1	(0.1)	17.1	[-21.3, 55.5]
unclear	501	783	(99)	24.6	[-1.7, 50.8]
**blind outcome assessment (detection bias)**					
$P>0.87$, adj. $R^2 = -0.28\%$					
high	2	2	(0.3)	29.5	[10.9, 48.1]
low	40	52	(7)	24.6	[5.6, 43.6]
unclear	461	731	(93)	24.5	[6.0, 43.1]
**reporting of drop-outs (attrition bias)**					
$P>0.01$, adj. $R^2 = 1.29\%$					
high	27	42	(5)	24.4	[20.3, 28.5]
low	226	340	(43)	22.9	[18.5, 27.3]
unclear	250	403	(51)	26.0	[21.7, 30.4]

Total # comparisons = 8, corrected P<0.006; pub = publications, comp = comparisons, NA = not applicable, adj. = adjusted, MD = weighted difference in means, RoB = risk of bias, ass. = assessment.

1. Alcindor D, Krolikowski JG, Pagel PS, Warltier DC, Kersten JR. Cyclooxygenase-2 mediates ischemic, anesthetic, and pharmacologic preconditioning in vivo. *Anesthesiology*. 2004;100:547-554
2. Alizadeh AM, Faghihi M, Sadeghipour HR, Mohammadghasemi F, Khori V. Role of endogenous oxytocin in cardiac ischemic preconditioning. *Regul Pept*. 2011;167:86-90
3. Alkhulaifi AM, Pugsley WB, Yellon DM. The influence of the time period between preconditioning ischemia and prolonged ischemia on myocardial protection. *Cardioscience*. 1993;4:163-169
4. Amour J, Brzezinska AK, Weihrauch D, Billstrom AR, Zielonka J, Krolikowski JG, Bienengraeber MW, Warltier DC, Pratt PF, Jr., Kersten JR. Role of heat shock protein 90 and endothelial nitric oxide synthase during early anesthetic and ischemic preconditioning. *Anesthesiology*. 2009;110:317-325
5. Andreadou I, Iliodromitis EK, Mikros E, Bofilis E, Zoga A, Constantinou M, Tsantili-Kakoulidou A, Kremastinos DT. Melatonin does not prevent the protection of ischemic preconditioning in vivo despite its antioxidant effect against oxidative stress. *Free Radic Biol Med*. 2004;37:500-510
6. Andreadou I, Iliodromitis EK, Tsovolas K, Aggeli IK, Zoga A, Gaitanaki C, Paraskevaidis IA, Markantonis SL, Beis I, Kremastinos DT. Acute administration of vitamin e triggers preconditioning via k(atp) channels and cyclic-gmp without inhibiting lipid peroxidation. *Free Radic Biol Med*. 2006;41:1092-1099
7. Andreadou I, Iliodromitis EK, Souridis V, Prokovas E, Kostidis S, Zoga A, Dagres N, Tsantili-Kakoulidou A, Kremastinos DT, Mikros E, Anastasiou-Nana M. Investigating the effect of antioxidant treatment on the protective effect of preconditioning in anesthetized rabbits. *Journal of cardiovascular pharmacology*. 2011;58:609-616
8. Aouam K, Tissier R, Bruneval P, Mandet C, Berdeaux A, Ghaleh B. Preconditioning of salvaged myocardium in conscious rabbits with postinfarction dysfunction. *American journal of physiology. Heart and circulatory physiology*. 2005;288:21
9. Argaud L, Gateau-Roesch O, Chalabreysse L, Gomez L, Loufouat J, Thivolet-Bejui F, Robert D, Ovize M. Preconditioning delays ca2+-induced mitochondrial permeability transition. *Cardiovascular research*. 2004;61:115-122
10. Argaud L, Gateau-Roesch O, Muntean D, Chalabreysse L, Loufouat J, Robert D, Ovize M. Specific inhibition of the mitochondrial permeability transition prevents lethal reperfusion injury. *Journal of molecular and cellular cardiology*. 2005;38:367-374
11. Argaud L, Gateau-Roesch O, Raisky O, Loufouat J, Robert D, Ovize M. Postconditioning inhibits mitochondrial permeability transition. *Circulation*. 2005;111:194-197
12. Argaud L, Gomez L, Gateau-Roesch O, Couture-Lepetit E, Loufouat J, Robert D, Ovize M. Trimetazidine inhibits mitochondrial permeability transition pore opening and prevents lethal ischemia-reperfusion injury. *Journal of molecular and cellular cardiology*. 2005;39:893-899
13. Argaud L, Gateau-Roesch O, Augeul L, Couture-Lepetit E, Loufouat J, Gomez L, Robert D, Ovize M. Increased mitochondrial calcium coexists with decreased reperfusion injury in postconditioned (but not preconditioned) hearts. *American journal of physiology. Heart and circulatory physiology*. 2008;294:19
14. Auchampach JA, Grover GJ, Gross GJ. Blockade of ischaemic preconditioning in dogs by the novel atp dependent potassium channel antagonist sodium 5-hydroxydecanoate. *Cardiovascular research*. 1992;26:1054-1062
15. Auchampach JA, Gross GJ. Adenosine a1 receptors, katp channels, and ischemic preconditioning in dogs. *The American journal of physiology*. 1993;264:H1327-1336
16. Auchampach JA, Jin X, Moore J, Wan TC, Kreckler LM, Ge ZD, Narayanan J, Whalley E, Kiesman W, Ticho B, Smits G, Gross GJ. Comparison of three different a1 adenosine receptor antagonists on infarct size and multiple cycle ischemic preconditioning in anesthetized dogs. *J Pharmacol Exp Ther*. 2004;308:846-856
17. Aye NN, Komori S, Hashimoto K. Effects and interaction, of cariporide and preconditioning on cardiac arrhythmias and infarction in rat in vivo. *British journal of pharmacology*. 1999;127:1048-1055
18. Baines CP, Goto M, Downey JM. Oxygen radicals released during ischemic preconditioning contribute to cardioprotection in the rabbit myocardium. *Journal of molecular and cellular cardiology*. 1997;29:207-216
19. Baines CP, Liu GS, Birincioglu M, Critz SD, Cohen MV, Downey JM. Ischemic preconditioning depends on interaction between mitochondrial k(atp) channels and actin cytoskeleton. *American Journal of Physiology - Heart and Circulatory Physiology*. 1999;276:H1361-H1368
20. Barbosa V, Sievers RE, Zaugg CE, Wolfe CL. Preconditioning ischemia time determines the degree of glycogen depletion and infarct size reduction in rat hearts. *Am Heart J*. 1996;131:224-230
21. Basalay M, Barsukevich V, Mastitskaya S, Mrochek A, Pernow J, Sjoquist PO, Ackland GL, Gourine AV, Gourine A. Remote ischaemic pre- and delayed postconditioning - similar degree of cardioprotection but distinct mechanisms. *Exp Physiol*. 2012;16:16
22. Baumert JH, Hein M, Gerets C, Baltus T, Hecker KE, Rossaint R. The effect of xenon anesthesia on the size of experimental myocardial infarction. *Anesth Analg*. 2007;105:1200-1206

23. Baxter GF, Marber MS, Patel VC, Yellon DM. Adenosine receptor involvement in a delayed phase of myocardial protection 24 hours after ischemic preconditioning. *Circulation*. 1994;90:2993-3000
24. Baxter GF, Goma FM, Yellon DM. Involvement of protein kinase c in the delayed cytoprotection following sublethal ischaemia in rabbit myocardium. *British journal of pharmacology*. 1995;115:222-224
25. Baxter GF, Goma FM, Yellon DM. Characterisation of the infarct-limiting effect of delayed preconditioning: Timecourse and dose-dependency studies in rabbit myocardium. *Basic research in cardiology*. 1997;92:159-167
26. Belosjorow S, Schulz R, Borge H, Schade FU, Heusch G. Endotoxin and ischemic preconditioning: Tnf-alpha concentration and myocardial infarct development in rabbits. *American Journal of Physiology - Heart and Circulatory Physiology*. 1999;277:H2470-H2475
27. Belosjorow S, Bolle I, Duschin A, Heusch G, Schulz R. Tnf-alpha antibodies are as effective as ischemic preconditioning in reducing infarct size in rabbits. *American journal of physiology. Heart and circulatory physiology*. 2003;284:5
28. Bencsik P, Kupai K, Giricz Z, Gorbe A, Pipis J, Murlasits Z, Kocsis GF, Varga-Orvos Z, Puskas LG, Csonka C, Csont T, Ferdinandy P. Role of inos and peroxynitrite-matrix metalloproteinase-2 signaling in myocardial late preconditioning in rats. *American journal of physiology. Heart and circulatory physiology*. 2010;299:11
29. Bernardo NL, D'Angelo M, Okubo S, Joy A, Kukreja RC. Delayed ischemic preconditioning is mediated by opening of atp-sensitive potassium channels in the rabbit heart. *The American journal of physiology*. 1999;276:H1323-1330
30. Bibli SI, Andreadou I, Lazaris E, Zoga A, Varnavas V, Andreou CC, Dagres N, Iliodromitis EK, Kyriakides ZS. Myocardial protection provided by chronic skeletal muscle ischemia is not further enhanced by ischemic pre- or postconditioning: Comparative effects on intracellular signaling. *Journal of cardiovascular pharmacology and therapeutics*. 2014;19:220-227
31. Blokhin IO, Galagudza MM, Vlasov TD, Nifontov EM, Petrishchev NN. [the relationship between ischemic preconditioning-induced infarction size limitation and duration of test myocardial ischemia]. *Ross Fiziol Zh Im I M Sechenova*. 2008;94:785-789
32. Blokhin IO, Vlasov TD, Galagudza MM, Nifontov EM, Petrishchev NN. [role of sodium-calcium exchanger in the myocardial protection against ischemia-reperfusion injury]. *Ross Fiziol Zh Im I M Sechenova*. 2008;94:284-292
33. Boengler K, Konietzka I, Buechert A, Heinen Y, Garcia-Dorado D, Heusch G, Schulz R. Loss of ischemic preconditioning's cardioprotection in aged mouse hearts is associated with reduced gap junctional and mitochondrial levels of connexin 43. *American journal of physiology. Heart and circulatory physiology*. 2007;292:1
34. Brandenburger T, Galas A, Huhn R, Pannen B, Bauer I, Heinen A. Remote ischemic preconditioning influences the protein expression of connexin 43 in the rat heart in vivo. *Applied Cardiopulmonary Pathophysiology*. 2012;16:328-329
35. Bukhari EA, Krukenkamp IB, Burns PG, Gaudette GR, Schulman JJ, al-Fagih MR, Levitsky S. Does aprotinin increase the myocardial damage in the setting of ischemia and preconditioning? *Ann Thorac Surg*. 1995;60:307-310
36. Burckhartt B, Yang XM, Tsuchida A, Mullane KM, Downey JM, Cohen MV. Acadesine extends the window of protection afforded by ischaemic preconditioning in conscious rabbits. *Cardiovascular research*. 1995;29:653-657
37. Burns PG, Krunkenkamp IB, Calderone CA, Kirvaitis RJ, Gaudette GR, Levitsky S. Is the preconditioning response conserved in senescent myocardium? *Ann Thorac Surg*. 1996;61:925-929
38. Cai ZP, Shen Z, Van Kaer L, Becker LC. Ischemic preconditioning-induced cardioprotection is lost in mice with immunoproteasome subunit low molecular mass polypeptide-2 deficiency. *Faseb J*. 2008;22:4248-4257
39. Cai Z, Luo W, Zhan H, Semenza GL. Hypoxia-inducible factor 1 is required for remote ischemic preconditioning of the heart. *Proc Natl Acad Sci U S A*. 2013;110:17462-17467
40. Canyon SJ, Dobson GP. Pretreatment with an adenosine a1 receptor agonist and lidocaine: A possible alternative to myocardial ischemic preconditioning. *J Thorac Cardiovasc Surg*. 2005;130:371-377
41. Cason BA, Gamperl AK, Slocum RE, Hickey RF. Anesthetic-induced preconditioning: Previous administration of isoflurane decreases myocardial infarct size in rabbits. *Anesthesiology*. 1997;87:1182-1190
42. Chazov EI, Kalenikova EI, Gorodetskaya EA. In vivo monitoring of hydroxyl radical generation in the rat myocardium in ischemia and reperfusion: The effect of ischemic preconditioning. *Dokl Biol Sci*. 2001;380:441-444
43. Chen K, Zhang J, Ye C. [metallothionein involvement in the delayed protection after ischemic or anoxic preconditioning in myocardium or cultured cardiomyocytes]. *Zhonghua Yi Xue Za Zhi*. 1997;77:106-110

44. Chen XG, Wu BY, Wang JK, Bai T. [mechanism of the protective effects of noninvasive limbs preconditioning on myocardial ischemia-reperfusion injury]. *Chin Med J*. 2005;118:1723-1727
45. Chen CH, Liu K, Chan JY. Anesthetic preconditioning confers acute cardioprotection via up-regulation of manganese superoxide dismutase and preservation of mitochondrial respiratory enzyme activity. *Shock*. 2008;29:300-308
46. Cheng XQ, Zhang Y, Lu Y, Li R, Weng LJ, Chen ZW. The role of beta-endorphin in protective effect of intracerebroventricular morphine preconditioning against myocardial post-ischemic injury in rats. [chinese]. *Chinese Pharmacological Bulletin*. 2009;25:1152+1153-1156
47. Cheng Y, Tan N, Yang J, Liu X, Cao X, He P, Dong X, Qin S, Zhang C. A translational study of circulating cell-free microrna-1 in acute myocardial infarction. *Clin Sci*. 2010;119:87-95
48. Cheng Y, Zhu P, Yang J, Liu X, Dong S, Wang X, Chun B, Zhuang J, Zhang C. Ischaemic preconditioning-regulated mir-21 protects heart against ischaemia/reperfusion injury via anti-apoptosis through its target pdcd4. *Cardiovascular research*. 2010;87:431-439
49. Chiari P, Piriou V, Hadour G, Rodriguez C, Loufouat J, Lehot JJ, Ovize M, Ferrera R. Preservation of ischemia and isoflurane-induced preconditioning after brain death in rabbit hearts. *American journal of physiology. Heart and circulatory physiology*. 2002;283:H1769-1774
50. Chien GL, Van Winkle DM. Naloxone blockade of myocardial ischemic preconditioning is stereoselective. *Journal of molecular and cellular cardiology*. 1996;28:1895-1900
51. Chien GL, Mohtadi K, Wolff RA, Van Winkle DM. Naloxone blockade of myocardial ischemic preconditioning does not require central nervous system participation. *Basic research in cardiology*. 1999;94:136-143
52. Cohen MV, Liu GS, Downey JM. Preconditioning causes improved wall motion as well as smaller infarcts after transient coronary occlusion in rabbits. *Circulation*. 1991;84:341-349
53. Cohen MV, Yang XM, Downey JM. Conscious rabbits become tolerant to multiple episodes of ischemic preconditioning. *Circulation research*. 1994;74:998-1004
54. Cohen MV, Yang XM, Downey JM. Smaller infarct after preconditioning does not predict extent of early functional improvement of reperfused heart. *The American journal of physiology*. 1999;277:H1754-1761
55. Cohen MV, Yang XM, Neumann T, Heusch G, Downey JM. Favorable remodeling enhances recovery of regional myocardial function in the weeks after infarction in ischemically preconditioned hearts. *Circulation*. 2000;102:579-583
56. Colantonio DA, Van Eyk JE, Przyklenk K. Stunned peri-infarct canine myocardium is characterized by degradation of troponin t, not troponin i. *Cardiovascular research*. 2004;63:217-225
57. Dai W, Kloner RA. Effects of acetaminophen on myocardial infarct size in rats. *Journal of cardiovascular pharmacology and therapeutics*. 2003;8:277-284
58. Dai W, Simkhovich BZ, Kloner RA. Ischemic preconditioning maintains cardioprotection in aging normotensive and spontaneously hypertensive rats. *Experimental gerontology*. 2009;44:344-349
59. Dairaku Y, Miura T, Harada N, Kimura M, Okamura T, Iwamoto H, Kametani R, Yamada M, Ikeda Y, Iwatate M, Kawamura S, Matsuzaki M. Effect of ischemic preconditioning and mitochondrial katp channel openers on chronic left ventricular remodeling in the ischemic-reperfused rat heart. *Circ J*. 2002;66:411-415
60. Daleau P, Boudriau S, Michaud M, Jolicoeur C, Kingma JG, Jr. Preconditioning in the absence or presence of sustained ischemia modulates myocardial cx43 protein levels and gap junction distribution. *Can J Physiol Pharmacol*. 2001;79:371-378
61. Das B, Sarkar C. Similarities between ischemic preconditioning and 17beta-estradiol mediated cardiomyocyte katp channel activation leading to cardioprotective and antiarrhythmic effects during ischemia/reperfusion in the intact rabbit heart. *Journal of cardiovascular pharmacology*. 2006;47:277-286
62. Das B, Sarkar C. Pharmacological preconditioning by levosimendan is mediated by inducible nitric oxide synthase and mitochondrial katp channel activation in the in vivo anesthetized rabbit heart model. *Vascul Pharmacol*. 2007;47:248-256
63. Das B, Sarkar C. Is preconditioning by oxytocin administration mediated by inos and/or mitochondrial k(atp) channel activation in the in vivo anesthetized rabbit heart? *Life Sci*. 2012;90:763-769
64. Dawn B, Takano H, Tang XL, Kodani E, Banerjee S, Rezazadeh A, Qiu Y, Bolli R. Role of src protein tyrosine kinases in late preconditioning against myocardial infarction. *American journal of physiology. Heart and circulatory physiology*. 2002;283:H549-556
65. Dawn B, Guo Y, Rezazadeh A, Wang OL, Stein AB, Hunt G, Varma J, Xuan YT, Wu WJ, Tan W, Zhu X, Bolli R. Tumor necrosis factor-alpha does not modulate ischemia/reperfusion injury in naive myocardium but is essential for the development of late preconditioning. *Journal of molecular and cellular cardiology*. 2004;37:51-61

66. Dawn B, Xuan YT, Guo Y, Rezazadeh A, Stein AB, Hunt G, Wu WJ, Tan W, Bolli R. Il-6 plays an obligatory role in late preconditioning via jak-stat signaling and upregulation of inos and cox-2. *Cardiovascular research*. 2004;64:61-71
67. Demiryurek S, Kara AF, Celik A, Babul A, Tarakcioglu M, Demiryurek AT. Effects of fasudil, a rho-kinase inhibitor, on myocardial preconditioning in anesthetized rats. *European journal of pharmacology*. 2005;527:129-140
68. Demiryurek S, Kara AF, Celik A, Tarakcioglu M, Bagci C, Demiryurek AT. Effects of y-27632, a selective rho-kinase inhibitor, on myocardial preconditioning in anesthetized rats. *Biochem Pharmacol*. 2005;69:49-58
69. De Paulis D, Chiari P, Teixeira G, Couture-Lepetit E, Abrial M, Argaud L, Gharib A, Ovize M. Cyclosporine a at reperfusion fails to reduce infarct size in the in vivo rat heart. *Basic research in cardiology*. 2013;108:379
70. Depre C, Park JY, Shen YT, Zhao X, Qiu H, Yan L, Tian B, Vatner SF, Vatner DE. Molecular mechanisms mediating preconditioning following chronic ischemia differ from those in classical second window. *American journal of physiology. Heart and circulatory physiology*. 2010;299:25
71. Deuchar GA, Opie LH, Lecour S. Tnfalpha is required to confer protection in an in vivo model of classical ischaemic preconditioning. *Life Sci*. 2007;80:1686-1691
72. de Zeeuw S, Lameris TW, Duncker DJ, Hasan D, Boomsma F, van den Meiracker AH, Verdouw PD. Cardioprotection in pigs by exogenous norepinephrine but not by cerebral ischemia-induced release of endogenous norepinephrine. *Stroke; a journal of cerebral circulation*. 2001;32:767-774
73. Ding YF, Zhang MM, He RR. [ischemic preconditioning reduces cardiomyocytic apoptosis in rabbit heart in vivo]. *Sheng Li Xue Bao*. 2000;52:220-224
74. Ding YF, Zhang MM, He RR. Role of renal nerve in cardioprotection provided by renal ischemic preconditioning in anesthetized rabbits. *Sheng Li Xue Bao*. 2001;53:7-12
75. Dong JH, Liu YX, Ji ES, He RR. [limb ischemic preconditioning reduces infarct size following myocardial ischemia-reperfusion in rats]. *Sheng Li Xue Bao*. 2004;56:41-46
76. Ebel D, Mullenheim J, Frassdorf J, Heinen A, Huhn R, Bohlen T, Ferrari J, Sudkamp H, Preckel B, Schlack W, Thamer V. Effect of acute hyperglycaemia and diabetes mellitus with and without short-term insulin treatment on myocardial ischaemic late preconditioning in the rabbit heart in vivo. *Pflugers Arch*. 2003;446:175-182
77. Ebel D, Toma O, Appler S, Baumann K, Frassdorf J, Preckel B, Rosen P, Schlack W, Weber NC. Ischemic preconditioning phosphorylates mitogen-activated kinases and heat shock protein 27 in the diabetic rat heart. *Horm Metab Res*. 2009;41:10-15
78. Eckle T, Grenz A, Kohler D, Redel A, Falk M, Rolauffs B, Osswald H, Kehl F, Eltzschig HK. Systematic evaluation of a novel model for cardiac ischemic preconditioning in mice. *American journal of physiology. Heart and circulatory physiology*. 2006;291:9
79. Eckle T, Hartmann K, Bonney S, Reithel S, Mittelbronn M, Walker LA, Lowes BD, Han J, Borchers CH, Buttrick PM, Kominsky DJ, Colgan SP, Eltzschig HK. Adora2b-elicited per2 stabilization promotes a hif-dependent metabolic switch crucial for myocardial adaptation to ischemia. *Nature medicine*. 2012;18:774-782
80. Erikson JM, Velasco CE. Endothelin-1 and myocardial preconditioning. *Am Heart J*. 1996;132:84-90
81. Fang J, Wu L, Chen L. Postconditioning attenuates cardiocyte ultrastructure injury and apoptosis by blocking mitochondrial permeability transition in rats. *Acta Cardiol*. 2008;63:377-387
82. Farhat F, Loisance D, Garnier JP, Kirsch M. Norepinephrine release after acute brain death abolishes the cardioprotective effects of ischemic preconditioning in rabbit. *Eur J Cardiothorac Surg*. 2001;19:313-320
83. Fisher SG, Marber MS. An in vivo model of ischaemia-reperfusion injury and ischaemic preconditioning in the mouse heart. *J Pharmacol Toxicol Methods*. 2002;48:161-169
84. Flack 3rd JE, Kimura Y, Engelman RM, Rousou JA, Iyengar J, Jones R, Das DK. Preconditioning the heart by repeated stunning improves myocardial salvage. *Circulation*. 1991;84:III369-374
85. Flaherty MP, Guo Y, Tiwari S, Rezazadeh A, Hunt G, Sanganalmath SK, Tang XL, Bolli R, Dawn B. The role of tnf-alpha receptors p55 and p75 in acute myocardial ischemia/reperfusion injury and late preconditioning. *Journal of molecular and cellular cardiology*. 2008;45:735-741
86. Flynn DM, Smith AH, Treadway JL, Levy CB, Soeller WC, Boettner WA, Wisniecki P, Plowchalk DR, Gernhardt SS, Tracey WR, Knight DR. The sulfonylurea glipizide does not inhibit ischemic preconditioning in anesthetized rabbits. *Cardiovasc Drugs Ther*. 2005;19:337-346
87. Fryer RM, Schultz JE, Hsu AK, Gross GJ. Pretreatment with tyrosine kinase inhibitors partially attenuates ischemic preconditioning in rat hearts. *The American journal of physiology*. 1998;275:H2009-2015
88. Fryer RM, Schultz JEJ, Hsu AK, Gross GJ. Importance of pkc and tyrosine kinase in single or multiple cycles of preconditioning in rat hearts. *American Journal of Physiology - Heart and Circulatory Physiology*. 1999;276:H1229-H1235

89. Fryer RM, Eells JT, Hsu AK, Henry MM, Gross GJ. Ischemic preconditioning in rats: Role of mitochondrial k(atp) channel in preservation of mitochondrial function. *American journal of physiology. Heart and circulatory physiology*. 2000;278:H305-312
90. Fryer RM, Hsu AK, Gross GJ. Mitochondrial k(atp) channel opening is important during index ischemia and following myocardial reperfusion in ischemic preconditioned rat hearts. *Journal of molecular and cellular cardiology*. 2001;33:831-834
91. Fryer RM, Patel HH, Hsu AK, Gross GJ. Stress-activated protein kinase phosphorylation during cardioprotection in the ischemic myocardium. *American journal of physiology. Heart and circulatory physiology*. 2001;281:H1184-1192
92. Fryer RM, Pratt PF, Hsu AK, Gross GJ. Differential activation of extracellular signal regulated kinase isoforms in preconditioning and opioid-induced cardioprotection. *J Pharmacol Exp Ther*. 2001;296:642-649
93. Fryer RM, Hsu AK, Wang Y, Henry M, Eells J, Gross GJ. Pkc- inhibition does not block preconditioning-induced preservation in mitochondrial atp synthesis and infarct size reduction in rats. *Basic research in cardiology*. 2002;97:47-54
94. Fukuma T, Miura T, Suzuki K, Tsuchida A, Nozawa Y, Shimamoto K. Relationship between free radicals and adenosine in the mechanism of preconditioning: Are they interrelated or independent triggers? *Mol Cell Biochem*. 2000;211:51-59
95. Galagudza MM, Nekrasova MK, Syrenskii AV, Nifontov EM. Resistance of the myocardium to ischemia and the efficacy of ischemic preconditioning in experimental diabetes mellitus. *Neurosci Behav Physiol*. 2007;37:489-493
96. Galagudza MM. [the influence of local and remote myocardial preconditioning on the incidence and severity of experimentally induced ischemic tachyarrhythmias]. *Vestn Ross Akad Med Nauk*. 2007;4:12-17
97. Galagudza MM, Syrenskii AV, Vlasov TD, Morozova LI, Egorova EI, Polumiskov VI. Effects of individual and combined administration of ethylmethylhydroxypyridine succinate and ischemic preconditioning on ischemic-reperfusive myocardial injury in rats. [russian]. *Eksperimental'naia i klinicheskaia farmakologiia*. 2009;72:22-26
98. Gao XM, Wang BH, Woodcock E, Du XJ. Expression of active alpha(1b)-adrenergic receptors in the heart does not alleviate ischemia reperfusion injury. *Journal of molecular and cellular cardiology*. 2000;32:1679-1686
99. Gao X, A., Zhang B, Shang H, Wang Y, Guo L, Zhang M, Shi H, Liu M. Protective effect of fufangdanshenfang pretreatment on myocardial ischemical reperfusion injury. [chinese]. *Chinese Journal of Clinical Rehabilitation*. 2003;7:1754-1756
100. Gao J, Kang Y, Lou J. The optimal strategy of noninvasive limb ischemic preconditioning for protecting heart against ischemia-reperfusion injury in rats. *J Surg Res*. 2012;8:8
101. Ge ZD, Ionova IA, Vladic N, Pravdic D, Hirata N, Vasquez-Vivar J, Pratt PF, Jr., Warltier DC, Pieper GM, Kersten JR. Cardiac-specific overexpression of gtp cyclohydrolase 1 restores ischaemic preconditioning during hyperglycaemia. *Cardiovascular research*. 2011;91:340-349
102. Gerczuk PZ, Breckenridge DG, Liles JT, Budas GR, Shryock JC, Belardinelli L, Kloner RA, Dai W. An apoptosis signal-regulating kinase 1 inhibitor reduces cardiomyocyte apoptosis and infarct size in a rat ischemia-reperfusion model. *Journal of cardiovascular pharmacology*. 2012;60:276-282
103. Gho BCG, Schoemaker RG, Van den Doel MA, Duncker DJ, Verdouw PD. Myocardial protection by brief ischemia in noncardiac tissue. *Circulation*. 1996;94:2193-2200
104. Gomez L, Paillard M, Price M, Chen Q, Teixeira G, Spiegel S, Lesnefsky EJ. A novel role for mitochondrial sphingosine-1-phosphate produced by sphingosine kinase-2 in ptp-mediated cell survival during cardioprotection. *Basic research in cardiology*. 2011;106:1341-1353
105. Gomoll AW. Cardioprotection associated with preconditioning in the anesthetized ferret. *Basic research in cardiology*. 1996;91:433-443
106. Goto M, Miura T, Itoya M, Sakamoto J, Iimura O. Reduction of regional contractile function by preconditioning ischemia does not play a permissive role in the infarct size-limitation by the preconditioning. *Basic research in cardiology*. 1993;88:594-606
107. Gourine AV, Molosh AI, Poputnikov D, Bulhak A, Sjoquist PO, Pernow J. Endothelin-1 exerts a preconditioning-like cardioprotective effect against ischaemia/reperfusion injury via the et(a) receptor and the mitochondrial k(atp) channel in the rat in vivo. *British journal of pharmacology*. 2005;144:331-337
108. Gozal Y, Raphael J, Rivo J, Berenshtein E, Chevion M, Drenger B. Isoflurane does not mimic ischaemic preconditioning in decreasing hydroxyl radical production in the rabbit. *Br J Anaesth*. 2005;95:442-447
109. Griol-Charhbili V, Messadi-Laribi E, Bascands JL, Heudes D, Meneton P, Giudicelli JF, Alhenc-Gelas F, Richer C. Role of tissue kallikrein in the cardioprotective effects of ischemic and pharmacological preconditioning in myocardial ischemia. *Faseb J*. 2005;19:1172-1174

110. Gross GJ, Auchampach JA. Blockade of atp-sensitive potassium channels prevents myocardial preconditioning in dogs. *Circulation research*. 1992;70:223-233
111. Gross GJ, Gauthier KM, Moore J, Campbell WB, Falck JR, Nithipatikom K. Evidence for role of epoxyeicosatrienoic acids in mediating ischemic preconditioning and postconditioning in dog. *American journal of physiology. Heart and circulatory physiology*. 2009;297:15
112. Grover GJ, Sleph PG, Dzwonczyk S. Role of myocardial atp-sensitive potassium channels in mediating preconditioning in the dog heart and their possible interaction with adenosine a1-receptors. *Circulation*. 1992;86:1310-1316
113. Grover GJ, D'Alonzo AJ, Dzwonczyk S, Parham CS, Darbenzio RB. Preconditioning is not abolished by the delayed rectifier k+ blocker dofetilide. *The American journal of physiology*. 1996;271:H1207-1214
114. Grund F, Sommerschild HT, Kirkeboen KA, Ilebekk A. Preconditioning with ischaemia reduces both myocardial oxygen consumption and infarct size in a graded pattern. *Journal of molecular and cellular cardiology*. 1997;29:3067-3079
115. Gu W, Kehl F, Krolikowski JG, Pagel PS, Warltier DC, Kersten JR. Simvastatin restores ischemic preconditioning in the presence of hyperglycemia through a nitric oxide-mediated mechanism. *Anesthesiology*. 2008;108:634-642
116. Gumina RJ, Buerger E, Eickmeier C, Moore J, Daemmgen J, Gross GJ. Inhibition of the na(+)/h(+) exchanger confers greater cardioprotection against 90 minutes of myocardial ischemia than ischemic preconditioning in dogs. *Circulation*. 1999;100:2519-2526
117. Gumina RJ, Moore J, Schelling P, Beier N, Gross GJ. Na(+)/h(+) exchange inhibition prevents endothelial dysfunction after i/r injury. *American journal of physiology. Heart and circulatory physiology*. 2001;281:H1260-1266
118. Gumina RJ, El Schultz J, Moore J, Beier N, Schelling P, Gross GJ. Cardioprotective-mimetics reduce myocardial infarct size in animals resistant to ischemic preconditioning. *Cardiovasc Drugs Ther*. 2005;19:315-322
119. Guo Y, Wu WJ, Qiu Y, Tang XL, Yang Z, Bolli R. Demonstration of an early and a late phase of ischemic preconditioning in mice. *The American journal of physiology*. 1998;275:H1375-1387
120. Guo Y, Jones WK, Xuan YT, Tang XL, Bao W, Wu WJ, Han H, Laubach VE, Ping P, Yang Z, Qiu Y, Bolli R. The late phase of ischemic preconditioning is abrogated by targeted disruption of the inducible no synthase gene. *Proc Natl Acad Sci U S A*. 1999;96:11507-11512
121. Guo Y, Bolli R, Bao W, Wu WJ, Black RG, Jr., Murphree SS, Salvatore CA, Jacobson MA, Auchampach JA. Targeted deletion of the a3 adenosine receptor confers resistance to myocardial ischemic injury and does not prevent early preconditioning. *Journal of molecular and cellular cardiology*. 2001;33:825-830
122. Guo D, Nguyen T, Ogbi M, Tawfik H, Ma G, Yu Q, Caldwell RW, Johnson JA. Protein kinase c-epsilon coimmunoprecipitates with cytochrome oxidase subunit iv and is associated with improved cytochrome-c oxidase activity and cardioprotection. *American journal of physiology. Heart and circulatory physiology*. 2007;293:27
123. Guo Y, Tukaye DN, Wu WJ, Zhu X, Book M, Tan W, Jones SP, Rokosh G, Narumiya S, Li Q, Bolli R. The cox-2/pgi2 receptor axis plays an obligatory role in mediating the cardioprotection conferred by the late phase of ischemic preconditioning. *PloS one*. 2012;7:e41178
124. Gysembergh A, Margonari H, Loufoua J, Ovize A, Andre-Fouet X, Minaire Y, Ovize M. Stretch-induced protection shares a common mechanism with ischemic preconditioning in rabbit heart. *The American journal of physiology*. 1998;274:H955-964
125. Haessler R, Kuzume K, Chien GL, Wolff RA, Davis RF, Van Winkle DM. Anaesthetics alter the magnitude of infarct limitation by ischaemic preconditioning. *Cardiovascular research*. 1994;28:1574-1580
126. Haessler R, Wolff RA, Chien GL, Davis RF, Van Winkle DM. High spinal anesthesia does not alter experimental myocardial lnfarction size or ischemic preconditioning. *Journal of Cardiothoracic and Vascular Anesthesia*. 1997;11:72-79
127. Hale SL, Kloner RA. Effect of ischemic preconditioning on regional myocardial flow in the rabbit heart. *Coronary Artery Disease*. 1992;3:133-140
128. Hale SL, Kloner RA. Ischemic preconditioning and myocardial hypothermia in rabbits with prolonged coronary artery occlusion. *The American journal of physiology*. 1999;276:H2029-2034
129. Halkos ME, Kerendi F, Corvera JS, Wang NP, Kin H, Payne CS, Sun HY, Guyton RA, Vinten-Johansen J, Zhao ZQ. Myocardial protection with postconditioning is not enhanced by ischemic preconditioning. *Ann Thorac Surg*. 2004;78:961-969
130. Hampton CR, Shimamoto A, Rothnie CL, Griscavage-Ennis J, Chong A, Dix DJ, Verrier ED, Pohlman TH. Hsp70.1 and -70.3 are required for late-phase protection induced by ischemic preconditioning of mouse hearts. *American journal of physiology. Heart and circulatory physiology*. 2003;285:24

131. Haruna T, Horie M, Kouchi I, Nawada R, Tsuchiya K, Akao M, Otani H, Murakami T, Sasayama S. Coordinate interaction between atp-sensitive k+ channel and na+, k+-atpase modulates ischemic preconditioning. *Circulation*. 1998;98:2905-2910
132. Hatori N, Segawa D, Hinokiyama K, Kimura T, Iizuka Y, Ochi M, Tanaka S. Effects of ischemic preconditioning and synchronized coronary venous retroperfusion in an off-pump coronary artery bypass grafting model. *Artif Organs*. 2001;25:47-52
133. Hausenloy DJ, Iliodromitis EK, Andreadou I, Papalois A, Gritsopoulos G, Anastasiou-Nana M, Kremastinos DT, Yellon DM. Investigating the signal transduction pathways underlying remote ischemic conditioning in the porcine heart. *Cardiovasc Drugs Ther*. 2012;30:30
134. He Y, Zeng ZY, Zhong GQ, Li JY, Li WK, Li W. Cx43 in mitochondria participates in the protection for heptanol preconditioning on myocardial ischemia/reperfusion injury of rabbits. [chinese]. *Chinese Pharmacological Bulletin*. 2009;25:1660-1665
135. Heinen NM, Putz VE, Gorgens JI, Huhn R, Gruber Y, Barthuber C, Preckel B, Pannen BH, Bauer I. Cardioprotection by remote ischemic preconditioning exhibits a signaling pattern different from local ischemic preconditioning. *Shock*. 2011;36:45-53
136. Hemetsberger H, Steiner S, Farhan S, Kvakan H, Hemetsberger R, Pavo N, Kopp C, Garamvolgyi R, Petrasi Z, Petnehazy O, Manczur F, Huber K, Wojta J, Glogar D, Gyongyosi M. Ischemic preconditioning leads to reduction of circulating mesenchymal stem cells in a porcine ischaemia/reperfusion model. *Journal fur Kardiologie*. 2008;15:348-352
137. Hemetsberger R, Posa A, Pavo N, Farhan S, Csonka C, Csont T, Ferdinandy P, Garamvolgyi R, Petrasi Z, Petnehazy O, Varga C, Pavo Jr I, Laszlo Jr F, Huber K, Wojta J, Glogar D, Gyongyosi M. Involvement of nitric oxide in the cardioprotective effect of early ischemic preconditioning in the reperfusion phase in pigs. *Journal fur Kardiologie*. 2008;15:119-122
138. Hiasa G, Hamada M, Ikeda S, Hiwada K. Ischemic preconditioning and lipopolysaccharide attenuate nuclear factor-kappab activation and gene expression of inflammatory cytokines in the ischemia-reperfused rat heart. *Jpn Circ J*. 2001;65:984-990
139. Hibert P, Prunier-Mirebeau D, Beseme O, Chwastyniak M, Tamareille S, Lamon D, Furber A, Pinet F, Prunier F. Apolipoprotein a-i is a potential mediator of remote ischemic preconditioning. *PloS one*. 2013;8:e77211
140. Hide EJ, Thiemermann C. Limitation of myocardial infarct size in the rabbit by ischaemic preconditioning is abolished by sodium 5-hydroxydecanoate. *Cardiovascular research*. 1996;31:941-946
141. Hinokiyama K, Hatori N, Ochi M, Maehara T, Tanaka S. Myocardial protective effect of lidocaine during experimental off-pump coronary artery bypass grafting. *Ann Thorac Cardiovasc Surg*. 2003;9:36-42
142. Hiroi T, Wajima T, Kaneko Y, Kiuchi Y, Shimizu S. An important role of increase in tetrahydrobiopterin via h2o2-jak2 signalling pathway in late phase of ischaemic preconditioning. *Exp Physiol*. 2010;95:609-621
143. Hoshida S, Kuzuya T, Nishida M, Yamashita N, Oe H, Hori M, Kamada T, Tada M. Adenosine blockade during reperfusion reverses the infarct limiting effect in preconditioned canine hearts. *Cardiovascular research*. 1994;28:1083-1088
144. Htun P, Ito WD, Hoefer IE, Schaper J, Schaper W. Intramyocardial infusion of fgf-1 mimics ischemic preconditioning in pig myocardium. *Journal of molecular and cellular cardiology*. 1998;30:867-877
145. Hu CP, Peng J, Xiao L, Ye F, Deng HW, Li YJ. Effect of age on alpha-calcitonin gene-related peptide-mediated delayed cardioprotection induced by intestinal preconditioning in rats. *Regul Pept*. 2002;107:137-143
146. Huang CH, Kim SJ, Ghaleh B, Kudej RK, Shen YT, Bishop SP, Vatner SF. An adenosine agonist and preconditioning shift the distribution of myocardial blood flow in conscious pigs. *The American journal of physiology*. 1999;276:H368-375
147. Huang CH, Wang JS, Chiang SC, Wang YY, Lai ST, Weng ZC. Brief pressure overload of the left ventricle preconditions rabbit myocardium against infarction. *Ann Thorac Surg*. 2004;78:628-633
148. Huang CH, Tsai SK, Chiang SC, Wang YY, Chih CL, Weng ZC, Lai ST. Brief pressure overload preconditions rabbit myocardium independent of adenosine receptor activation. *Ann Thorac Surg*. 2011;92:1727-1732
149. Huang CH, Tsai SK, Chiang SC, Lai CC, Weng ZC. The role of adenosine in preconditioning by brief pressure overload in rats. *Journal of the Formosan Medical Association = Taiwan yi zhi*. 2013
150. Iliodromitis EK, Kremastinos Th D, Bouris I, Papadopoulos C, Paraskevaidis IA, Yellon DM. The re-introduction of ischemic preconditioning is able to protect myocardium after repeated long reperfusion intervals. *Cardioscience*. 1994;5:277-281
151. Iliodromitis EK, Papadopoulos CC, Markianos M, Paraskevaidis IA, Kyriakides ZS, Kremastinos DT. Alterations in circulating cyclic guanosine monophosphate (c-gmp) during short and long ischemia in preconditioning. *Basic research in cardiology*. 1996;91:234-239
152. Iliodromitis EK, Papadopoulos C, Paraskevaidis IA, Kyriakides ZS, Flessa C, Kremastinos DT. Protection from preconditioning can be reinstated at various reperfusion intervals. *Cardiovasc Drugs Ther*. 1996;10:341-346

153. Iliodromitis EK, Kremastinos DT, Katritsis DG, Papadopoulos CC, Hearse DJ. Multiple cycles of preconditioning cause loss of protection in open-chest rabbits. *Journal of molecular and cellular cardiology*. 1997;29:915-920
154. Iliodromitis EK, Gaitanaki C, Lazou A, Bofilis E, Karavolias GK, Beis I, Kremastinos DT. Dissociation of stress-activated protein kinase (p38-mapk and jnks) phosphorylation from the protective effect of preconditioning in vivo. *Journal of molecular and cellular cardiology*. 2002;34:1019-1028
155. Iliodromitis EK, Vlahakos DV, Bofilis E, Karavolias GK, Tasouli A, Kremastinos DT. Angiotensin ii fails to induce preconditioning in vivo--the protective role of myocardial stretch due to pressure overload. *Can J Cardiol*. 2002;18:34-42
156. Iliodromitis EK, Cokkinos P, Zoga A, Steliou I, Vrettou AR, Kremastinos DT. Oral nicorandil recaptures the waned protection from preconditioning in vivo. *British journal of pharmacology*. 2003;138:1101-1106
157. Iliodromitis EK, Tasouli A, Andreadou I, Bofilis E, Zoga A, Cokkinos P, Kremastinos DT. Intravenous atenolol and esmolol maintain the protective effect of ischemic preconditioning in vivo. *European journal of pharmacology*. 2004;499:163-169
158. Iliodromitis EK, Gaitanaki C, Lazou A, Aggeli IK, Gizas V, Bofilis E, Zoga A, Beis I, Kremastinos DT. Differential activation of mitogen-activated protein kinases in ischemic and nitroglycerin-induced preconditioning. *Basic research in cardiology*. 2006;101:327-335
159. Iliodromitis EK, Zoga A, Vrettou A, Andreadou I, Paraskevaidis IA, Kaklamanis L, Kremastinos DT. The effectiveness of postconditioning and preconditioning on infarct size in hypercholesterolemic and normal anesthetized rabbits. *Atherosclerosis*. 2006;188:356-362
160. Iliodromitis EK, Aggeli IK, Gaitanaki C, Tsiafoutis I, Zoga A, Beis I, Kremastinos DT. P38-mapk is involved in restoration of the lost protection of preconditioning by nicorandil in vivo. *European journal of pharmacology*. 2008;579:289-297
161. Iliodromitis K, Farmakis D, Andreadou I, Zoga A, Bibli SI, Manolaki T, Dagres N, Iliodromitis EK, Anastasiou-Nana M, Kremastinos DT. Various models of cardiac conditioning in single or sequential periods of ischemia: Comparative effects on infarct size and intracellular signaling. *Int J Cardiol*. 2013;168:1336-1341
162. Imagawa J, Baxter GF, Yellon DM. Genistein, a tyrosine kinase inhibitor, blocks the "second window of protection" 48 h after ischemic preconditioning in the rabbit. *Journal of molecular and cellular cardiology*. 1997;29:1885-1893
163. Imagawa J, Baxter GF, Yellon DM. Myocardial protection afforded by nicorandil and ischaemic preconditioning in a rabbit infarct model in vivo. *Journal of cardiovascular pharmacology*. 1998;31:74-79
164. Imagawa J, Yellon DM, Baxter GF. Pharmacological evidence that inducible nitric oxide synthase is a mediator of delayed preconditioning. *British journal of pharmacology*. 1999;126:701-708
165. Ismaeil MS, Tkachenko I, Gamperl AK, Hickey RF, Cason BA. Mechanisms of isoflurane-induced myocardial preconditioning in rabbits. *Anesthesiology*. 1999;90:812-821
166. Iwamoto T, Miura T, Adachi T, Noto T, Ogawa T, Tsuchida A, Iimura O. Myocardial infarct size-limiting effect of ischemic preconditioning was not attenuated by oxygen free-radical scavengers in the rabbit. *Circulation*. 1991;83:1015-1022
167. Jaberansari MT, Baxter GF, Muller CA, Latouf SE, Roth E, Opie LH, Yellon DM. Angiotensin-converting enzyme inhibition enhances a subthreshold stimulus to elicit delayed preconditioning in pig myocardium. *J Am Coll Cardiol*. 2001;37:1996-2001
168. Jancso G, Gasz B, Szanto Z, Cserepes B, Roth E. Bradykinin and angiotensin-converting enzyme inhibition in cardioprotection. *Experimental and Clinical Cardiology*. 2004;9:21-25
169. Jancso G, Cserepes B, Gasz B, Benko L, Ferencz A, Borsiczky B, Lantos J, Dureja A, Kiss K, Szeberenyi J, Roth E. Effect of acetylsalicylic acid on nuclear factor-kappab activation and on late preconditioning against infarction in the myocardium. *Journal of cardiovascular pharmacology*. 2005;46:295-301
170. Ji L, Zhang X, Liu W, Huang Q, Yang W, Fu F, Ma H, Su H, Wang H, Wang J, Zhang H, Gao F. Ampk-regulated and akt-dependent enhancement of glucose uptake is essential in ischemic preconditioning-alleviated reperfusion injury. *PloS one*. 2013;8:e69910
171. Jiao XY, Luo N, Zhi JM, Zhao RR. Effects of exogenous platelet activating factor and its antagonist ginkgolide b on ischemia-reperfusion injury of myocardium in rats. *Chinese Journal of Pharmacology and Toxicology*. 2002;16:81-87
172. Jung O, Jung W, Malinski T, Wiemer G, Schoelkens BA, Linz W. Ischemic preconditioning and infarct mass: The effect of hypercholesterolemia and endothelial dysfunction. *Clin Exp Hypertens*. 2000;22:165-179
173. Junhua Z, Kui C, Jian C. Mitogen-activated protein kinase involvement in the delayed protection after ischemic or anoxic preconditioning in myocardium or cultured cardiomyocytes. [chinese]. *Chinese Journal of Cardiology*. 1997;25:215-218

174. Kalakech H, Tamareille S, Pons S, Godin-Ribuot D, Carmeliet P, Furber A, Martin V, Berdeaux A, Ghaleh B, Prunier F. Role of hypoxia inducible factor-1alpha in remote limb ischemic preconditioning. *Journal of molecular and cellular cardiology*. 2013;65:98-104
175. Kapadia SJ, Terlato JS, Most AS. Presence of a critical coronary artery stenosis does not abolish the protective effect of ischemic preconditioning. *Circulation*. 1997;95:1286-1292
176. Kara AF, Demiryurek S, Celik A, Tarakcioglu M, Demiryurek AT. Effects of trimetazidine on myocardial preconditioning in anesthetized rats. *European journal of pharmacology*. 2004;503:135-145
177. Kara AF, Demiryurek S, Celik A, Tarakcioglu M, Demiryurek AT. Effects of chronic trimetazidine treatment on myocardial preconditioning in anesthetized rats. *Fundam Clin Pharmacol*. 2006;20:449-459
178. Kariya T, Minatoguchi S, Ohno T, Yamashita K, Uno Y, Arai M, Koshiji M, Fujiwara T, Fujiwara H. Infarct size-reducing effect of ischemic preconditioning is related to alpha1b-adrenoceptors but not to alpha1a-adrenoceptors in rabbits. *Journal of cardiovascular pharmacology*. 1997;30:437-445
179. Katakam PV, Jordan JE, Snipes JA, Tulbert CD, Miller AW, Busija DW. Myocardial preconditioning against ischemia-reperfusion injury is abolished in zucker obese rats with insulin resistance. *Am J Physiol Regul Integr Comp Physiol*. 2007;292:28
180. Kersten JR, Schmeling TJ, Pagel PS, Gross GJ, Warltier DC. Isoflurane mimics ischemic preconditioning via activation of k(atp) channels: Reduction of myocardial infarct size with an acute memory phase. *Anesthesiology*. 1997;87:361-370
181. Kersten JR, Schmeling TJ, Orth KG, Pagel PS, Warltier DC. Acute hyperglycemia abolishes ischemic preconditioning in vivo. *The American journal of physiology*. 1998;275:H721-725
182. Kersten JR, Toller WG, Gross ER, Pagel PS, Warltier DC. Diabetes abolishes ischemic preconditioning: Role of glucose, insulin, and osmolality. *American journal of physiology. Heart and circulatory physiology*. 2000;278:H1218-1224
183. Kingma JG, Jr. Cardiac adaptation to ischemia-reperfusion injury. *Ann N Y Acad Sci*. 1999;874:83-99
184. Kingma Jr JG, Simard D, Voisine P, Rouleau JR. Role of the autonomic nervous system in cardioprotection by remote preconditioning in isoflurane-anaesthetized dogs. *Cardiovascular research*. 2011;89:384-391
185. Kingma JG, Simard D, Voisine P, Rouleau JR. Influence of cardiac decentralization on cardioprotection. *PloS one*. 2013;8:e79190
186. Kirsch M, Farhat F, Garnier JP, Loisance D. Acute brain death abolishes the cardioprotective effects of ischemic preconditioning in the rabbit. *Transplantation*. 2000;69:2013-2019
187. Kis A, Yellon DM, Baxter GF. Second window of protection following myocardial preconditioning: An essential role for pi3 kinase and p70s6 kinase. *Journal of molecular and cellular cardiology*. 2003;35:1063-1071
188. Kitakaze M, Hori M, Morioka T, Minamino T, Takashima S, Sato H, Shinozaki Y, Chujo M, Mori H, Inoue M, et al. Infarct size-limiting effect of ischemic preconditioning is blunted by inhibition of 5'-nucleotidase activity and attenuation of adenosine release. *Circulation*. 1994;89:1237-1246
189. Kitakaze M, Hori M, Morioka T, Minamino T, Takashima S, Sato H, Shinozaki Y, Chujo M, Mori H, Inoue M, Kamada T. Alpha1-adrenoceptor activation mediates the infarct size-limiting effect of ischemic preconditioning through augmentation of 5'-nucleotidase activity. *Journal of Clinical Investigation*. 1994;93:2197-2205
190. Kitakaze M, Node K, Minamino T, Komamura K, Funaya H, Shinozaki Y, Chujo M, Mori H, Inoue M, Hori M, Kamada T. Role of activation of protein kinase c in the infarct size-limiting effect of ischemic preconditioning through activation of ecto-5'-nucleotidase. *Circulation*. 1996;93:781-791
191. Kitakaze M, Minamino T, Node K, Komamura K, Shinozaki Y, Chujo M, Mori H, Inoue M, Hori M, Kamada T. Role of activation of ectosolic 5'-nucleotidase in the cardioprotection mediated by opening of k+c channels. *The American journal of physiology*. 1996;270:H1744-1756
192. Kitakaze M, Node K, Asanuma H, Takashima S, Sakata Y, Asakura M, Sanada S, Shinozaki Y, Mori H, Kuzuya T, Hori M. Protein tyrosine kinase is not involved in the infarct size-limiting effect of ischemic preconditioning in canine hearts. *Circulation research*. 2000;87:303-308
193. Kocoglu H, Karaaslan K, Gonca E, Bozdogan O, Gulcu N. Preconditionin effects of dexmedetomidine on myocardial ischemia/reperfusion injury in rats. *Current Therapeutic Research - Clinical and Experimental*. 2008;69:150-158
194. Kohler D, Eckle T, Faigle M, Grenz A, Mittelbronn M, Laucher S, Hart ML, Robson SC, Muller CE, Eltzschig HK. Cd39/ectonucleoside triphosphate diphosphohydrolase 1 provides myocardial protection during cardiac ischemia/reperfusion injury. *Circulation*. 2007;116:1784-1794
195. Kojima M, Kusumoto K, Fujiwara S, Watanabe T, Fujino M. Role of endogenous endothelin in the extension of myocardial infarct size studied with the endothelin receptor antagonist, tak-044. *Journal of cardiovascular pharmacology*. 1995;26:S365-368

196. Kolettis TM, Vilaeti AD, Tsalikakis DG, Zoga A, Valenti M, Tzallas AT, Papalois A, Iliodromitis EK. Effects of pre- and postconditioning on arrhythmogenesis in the in vivo rat model. *Journal of cardiovascular pharmacology and therapeutics*. 2013;18:376-385
197. Koneru S, Penumathsa SV, Thirunavukkarasu M, Samuel SM, Zhan L, Han Z, Maulik G, Das DK, Maulik N. Redox regulation of ischemic preconditioning is mediated by the differential activation of caveolins and their association with enos and glut-4. *American journal of physiology. Heart and circulatory physiology*. 2007;292:2
198. Koning MM, De Zeeuw S, Nieukoop S, De Jong JW, Verdouw PD. Is myocardial infarct size limitation by ischemic preconditioning an "all or nothing" phenomenon? *Ann N Y Acad Sci*. 1994;723:333-336
199. Koning MM, Simonis LA, de Zeeuw S, Nieukoop S, Post S, Verdouw PD. Ischaemic preconditioning by partial occlusion without intermittent reperfusion. *Cardiovascular research*. 1994;28:1146-1151
200. Kositprapa C, Ockaili RA, Kukreja RC. Bradykinin b2 receptor is involved in the late phase of preconditioning in rabbit heart. *Journal of molecular and cellular cardiology*. 2001;33:1355-1362
201. Kouchi I, Murakami T, Nawada R, Akao M, Sasayama S. Katp channels are common mediators of ischemic and calcium preconditioning in rabbits. *The American journal of physiology*. 1998;274:H1106-1112
202. Kremastinos DT, Bofilis E, Karavolias GK, Papalois A, Kaklamanis L, Iliodromitis EK. Preconditioning limits myocardial infarct size in hypercholesterolemic rabbits. *Atherosclerosis*. 2000;150:81-89
203. Kristensen J, Mortensen UM, Nielsen SS, Maeng M, Kaltoft A, Nielsen TT, Rehling M. Myocardial perfusion imaging with 99mtc sestamibi early after reperfusion reliably reflects infarct size reduction by ischaemic preconditioning in an experimental porcine model. *Nucl Med Commun*. 2004;25:495-500
204. Kudej RK, Shen YT, Peppas AP, Huang CH, Chen W, Yan L, Vatner DE, Vatner SF. Obligatory role of cardiac nerves and alpha1-adrenergic receptors for the second window of ischemic preconditioning in conscious pigs. *Circulation research*. 2006;99:1270-1276
205. Kunuthur SP, Mocanu MM, Hemmings BA, Hausenloy DJ, Yellon DM. The akt1 isoform is an essential mediator of ischemic preconditioning. *Journal of cellular and molecular medicine*. 2012;28:1582-4934
206. Kuzmin AI, Gourine AV, Molosh AI, Lakomkin VL, Vassort G. Effects of preconditioning on myocardial interstitial levels of atp and its catabolites during regional ischemia and reperfusion in the rat. *Basic research in cardiology*. 2000;95:127-136
207. Kuzume K, Wolff RA, Chien GL, Van Winkle DM. Remifentanil limits infarct size but attenuates preconditioning-induced infarct limitation. *Coron Artery Dis*. 2004;15:449-455
208. Kuzuya T, Hoshida S, Yamashita N, Fuji H, Oe H, Hori M, Kamada T, Tada M. Delayed effects of sublethal ischemia on the acquisition of tolerance to ischemia. *Circulation research*. 1993;72:1293-1299
209. Landim MB, Dourado PM, Casella-Filho A, Chagas AC, da-Luz PL. High plasma concentrations of asymmetric dimethylarginine inhibit ischemic cardioprotection in hypercholesterolemic rats. *Brazilian journal of medical and biological research.* 2013;46:454-459
210. Lang SC, Elsasser A, Scheler C, Vetter S, Tiefenbacher CP, Kubler W, Katus HA, Vogt AM. Myocardial preconditioning and remote renal preconditioning--identifying a protective factor using proteomic methods? *Basic research in cardiology*. 2006;101:149-158
211. Lange M, Smul TM, Blomeyer CA, Redel A, Klotz KN, Roewer N, Kehl F. Role of the beta1-adrenergic pathway in anesthetic and ischemic preconditioning against myocardial infarction in the rabbit heart in vivo. *Anesthesiology*. 2006;105:503-510
212. Lankford AR, Yang JN, Rose'Meyer R, French BA, Matherne GP, Fredholm BB, Yang Z. Effect of modulating cardiac a1 adenosine receptor expression on protection with ischemic preconditioning. *American journal of physiology. Heart and circulatory physiology*. 2006;290:18
213. Larsen JR, Aagaard SR, Hasenkam JM, Sloth E. Pre-occlusion ischaemia, not sevoflurane, successfully preconditions the myocardium against further damage in porcine in vivo hearts. *Acta Anaesthesiol Scand*. 2007;51:402-409
214. Lasley RD, Konyn PJ, Hegge JO, Mentzer RM, Jr. Effects of ischemic and adenosine preconditioning on interstitial fluid adenosine and myocardial infarct size. *The American journal of physiology*. 1995;269:H1460-1466
215. Lazou A, Iliodromitis EK, Cieslak D, Voskarides K, Mousikos S, Bofilis E, Kremastinos DT. Ischemic but not mechanical preconditioning attenuates ischemia/reperfusion induced myocardial apoptosis in anaesthetized rabbits: The role of bcl-2 family proteins and erk1/2. *Apoptosis*. 2006;11:2195-2204
216. Lee TM, Su SF, Tsai CC, Lee YT, Tsai CH. Cardioprotective effects of 17 beta-estradiol produced by activation ofmitochondrial atp-sensitive k(+)channels in canine hearts. *Journal of molecular and cellular cardiology*. 2000;32:1147-1158
217. Lee BH, Choi MJ, Kim WG. Effect of ischemic preconditioning based on different epicardial branching patterns of the left coronary artery in the rabbit heart. *J Invest Surg*. 2004;17:71-79

218. Li GC, Vasquez JA, Gallagher KP, Lucchesi BR. Myocardial protection with preconditioning. *Circulation*. 1990;82:609-619
219. Li Q, Guo Y, Xuan YT, Lowenstein CJ, Stevenson SC, Prabhu SD, Wu WJ, Zhu Y, Bolli R. Gene therapy with inducible nitric oxide synthase protects against myocardial infarction via a cyclooxygenase-2-dependent mechanism. *Circulation research*. 2003;92:741-748
220. Li R, Wong GT, Wong TM, Zhang Y, Xia Z, Irwin MG. Intrathecal morphine preconditioning induces cardioprotection via activation of delta, kappa, and mu opioid receptors in rats. *Anesth Analg*. 2009;108:23-29
221. Li SJ, Wu YN, Kang Y, Yin YQ, Gao WZ, Liu YX, Lou JS. Influence of limb atraumatic ischemic preconditioning on fibrolysis factors in myocardial ischemia/reperfusion injury in rats. [chinese]. *Chinese Pharmacological Bulletin*. 2008;24:1344-1347
222. Li SJ, Wu YN, Kang Y, Yin YQ, Gao WZ, Liu YX, Lou JS. Effects of limb atraumatic ischemic preconditioning on apoptosis induced by myocardial ischemia-reperfusion injury in rats. [chinese]. *Chinese Pharmacological Bulletin*. 2009;25:100-104
223. Li SJ, Wu YN, Kang Y, Yin YQ, Gao WZ, Liu YX, Lou JS. Noninvasive limb ischemic preconditioning protects against myocardial i/r injury in rats. *J Surg Res*. 2010;164:162-168
224. Li W, Jia G, Guo W, Wang H. Nitric oxide opens second window of protection in ischemic preconditioning via induction of heat-shock protein 72. *Chin Med J*. 2003;116:258-262
225. Li XD, Cheng YT, Yang YJ, Meng XM, Zhao JL, Zhang HT, Wu YJ, You SJ, Wu YL. Pka-mediated enos phosphorylation in the protection of ischemic preconditioning against no-reflow. *Microvascular research*. 2012;84:44-54
226. Li XD, Yang YJ, Zhao JL, Zhang HT, Cheng YT, Tian Y, Meng XM, Cui HH, Wang TJ. [ischemic preconditioning attenuates myocardial no-reflow and reperfusion injury after revascularization of acute myocardial infarction by reducing myocardial edema via the protein kinase a pathway]. *Zhonghua Xin Xue Guan Bing Za Zhi*. 2012;40:945-951
227. Li YW, Whittaker P, Kloner RA. The transient nature of the effect of ischemic preconditioning on myocardial infarct size and ventricular arrhythmia. *Am Heart J*. 1992;123:346-353
228. Li Y, Kloner RA. Cardioprotective effects of ischaemic preconditioning are not mediated by prostanoids. *Cardiovascular research*. 1992;26:226-231
229. Li Y, Kloner RA. Adenosine deaminase inhibition is not cardioprotective in the rat. *Am Heart J*. 1993;126:1293-1298
230. Li Y, Kloner RA. The cardioprotective effects of ischemic 'preconditioning' are not mediated by adenosine receptors in rat hearts. *Circulation*. 1993;87:1642-1648
231. Li Y, Kloner RA. Cardioprotective effects of ischemic preconditioning can be recaptured after they are lost. *J Am Coll Cardiol*. 1994;23:470-474
232. Li Y, Kloner RA. Does protein kinase c play a role in ischemic preconditioning in rat hearts? *The American journal of physiology*. 1995;268:H426-431
233. Li Y, Cai M, Xu Y, Swartz HM, He G. Late phase ischemic preconditioning preserves mitochondrial oxygen metabolism and attenuates post-ischemic myocardial tissue hyperoxygenation. *Life Sci*. 2011;88:57-64
234. Liao XL, Hu XX, Chang FJ, Yuan HY, Ci HB, Wu JY, Xu Z, Wang ZP, Zhang X, Xia Z, Ou JS. A simple modification results in greater success in the model of coronary artery ligation and myocardial ischemia in mice. *Journal of cardiovascular pharmacology*. 2013;61:430-436
235. Liaudet L, Yang Z, Al-Affar EB, Szabo C. Myocardial ischemic preconditioning in rodents is dependent on poly (adp-ribose) synthetase. *Mol Med*. 2001;7:406-417
236. Lie RH, Stoettrup N, Sloth E, Hasenkam JM, Kroyer R, Nielsen TT. Post-conditioning with cyclosporine a fails to reduce the infarct size in an in vivo porcine model. *Acta Anaesthesiol Scand*. 2010;54:804-813
237. Liem DA, van den Doel MA, de Zeeuw S, Verdouw PD, Duncker DJ. Role of adenosine in ischemic preconditioning in rats depends critically on the duration of the stimulus and involves both a(1) and a(3) receptors. *Cardiovascular research*. 2001;51:701-708
238. Liem DA, te Lintel Hekkert M, Manintveld OC, Boomsma F, Verdouw PD, Duncker DJ. Myocardium tolerant to an adenosine-dependent ischemic preconditioning stimulus can still be protected by stimuli that employ alternative signaling pathways. *American journal of physiology. Heart and circulatory physiology*. 2005;288:14
239. Lim SY, Davidson SM, Hausenloy DJ, Yellon DM. Preconditioning and postconditioning: The essential role of the mitochondrial permeability transition pore. *Cardiovascular research*. 2007;75:530-535
240. Lim SY, Yellon DM, Hausenloy DJ. The neural and humoral pathways in remote limb ischemic preconditioning. *Basic research in cardiology*. 2010;105:651-655

241. Liu Y, Thornton JD, Cohen MV, Downey JM, Schaffer SW. Streptozotocin-induced non-insulin-dependent diabetes protects the heart from infarction. *Circulation*. 1993;88:1273-1278
242. Liu Y, Downey JM. Preconditioning against infarction in the rat heart does not involve a pertussis toxin sensitive g protein. *Cardiovascular research*. 1993;27:608-611
243. Liu Y, Ytrehus K, Downey JM. Evidence that translocation of protein kinase c is a key event during ischemic preconditioning of rabbit myocardium. *Journal of molecular and cellular cardiology*. 1994;26:661-668
244. Liu Y, Wu WK, Chen C, Liu KX. [role of nf-kappab in the induction of delayed preconditioning in rat heart by pretreatment with sini decoction]. *Zhongguo Zhong Yao Za Zhi*. 2005;30:453-456
245. Liu Z, Barrett HH, Stevenson GD, Furenlid LR, Pak KY, Woolfenden JM. Evaluating the protective role of ischaemic preconditioning in rat hearts using a stationary small-animal spect imager and 99mtc-glucarate. *Nucl Med Commun*. 2008;29:120-128
246. Liu GS, Stanley AW, Downey J. Cyclooxygenase products are not involved in the protection against myocardial infarction afforded by preconditioning in rabbit. Cyclooxygenase pathway's involvement in preconditioning. *Am J Cardiovasc Pathol*. 1992;4:157-164
247. Liu GS, Stanley AW, Downey JM. Ischaemic preconditioning is not dependent on neutrophils or glycolytic substrate at reperfusion in rabbit heart. *Cardiovascular research*. 1992;26:1195-1198
248. Liu Y, Downey JM. Ischemic preconditioning protects against infarction in rat heart. *The American journal of physiology*. 1992;263:H1107-1112
249. Loke KE, Woodman OL. Preconditioning improves myocardial function and reflow, but not vasodilator reactivity, after ischaemia and reperfusion in anaesthetized dogs. *Clin Exp Pharmacol Physiol*. 1998;25:552-558
250. Lott FD, Guo P, Toombs CF. Reduction in infarct size by ischemic preconditioning persists in a chronic rat model of myocardial ischemia-reperfusion injury. *Pharmacology*. 1996;52:113-118
251. Lotz C, Lazariotto M, Redel A, Smul TM, Stumpner J, Blomeyer C, Tischer-Zeitz T, Schmidt J, Pociej J, Roewer N, Kehl F, Lange M. Activation of peroxisome-proliferator-activated receptors alpha and gamma mediates remote ischemic preconditioning against myocardial infarction in vivo. *Exp Biol Med*. 2011;236:113-122
252. Lou JS, Liang HX, Liu YX. The pharmacological preconditioning of nitroglycerin and buprenorphine used in combination against myocardial ischemia in rats: Early cardioprotection. [chinese]. *Chinese Pharmacological Bulletin*. 2003;19:1119-1125
253. Lu XM, Zhang GX, Yu YQ, Kimura S, Nishiyama A, Matsuyoshi H, Shimizu J, Takaki M. The opposite roles of nnos in cardiac ischemia-reperfusion-induced injury and in ischemia preconditioning-induced cardioprotection in mice. *J Physiol Sci*. 2009;59:253-262
254. Lu Y, Dong C, Yu J, Li L. Role of central and peripheral opioid receptors in the cardioprotection of intravenous morphine preconditioning. *Ir J Med Sci*. 2011;180:881-885
255. Lu Y, Dong CS, Yu JM, Li H. Morphine reduces the threshold of remote ischemic preconditioning against myocardial ischemia and reperfusion injury in rats: The role of opioid receptors. *J Cardiothorac Vasc Anesth*. 2012;26:403-406
256. Luo D, Deng PY, Ye F, Peng WJ, Deng HW, Li YJ. Delayed preconditioning by cardiac ischemia involves endogenous calcitonin gene-related peptide via the nitric oxide pathway. *European journal of pharmacology*. 2004;502:135-141
257. Manintveld OC, te Lintel Hekkert M, Keijzer E, Verdouw PD, Duncker DJ. Intravenous adenosine protects the myocardium primarily by activation of a neurogenic pathway. *British journal of pharmacology*. 2005;145:703-711
258. Manintveld OC, Sluiter W, Dekkers DH, te Lintel Hekkert M, Lamers JM, Verdouw PD, Duncker DJ. Involvement of reperfusion injury salvage kinases in preconditioning depends critically on the preconditioning stimulus. *Exp Biol Med*. 2011;236:874-882
259. Marber MS, Latchman DS, Walker JM, Yellon DM. Cardiac stress protein elevation 24 hours after brief ischemia or heat stress is associated with resistance to myocardial infarction. *Circulation*. 1993;88:1264-1272
260. Martin BJ, McClanahan TB, Van Wylen DG, Gallagher KP. Effects of ischemia, preconditioning, and adenosine deaminase inhibition on interstitial adenosine levels and infarct size. *Basic research in cardiology*. 1997;92:240-251
261. Matsuda M, Catena TG, Vander Heide RS, Jennings RB, Reimer KA. Cardiac protection by ischaemic preconditioning is not mediated by myocardial stunning. *Cardiovascular research*. 1993;27:585-592
262. Matsumura K, Komori S, Takusagawa M, Osada M, Tanabe F, Itoh M, Tamura K. Protein kinase c is involved in cardioprotective effects of ischemic preconditioning on infarct size and ventricular arrhythmia in rats in vivo. *Mol Cell Biochem*. 2000;214:39-45

263. McVey M, Perrone MH, Clark KL. Does tumor necrosis factor-alpha (tnf-alpha) contribute to myocardial reperfusion injury in anesthetized rats? *Gen Pharmacol*. 1999;32:41-45
264. McVey MJ, Smits GJ, Cox BF, Kitzen JM, Clark KL, Perrone MH. Cardiovascular pharmacology of the adenosine a1/a2-receptor agonist amp 579: Coronary hemodynamic and cardioprotective effects in the canine myocardium. *Journal of cardiovascular pharmacology*. 1999;33:703-710
265. Mei DA, Nithipatikom K, Lasley RD, Gross GJ. Myocardial preconditioning produced by ischemia, hypoxia, and a katp channel opener: Effects on interstitial adenosine in dogs. *Journal of molecular and cellular cardiology*. 1998;30:1225-1236
266. Miki T, Miura T, Ura N, Ogawa T, Suzuki K, Shimamoto K, Iimura O. Captopril potentiates the myocardial infarct size-limiting effect of ischemic preconditioning through bradykinin b2 receptor activation. *J Am Coll Cardiol*. 1996;28:1616-1622
267. Miki T, Cohen MV, Downey JM. Opioid receptor contributes to ischemic preconditioning through protein kinase c activation in rabbits. *Mol Cell Biochem*. 1998;186:3-12
268. Miki T, Miura T, Bunger R, Suzuki K, Sakamoto J, Shimamoto K. Ecto-5'-nucleotidase is not required for ischemic preconditioning in rabbit myocardium in situ. *The American journal of physiology*. 1998;275:H1329-1337
269. Miki T, Swafford AN, Cohen MV, Downey JM. Second window of protection against infarction in conscious rabbits: Real or artifactual. *Journal of molecular and cellular cardiology*. 1999;31:809-816
270. Miller DL, Van Winkle DM. Ischemic preconditioning limits infarct size following regional ischemia-reperfusion in in situ mouse hearts. *Cardiovascular research*. 1999;42:680-684
271. Minamino T, Kitakaze M, Morioka T, Node K, Komamura K, Takeda H, Inoue M, Hori M, Kamada T. Cardioprotection due to preconditioning correlates with increased ecto-5'-nucleotidase activity. *The American journal of physiology*. 1996;270:H238-244
272. Miura T, Goto M, Urabe K, Endoh A, Shimamoto K, Iimura O. Does myocardial stunning contribute to infarct size limitation by ischemic preconditioning? *Circulation*. 1991;84:2504-2512
273. Miura T, Ogawa T, Iwamoto T, Shimamoto K, Iimura O. Dipyridamole potentiates the myocardial infarct size-limiting effect of ischemic preconditioning. *Circulation*. 1992;86:979-985
274. Miura T, Kawamura S, Goto M, Sakamoto J, Tsuchida A, Matsuzaki M, Shimamoto K. Effect of protein kinase c inhibitors on cardioprotection by ischemic preconditioning depends on the number of preconditioning episodes. *Cardiovascular research*. 1998;37:700-709
275. Mizumura T, Nithipatikom K, Gross GJ. Bimakalim, an atp-sensitive potassium channel opener, mimics the effects of ischemic preconditioning to reduce infarct size, adenosine release, and neutrophil function in dogs. *Circulation*. 1995;92:1236-1245
276. Mizumura T, Auchampach JA, Linden J, Bruns RF, Gross GJ. Pd 81,723, an allosteric enhancer of the a1 adenosine receptor, lowers the threshold for ischemic preconditioning in dogs. *Circulation research*. 1996;79:415-423
277. Mizumura T, Saito S, Ozawa Y, Kanmatsuse K, Gross GJ. An atp-sensitive potassium (katp) channel opener, nicorandil, lowers the threshold for ischemic preconditioning in barbital-anesthetized dogs. *Heart Vessels*. 1997;12:175-177
278. Morgan EN, Boyle EM, Jr., Yun W, Griscavage-Ennis JM, Farr AL, Canty TG, Jr., Pohlman TH, Verrier ED. An essential role for nf-kappab in the cardioadaptive response to ischemia. *Ann Thorac Surg*. 1999;68:377-382
279. Mortensen UM, Norgaard BL, Nielsen-Kudsk JE, Kristensen J, Maeng M, Nielsen TT. A phase of increased st elevation during coronary occlusion following ischemic preconditioning. *Basic research in cardiology*. 2006;101:140-148
280. Mullenheim J, Frassdorf J, Preckel B, Thamer V, Schlack W. Ketamine, but not s(+)-ketamine, blocks ischemic preconditioning in rabbit hearts in vivo. *Anesthesiology*. 2001;94:630-636
281. Mullenheim J, Rulands R, Wietschorke T, Frassdorf J, Preckel B, Schlack W. Late preconditioning is blocked by racemic ketamine, but not by s(+)-ketamine. *Anesth Analg*. 2001;93:265-270
282. Mullenheim J, Schlack W, Frassdorf J, Heinen A, Preckel B, Thamer V. Additive protective effects of late and early ischaemic preconditioning are mediated by the opening of katp channels in vivo. *Pflugers Arch*. 2001;442:178-187
283. Mullenheim J, Ebel D, Bauer M, Otto F, Heinen A, Frassdorf J, Preckel B, Schlack W. Sevoflurane confers additional cardioprotection after ischemic late preconditioning in rabbits. *Anesthesiology*. 2003;99:624-631
284. Munch-Ellingsen J, Bugge E, Lokebo JE, Ytrehus K. Potassium channel blocker dofetilide does not abolish ischaemic preconditioning. *Scand J Clin Lab Invest*. 1997;57:13-20
285. Munch-Ellingsen J, Lokebo JE, Bugge B, Ytrehus K. Equal reduction in infarct size by ethylisopropyl amiloride pretreatment and ischemic preconditioning in the in situ rabbit heart. *Molecular and Cellular Biochemistry*. 1998;186:13-18

286. Murry CE, Jennings RB, Reimer KA. Preconditioning with ischemia: A delay of lethal cell injury in ischemic myocardium. *Circulation*. 1986;74:1124-1136
287. Murry CE, Richard VJ, Jennings RB, Reimer KA. Myocardial protection is lost before contractile function recovers from ischemic preconditioning. *The American journal of physiology*. 1991;260:H796-804
288. Nadtochiy SM, Burwell LS, Ingraham CA, Spencer CM, Friedman AE, Pinkert CA, Brookes PS. In vivo cardioprotection by s-nitroso-2-mercaptopropionyl glycine. *Journal of molecular and cellular cardiology*. 2009;46:960-968
289. Nakae I, Takaoka A, Mitsunami K, Yabe T, Ito M, Masayuki, Takahashi, Matsumoto T, Omura T, Yokohama H, Kinoshita M. Cardioprotective effects of nicorandil in rabbits anaesthetized with halothane: Potentiation of ischaemic preconditioning via katp channels. *Clin Exp Pharmacol Physiol*. 2000;27:810-817
290. Nakano A, Miura T, Ura N, Suzuki K, Shimamoto K. Role of the angiotensin ii type 1 receptor in preconditioning against infarction. *Coron Artery Dis*. 1997;8:343-350
291. Naumenko SE, Latysheva TV, Gilinskii MA. Inhibition of norepinephrine reuptake and size of myocardial infarction during focal ischemia and after preconditioning. *Bull Exp Biol Med*. 2010;150:15-17
292. Nawada R, Murakami T, Iwase T, Nagai K, Morita Y, Kouchi I, Akao M, Sasayama S. Inhibition of sarcolemmal na+,k+-atpase activity reduces the infarct size-limiting effect of preconditioning in rabbit hearts. *Circulation*. 1997;96:599-604
293. Neckar J, Papousek F, Novakova O, Ost'adal B, Kolar F. Cardioprotective effects of chronic hypoxia and ischaemic preconditioning are not additive. *Basic research in cardiology*. 2002;97:161-167
294. Nieszner E, Posa I, Kocsis E, Pogatsa G, Preda I, Koltai MZ. Influence of diabetic state and that of different sulfonylureas on the size of myocardial infarction with and without ischemic preconditioning in rabbits. *Exp Clin Endocrinol Diabetes*. 2002;110:212-218
295. Nishihara M, Miura T, Miki T, Sakamoto J, Tanno M, Kobayashi H, Ikeda Y, Ohori K, Takahashi A, Shimamoto K. Erythropoietin affords additional cardioprotection to preconditioned hearts by enhanced phosphorylation of glycogen synthase kinase-3 beta. *American journal of physiology. Heart and circulatory physiology*. 2006;291:24
296. Nithipatikom K, Endsley MP, Moore JM, Isbell MA, Falck JR, Campbell WB, Gross GJ. Effects of selective inhibition of cytochrome p-450 omega-hydroxylases and ischemic preconditioning in myocardial protection. *American journal of physiology. Heart and circulatory physiology*. 2006;290:7
297. Nithipatikom K, Moore JM, Isbell MA, Falck JR, Gross GJ. Epoxyeicosatrienoic acids in cardioprotection: Ischemic versus reperfusion injury. *American journal of physiology. Heart and circulatory physiology*. 2006;291:10
298. Node K, Kitakaze M, Minamino T, Tada M, Inoue M, Hori M, Kamada T. Activation of ecto-5'-nucleotidase by protein kinase c and its role in ischaemic tolerance in the canine heart. *British journal of pharmacology*. 1997;120:273-281
299. Nozawa Y, Miura T, Tsuchida A, Kita H, Fukuma T, Shimamoto K. Chronic treatment with an ace inhibitor, temocapril, lowers the threshold for the infarct size-limiting effect of ischemic preconditioning. *Cardiovasc Drugs Ther*. 1999;13:151-157
300. Nozawa Y, Miura T, Miki T, Ohnuma Y, Yano T, Shimamoto K. Mitochondrial katp channel-dependent and -independent phases of ischemic preconditioning against myocardial infarction in the rat. *Basic research in cardiology*. 2003;98:50-58
301. Okamura T, Miura T, Iwamoto H, Shirakawa K, Kawamura S, Ikeda Y, Iwatate M, Matsuzaki M. Ischemic preconditioning attenuates apoptosis through protein kinase c in rat hearts. *The American journal of physiology*. 1999;277:H1997-2001
302. Okubo S, Bernardo NL, Elliott GT, Hess ML, Kukreja RC. Tyrosine kinase signaling in action potential shortening and expression of hsp72 in late preconditioning. *American journal of physiology. Heart and circulatory physiology*. 2000;279:H2269-2276
303. Okubo S, Tanabe Y, Takeda K, Kitayama M, Kanemitsu S, Kukreja RC, Takekoshi N. Ischemic preconditioning and morphine attenuate myocardial apoptosis and infarction after ischemia-reperfusion in rabbits: Role of delta-opioid receptor. *American journal of physiology. Heart and circulatory physiology*. 2004;287:1
304. Okubo S, Tanabe Y, Takeda K, Kitayama M, Kanemitsu S, Kukreja RC, Takekoshi N. Pretreatment with tyrosine kinase inhibitor attenuates the reduction of apoptosis 24 h after ischemic preconditioning. *Jpn J Physiol*. 2004;54:143-151
305. Ovize M, Kloner RA, Hale SL, Przyklenk K. Coronary cyclic flow variations "precondition" ischemic myocardium. *Circulation*. 1992;85:779-789
306. Ovize M, Aupetit JF, Rioufol G, Loufoua J, Andre-Fouet X, Minaire Y, Faucon G. Preconditioning reduces infarct size but accelerates time to ventricular fibrillation in ischemic pig heart. *The American journal of physiology*. 1995;269:H72-79

307. Pagel PS, Krolikowski JG, Shim YH, Venkatapuram S, Kersten JR, Weihrauch D, Warltier DC, Pratt PF, Jr. Noble gases without anesthetic properties protect myocardium against infarction by activating prosurvival signaling kinases and inhibiting mitochondrial permeability transition in vivo. *Anesth Analg*. 2007;105:562-569
308. Patel HH, Hsu A, Moore J, Gross GJ. Bw373u86, a delta opioid agonist, partially mediates delayed cardioprotection via a free radical mechanism that is independent of opioid receptor stimulation. *Journal of molecular and cellular cardiology*. 2001;33:1455-1465
309. Patel HH, Moore J, Hsu AK, Gross GJ. Cardioprotection at a distance: Mesenteric artery occlusion protects the myocardium via an opioid sensitive mechanism. *Journal of molecular and cellular cardiology*. 2002;34:1317-1323
310. Patel HH, Gross ER, Peart JN, Hsu AK, Gross GJ. Sarcolemmal katp channel triggers delayed ischemic preconditioning in rats. *American journal of physiology. Heart and circulatory physiology*. 2005;288:24
311. Peart JN, Patel HH, Gross GJ. Delta-opioid receptor activation mimics ischemic preconditioning in the canine heart. *Journal of cardiovascular pharmacology*. 2003;42:78-81
312. Pell TJ, Baxter GF, Yellon DM, Drew GM. Renal ischemia preconditions myocardium: Role of adenosine receptors and atp-sensitive potassium channels. *The American journal of physiology*. 1998;275:H1542-1547
313. Perricone AJ, Bivona BJ, Jackson FR, Vander Heide RS. Conditional knockout of myocyte focal adhesion kinase abrogates ischemic preconditioning in adult murine hearts. *Journal of the American Heart Association*. 2013;2:e000457
314. Petrischev NN, Shlyakhto Ye V, Tsyrlin VA, Vlasov TD, Syrensky AV, Galagudza MM. The role of oxygen free radicals in the mechanisms of local and distant ischemic myocardial preconditioning. [russian]. *Vestnik Rossiiskoi Akademii Meditsinskikh Nauk*. 2006:10-15
315. Ping P, Takano H, Zhang J, Tang XL, Qiu Y, Li RC, Banerjee S, Dawn B, Balafonova Z, Bolli R. Isoform-selective activation of protein kinase c by nitric oxide in the heart of conscious rabbits: A signaling mechanism for both nitric oxide-induced and ischemia-induced preconditioning. *Circulation research*. 1999;84:587-604
316. Ping P, Song C, Zhang J, Guo Y, Cao X, Li RC, Wu W, Vondriska TM, Pass JM, Tang XL, Pierce WM, Bolli R. Formation of protein kinase c(epsilon)-lck signaling modules confers cardioprotection. *The Journal of clinical investigation*. 2002;109:499-507
317. Piot CA, Padmanaban D, Ursell PC, Sievers RE, Wolfe CL. Ischemic preconditioning decreases apoptosis in rat hearts in vivo. *Circulation*. 1997;96:1598-1604
318. Poulsen RH, Rasmussen JT, Botker HE, Waehrens LS, Falborg L, Heegaard CW, Rehling M. Imaging the myocardium at risk with (9)(9)mtc-lactadherin administered after reperfusion in a porcine model. *Nuclear medicine and biology*. 2014;41:114-119
319. Przyklenk K, Bauer B, Ovize M, Kloner RA, Whittaker P. Regional ischemic 'preconditioning' protects remote virgin myocardium from subsequent sustained coronary occlusion. *Circulation*. 1993;87:893-899
320. Przyklenk K, Sussman MA, Simkhovich BZ, Kloner RA. Does ischemic preconditioning trigger translocation of protein kinase c in the canine model? *Circulation*. 1995;92:1546-1557
321. Przyklenk K, Zhao L, Kloner RA, Elliott GT. Cardioprotection with ischemic preconditioning and mla: Role of adenosine-regulating enzymes? *The American journal of physiology*. 1996;271:H1004-1014
322. Przyklenk K, Hata K, Kloner RA. Is calcium a mediator of infarct size reduction with preconditioning in canine myocardium? *Circulation*. 1997;96:1305-1312
323. Przyklenk K, Li G, Whittaker P. No loss in the in vivo efficacy of ischemic preconditioning in middle-aged and old rabbits. *J Am Coll Cardiol*. 2001;38:1741-1747
324. Przyklenk K, Li G, Simkhovich BZ, Kloner RA. Mechanisms of myocardial ischemic preconditioning are age related: Pkc-epsilon does not play a requisite role in old rabbits. *J Appl Physiol*. 2003;95:2563-2569
325. Qian YZ, Levasseur JE, Yoshida KI, Kukreja RC. K(atp) channels in rat heart: Blockade of ischemic and acetylcholine- mediated preconditioning by glibenclamide. *American Journal of Physiology - Heart and Circulatory Physiology*. 1996;271:H23-H28
326. Qian YZ, Bernardo NL, Nayeem MA, Chelliah J, Kukreja RC. Induction of 72-kda heat shock protein does not produce second window of ischemic preconditioning in rat heart. *The American journal of physiology*. 1999;276:H224-234
327. Qiu Y, Rizvi A, Tang XL, Manchikalapudi S, Takano H, Jadoon AK, Wu WJ, Bolli R. Nitric oxide triggers late preconditioning against myocardial infarction in conscious rabbits. *The American journal of physiology*. 1997;273:H2931-2936
328. Qiu Y, Tang XL, Park SW, Sun JZ, Kalya A, Bolli R. The early and late phases of ischemic preconditioning: A comparative analysis of their effects on infarct size, myocardial stunning, and arrhythmias in conscious pigs undergoing a 40-minute coronary occlusion. *Circulation research*. 1997;80:730-742

329. Rajesh KG, Sasaguri S, Zhitian Z, Suzuki R, Asakai R, Maeda H. Second window of ischemic preconditioning regulates mitochondrial permeability transition pore by enhancing bcl-2 expression. *Cardiovascular research*. 2003;59:297-307

330. Rajesh KG, Sasaguri S, Suzuki R, Xing Y, Maeda H. Ischemic preconditioning prevents reperfusion heart injury in cardiac hypertrophy by activation of mitochondrial katp channels. *Int J Cardiol*. 2004;96:41-49

331. Raphael J, Drenger B, Rivo J, Berenshtein E, Chevion M, Gozal Y. Ischemic preconditioning decreases the reperfusion-related formation of hydroxyl radicals in a rabbit model of regional myocardial ischemia and reperfusion: The role of k(atp) channels. *Free Radic Res*. 2005;39:747-754

332. Redel A, Stumpner J, Tischer-Zeitz T, Lange M, Smul TM, Lotz C, Roewer N, Kehl F. Comparison of isoflurane-, sevoflurane-, and desflurane-induced pre- and postconditioning against myocardial infarction in mice in vivo. *Exp Biol Med*. 2009;234:1186-1191

333. Reffelmann T, Kloner RA. Is microvascular protection by cariporide and ischemic preconditioning causally linked to myocardial salvage? *American journal of physiology. Heart and circulatory physiology*. 2003;284:10

334. Ren X, Wang Y, Jones WK. Tnf-alpha is required for late ischemic preconditioning but not for remote preconditioning of trauma. *J Surg Res*. 2004;121:120-129

335. Richard V, Tron C, Thuillez C. Ischaemic preconditioning is not mediated by oxygen derived free radicals in rats. *Cardiovascular research*. 1993;27:2016-2021

336. Richard V, Kaeffer N, Tron C, Thuillez C. Ischemic preconditioning protects against coronary endothelial dysfunction induced by ischemia and reperfusion. *Circulation*. 1994;89:1254-1261

337. Rioufol G, Ovize M, Loufoua J, Pop C, Andre-Fouat X, Minaire Y. Ventricular fibrillation in preconditioned pig hearts: Role of k+atp channels. *The American journal of physiology*. 1997;273:H2804-2810

338. Rivo J, Raphael J, Drenger B, Berenshtein E, Chevion M, Gozal Y. Flumazenil mimics whereas midazolam abolishes ischemic preconditioning in a rabbit heart model of ischemia-reperfusion. *Anesthesiology*. 2006;105:65-71

339. Roesner JP, Petzelbauer P, Koch A, Mersmann J, Zacharowski PA, Boehm O, Reingruber S, Pasteiner W, Mascher D, Wolzt M, Barthuber C, Noldge-Schomburg GE, Scheeren TW, Zacharowski K. The fibrin-derived peptide bbeta15-42 is cardioprotective in a pig model of myocardial ischemia-reperfusion injury. *Crit Care Med*. 2007;35:1730-1735

340. Roesner JP, Mersmann J, Bergt S, Bohnenberg K, Barthuber C, Szabo C, Noldge-Schomburg GE, Zacharowski K. Therapeutic injection of parp inhibitor ino-1001 preserves cardiac function in porcine myocardial ischemia and reperfusion without reducing infarct size. *Shock*. 2010;33:507-512

341. Rohmann S, Weygandt H, Schelling P, Kie Soei L, Verdouw PD, Lues I. Involvement of atp-sensitive potassium channels in preconditioning protection. *Basic research in cardiology*. 1994;89:563-576

342. Sack S, Mohri M, Arras M, Schwarz ER, Schaper W. Ischaemic preconditioning--time course of renewal in the pig. *Cardiovascular research*. 1993;27:551-555

343. Sanada S, Kitakaze M, Asanuma H, Harada K, Ogita H, Node K, Takashima S, Sakata Y, Asakura M, Shinozaki Y, Mori H, Kuzuya T, Hori M. Role of mitochondrial and sarcolemmal k(atp) channels in ischemic preconditioning of the canine heart. *American journal of physiology. Heart and circulatory physiology*. 2001;280:H256-263

344. Sanada S, Kitakaze M, Papst PJ, Hatanaka K, Asanuma H, Aki T, Shinozaki Y, Ogita H, Node K, Takashima S, Asakura M, Yamada J, Fukushima T, Ogai A, Kuzuya T, Mori H, Terada N, Yoshida K, Hori M. Role of phasic dynamism of p38 mitogen-activated protein kinase activation in ischemic preconditioning of the canine heart. *Circulation research*. 2001;88:175-180

345. Sanada S, Asanuma H, Tsukamoto O, Minamino T, Node K, Takashima S, Fukushima T, Ogai A, Shinozaki Y, Fujita M, Hirata A, Okuda H, Shimokawa H, Tomoike H, Hori M, Kitakaze M. Protein kinase a as another mediator of ischemic preconditioning independent of protein kinase c. *Circulation*. 2004;110:51-57

346. Sandhu R, Diaz RJ, Mao GD, Wilson GJ. Ischemic preconditioning: Differences in protection and susceptibility to blockade with single-cycle versus multicycle transient ischemia. *Circulation*. 1997;96:984-995

347. Sanz E, Garcia Dorado D, Oliveras J, Barrabes JA, Gonzalez MA, Ruiz-Meana M, Solares J, Carreras MJ, Garcia-Lafuente A, Desco M, et al. Dissociation between anti-infarct effect and anti-edema effect of ischemic preconditioning. *The American journal of physiology*. 1995;268:H233-241

348. Sarkar K, Cai Z, Gupta R, Parajuli N, Fox-Talbot K, Darshan MS, Gonzalez FJ, Semenza GL. Hypoxia-inducible factor 1 transcriptional activity in endothelial cells is required for acute phase cardioprotection induced by ischemic preconditioning. *Proc Natl Acad Sci U S A*. 2012;109:10504-10509

349. Sasamori J, Aihara K, Yoneyama F, Sato I, Kogi K, Takeo S. Amelioration of ischemia/reperfusion-induced myocardial infarction by the 2-alkynyladenosine derivative 2-octynyladenosine (yt-146). *Journal of cardiovascular pharmacology*. 2006;47:614-620

350. Sato H, Bolli R, Rokosh GD, Bi Q, Dai S, Shirk G, Tang XL. The cardioprotection of the late phase of ischemic preconditioning is enhanced by postconditioning via a cox-2-mediated mechanism in conscious rats. *American journal of physiology. Heart and circulatory physiology*. 2007;293:17
351. Sbarouni E, Iliodromitis EK, Zoga A, Vlachou G, Andreadou I, Kremastinos DT. The effect of the phytoestrogen genistein on myocardial protection, preconditioning and oxidative stress. *Cardiovasc Drugs Ther*. 2006;20:253-258
352. Schmidt MR, Stottrup NB, Contractor H, Hyldebrandt JA, Johannsen M, Pedersen CM, Birkler R, Ashrafian H, Sorensen KE, Kharbanda RK, Redington AN, Botker HE. Remote ischemic preconditioning with--but not without--metabolic support protects the neonatal porcine heart against ischemia-reperfusion injury. *Int J Cardiol*. 2014;170:388-393
353. Schoemaker RG, van Heijningen CL. Bradykinin mediates cardiac preconditioning at a distance. *American journal of physiology. Heart and circulatory physiology*. 2000;278:H1571-1576
354. Schott RJ, Schaper W. Ischemic preconditioning and myocardial stunning: Related consequences of brief coronary occlusion and reperfusion? *Adv Cardiol*. 1990;37:32-41
355. Schultz JEJ, Rose E, Yao Z, Gross GJ. Evidence for involvement of opioid receptors in ischemic preconditioning in rat hearts. *American Journal of Physiology - Heart and Circulatory Physiology*. 1995;268:H2157-H2161
356. Schultz JEJ, Hsu AK, Gross GJ. Morphine mimics the cardioprotective effects of ischemic preconditioning via a glibenclamide-sensitive mechanism in the rat heart. *Circulation research*. 1996;78:1100-1104
357. Schultz JE, Qian YZ, Gross GJ, Kukreja RC. The ischemia-selective katp channel antagonist, 5-hydroxydecanoate, blocks ischemic preconditioning in the rat heart. *Journal of molecular and cellular cardiology*. 1997;29:1055-1060
358. Schultz JE, Yao Z, Cavero I, Gross GJ. Glibenclamide-induced blockade of ischemic preconditioning is time dependent in intact rat heart. *The American journal of physiology*. 1997;272:H2607-2615
359. Schultz JJ, Hsu AK, Gross GJ. Ischemic preconditioning is mediated by a peripheral opioid receptor mechanism in the intact rat heart. *Journal of molecular and cellular cardiology*. 1997;29:1355-1362
360. Schultz JJ, Hsu AK, Gross GJ. Ischemic preconditioning and morphine-induced cardioprotection involve the delta (delta)-opioid receptor in the intact rat heart. *Journal of molecular and cellular cardiology*. 1997;29:2187-2195
361. Schultz JE, Hsu AK, Barbieri JT, Li PL, Gross GJ. Pertussis toxin abolishes the cardioprotective effect of ischemic preconditioning in intact rat heart. *The American journal of physiology*. 1998;275:H495-500
362. Schultz JE, Hsu AK, Gross GJ. Ischemic preconditioning in the intact rat heart is mediated by delta1- but not mu- or kappa-opioid receptors. *Circulation*. 1998;97:1282-1289
363. Schultz JE, Kwok WM, Hsu AK, Gross GJ. Terikalant, an inward-rectifier potassium channel blocker, does not abolish the cardioprotection induced by ischemic preconditioning in the rat. *Journal of molecular and cellular cardiology*. 1998;30:1817-1825
364. Schulz R, Rose J, Heusch G. Involvement of activation of atp-dependent potassium channels in ischemic preconditioning in swine. *The American journal of physiology*. 1994;267:H1341-1352
365. Schwartz LM, Jennings RB, Reimer KA. Premedication with the opioid analgesic butorphanol raises the threshold for ischemic preconditioning in dogs. *Basic research in cardiology*. 1997;92:106-114
366. Schwartz LM, Sebbag L, Jennings RB, Reimer KA. Duration and reinstatement of myocardial protection against infarction by ischemic preconditioning in open chest dogs. *Journal of molecular and cellular cardiology*. 2001;33:1561-1570
367. Schwartz LM, Welch TS, Crago MS. Cardioprotection by multiple preconditioning cycles does not require mitochondrial k(atp) channels in pigs. *American journal of physiology. Heart and circulatory physiology*. 2002;283:27
368. Schwarz ER, Montino H, Fleischhauer J, Klues HG, vom Dahl J, Hanrath P. Angiotensin ii receptor antagonist exp 3174 reduces infarct size comparable with enalaprilat and augments preconditioning in the pig heart. *Cardiovasc Drugs Ther*. 1997;11:687-695
369. Schwarz ER, Fleischhauer J, Montino H, Chakupurakal R, Foresti M, Schuetz T, Sack S, Mohri M, Arras M, Schaper W, Hanrath P. Infarct size reduction by ischemic preconditioning is a monophasic, short-lived phenomenon in anesthetized pigs. *Journal of cardiovascular pharmacology and therapeutics*. 1998;3:63-70
370. Sebbag L, Katsuragawa M, Verbinski S, Jennings RB, Reimer KA. Intracoronary administration of the alpha 1-receptor agonist, methoxamine, does not reproduce the infarct-limiting effect of ischemic preconditioning in dogs. *Cardiovascular research*. 1996;32:830-838
371. Shahid M, Tauseef M, Sharma KK, Fahim M. Brief femoral artery ischaemia provides protection against myocardial ischaemia-reperfusion injury in rats: The possible mechanisms. *Exp Physiol*. 2008;93:954-968

372. Shattock MJ, Lawson CS, Hearse DJ, Downey JM. Electrophysiological characteristics of repetitive ischemic preconditioning in the pig heart. *Journal of molecular and cellular cardiology*. 1996;28:1339-1347
373. Shekarforoush S, Noroozzadeh A, Safari F, Golmanesh L, Khoshbaten A. Acute effect of burn trauma to small skin areas on myocardial ischemiareperfusion injury in rat. *Physiology and Pharmacology*. 2011;15:241-248
374. Sheldrick A, Gray KM, Drew GM, Louttit JB. The effect of body temperature on myocardial protection conferred by ischaemic preconditioning or the selective adenosine a1 receptor agonist gr79236, in an anaesthetized rabbit model of myocardial ischaemia and reperfusion. *British journal of pharmacology*. 1999;128:385-395
375. Shen YT, Depre C, Yan L, Park JY, Tian B, Jain K, Chen L, Zhang Y, Kudej RK, Zhao X, Sadoshima J, Vatner DE, Vatner SF. Repetitive ischemia by coronary stenosis induces a novel window of ischemic preconditioning. *Circulation*. 2008;118:1961-1969
376. Shen B, Li J, Gao L, Zhang J, Yang B. Role of cc-chemokine receptor 5 on myocardial ischemia-reperfusion injury in rats. *Mol Cell Biochem*. 2013;378:137-144
377. Shinmura K, Tang XL, Wang Y, Xuan YT, Liu SQ, Takano H, Bhatnagar A, Bolli R. Cyclooxygenase-2 mediates the cardioprotective effects of the late phase of ischemic preconditioning in conscious rabbits. *Proc Natl Acad Sci U S A*. 2000;97:10197-10202
378. Shinmura K, Bolli R, Liu SQ, Tang XL, Kodani E, Xuan YT, Srivastava S, Bhatnagar A. Aldose reductase is an obligatory mediator of the late phase of ischemic preconditioning. *Circulation research*. 2002;91:240-246
379. Shintani Y, Node K, Asanuma H, Sanada S, Takashima S, Asano Y, Liao Y, Fujita M, Hirata A, Shinozaki Y, Fukushima T, Nagamachi Y, Okuda H, Kim J, Tomoike H, Hori M, Kitakaze M. Opening of ca2+-activated k+ channels is involved in ischemic preconditioning in canine hearts. *Journal of molecular and cellular cardiology*. 2004;37:1213-1218
380. Singh D, Sharma A, Singh M. Effect of actinomycin d and cycloheximide on ischemic preconditioning-induced delayed cardioprotective effect in rats. *Indian J Exp Biol*. 2000;38:982-987
381. Singh D, Chopra K. Evidence of the role of angiotensin at(1) receptors in remote renal preconditioning of myocardium. *Methods Find Exp Clin Pharmacol*. 2004;26:117-122
382. Sivarajah A, McDonald MC, Thiemermann C. The cardioprotective effects of preconditioning with endotoxin, but not ischemia, are abolished by a peroxisome proliferator-activated receptor-gamma antagonist. *J Pharmacol Exp Ther*. 2005;313:896-901
383. Sivarajah A, McDonald MC, Thiemermann C. The production of hydrogen sulfide limits myocardial ischemia and reperfusion injury and contributes to the cardioprotective effects of preconditioning with endotoxin, but not ischemia in the rat. *Shock*. 2006;26:154-161
384. Smits GJ, McVey M, Cox BF, Perrone MH, Clark KL. Cardioprotective effects of the novel adenosine a1/a2 receptor agonist amp 579 in a porcine model of myocardial infarction. *J Pharmacol Exp Ther*. 1998;286:611-618
385. Smul TM, Stumpner J, Blomeyer C, Lotz C, Redel A, Lange M, Roewer N, Kehl F. Propofol inhibits desflurane-induced preconditioning in rabbits. *J Cardiothorac Vasc Anesth*. 2011;25:276-281
386. Speechly-Dick ME, Mocanu MM, Yellon DM. Protein kinase c. Its role in ischemic preconditioning in the rat. *Circulation research*. 1994;75:586-590
387. Speechly-Dick ME, Baxter GF, Yellon DM. Ischaemic preconditioning protects hypertrophied myocardium. *Cardiovascular research*. 1994;28:1025-1029
388. Strohm C, Barancik T, Bruhl ML, Kilian SA, Schaper W. Inhibition of the er-kinase cascade by pd98059 and uo126 counteracts ischemic preconditioning in pig myocardium. *Journal of cardiovascular pharmacology*. 2000;36:218-229
389. Su DC, Chang ZW, Fan SY. [role of gap junction in ischemic preconditioning]. *Zhonghua Xin Xue Guan Bing Za Zhi*. 2006;34:690-694
390. Suzuki K, Miura T, Miki T, Tsuchida A, Shimamoto K. Infarct-size limitation by preconditioning is enhanced by dipyridamole administered before but not after preconditioning: Evidence for the role of interstitial adenosine level during preconditioning as a primary determinant of cardioprotection. *Journal of cardiovascular pharmacology*. 1998;31:1-9
391. Takano H, Manchikalapudi S, Tang XL, Qiu Y, Rizvi A, Jadoon AK, Zhang Q, Bolli R. Nitric oxide synthase is the mediator of late preconditioning against myocardial infarction in conscious rabbits. *Circulation*. 1998;98:441-449
392. Takano H, Tang XL, Qiu Y, Guo Y, French BA, Bolli R. Nitric oxide donors induce late preconditioning against myocardial stunning and infarction in conscious rabbits via an antioxidant-sensitive mechanism. *Circulation research*. 1998;83:73-84

393. Takano H, Tang XL, Bolli R. Differential role of k(atp) channels in late preconditioning against myocardial stunning and infarction in rabbits. *American journal of physiology. Heart and circulatory physiology*. 2000;279:H2350-2359
394. Takano H, Tang XL, Kodani E, Bolli R. Late preconditioning enhances recovery of myocardial function after infarction in conscious rabbits. *American journal of physiology. Heart and circulatory physiology*. 2000;279:H2372-2381
395. Takaoka A, Nakae I, Mitsunami K, Yabe T, Morikawa S, Inubushi T, Kinoshita M. Renal ischemia/reperfusion remotely improves myocardial energy metabolism during myocardial ischemia via adenosine receptors in rabbits: Effects of "remote preconditioning". *J Am Coll Cardiol*. 1999;33:556-564
396. Takasaki Y, Adachi N, Dote K, Tsubota S, Yorozuya T, Arai T. Ischemic preconditioning suppresses the noradrenaline turnover in the rat heart. *Cardiovascular research*. 1998;39:373-380
397. Talukder MA, Yang F, Shimokawa H, Zweier JL. Enos is required for acute in vivo ischemic preconditioning of the heart: Effects of ischemic duration and sex. *American journal of physiology. Heart and circulatory physiology*. 2010;299:4
398. Tanaka M, Fujiwara H, Yamasaki K, Miyamae M, Yokota R, Hasegawa K, Fujiwara T, Sasayama S. Ischemic preconditioning elevates cardiac stress protein but does not limit infarct size 24 or 48 h later in rabbits. *The American journal of physiology*. 1994;267:H1476-1482
399. Tanaka M, Fujiwara H, Yamasaki K, Sasayama S. Superoxide dismutase and n-2-mercaptopropionyl glycine attenuate infarct size limitation effect of ischaemic preconditioning in the rabbit. *Cardiovascular research*. 1994;28:980-986
400. Tang XL, Xuan YT, Zhu Y, Shirk G, Bolli R. Nicorandil induces late preconditioning against myocardial infarction in conscious rabbits. *American journal of physiology. Heart and circulatory physiology*. 2004;286:18
401. Tang XL, Takano H, Xuan YT, Sato H, Kodani E, Dawn B, Zhu Y, Shirk G, Wu WJ, Bolli R. Hypercholesterolemia abrogates late preconditioning via a tetrahydrobiopterin-dependent mechanism in conscious rabbits. *Circulation*. 2005;112:2149-2156
402. Tang XL, Sato H, Tiwari S, Dawn B, Bi Q, Li Q, Shirk G, Bolli R. Cardioprotection by postconditioning in conscious rats is limited to coronary occlusions <45 min. *American journal of physiology. Heart and circulatory physiology*. 2006;291:30
403. Tanhehco EJ, Yasojima K, McGeer PL, McGeer EG, Lucchesi BR. Preconditioning reduces myocardial complement gene expression in vivo. *American journal of physiology. Heart and circulatory physiology*. 2000;279:H1157-1165
404. Tanno M, Tsuchida A, Nozawa Y, Matsumoto T, Hasegawa T, Miura T, Shimamoto K. Roles of tyrosine kinase and protein kinase c in infarct size limitation by repetitive ischemic preconditioning in the rat. *Journal of cardiovascular pharmacology*. 2000;35:345-352
405. Thornton J, Striplin S, Liu GS, Swafford A, Stanley AW, Van Winkle DM, Downey JM. Inhibition of protein synthesis does not block myocardial protection afforded by preconditioning. *The American journal of physiology*. 1990;259:H1822-1825
406. Thornton JD, Liu GS, Olsson RA, Downey JM. Intravenous pretreatment with a1-selective adenosine analogues protects the heart against infarction. *Circulation*. 1992;85:659-665
407. Thornton JD, Daly JF, Cohen MV, Yang XM, Downey JM. Catecholamines can induce adenosine receptor-mediated protection of the myocardium but do not participate in ischemic preconditioning in the rabbit. *Circulation research*. 1993;73:649-655
408. Thornton JD, Liu GS, Downey JM. Pretreatment with pertussis toxin blocks the protective effects of preconditioning: Evidence for a g-protein mechanism. *Journal of molecular and cellular cardiology*. 1993;25:311-320
409. Thornton JD, Thornton CS, Downey JM. Effect of adenosine receptor blockade: Preventing protective preconditioning depends on time of initiation. *The American journal of physiology*. 1993;265:H504-508
410. Tissier R, Souktani R, Parent de Curzon O, Lellouche N, Henry P, Giudicelli JF, Berdeaux A, Ghaleh B. Pharmacological delayed preconditioning against ischaemia-induced ventricular arrhythmias: Effect of an adenosine a(1)-receptor agonist. *British journal of pharmacology*. 2001;134:1532-1538
411. Tissier R, Souktani R, Bruneval P, Giudicelli JF, Berdeaux A, Ghaleh B. Adenosine a(1)-receptor induced late preconditioning and myocardial infarction: Reperfusion duration is critical. *American journal of physiology. Heart and circulatory physiology*. 2002;283:H38-43
412. Toller WG, Kersten JR, Pagel PS, Hettrick DA, Warltier DC. Sevoflurane reduces myocardial infarct size and decreases the time threshold for ischemic preconditioning in dogs. *Anesthesiology*. 1999;91:1437-1446

413. Toombs CF, Wiltse AL, Shebuski RJ. Ischemic preconditioning fails to limit infarct size in reserpinized rabbit myocardium. Implication of norepinephrine release in the preconditioning effect. *Circulation*. 1993;88:2351-2358
414. Toufektsian MC, Morel S, Tanguy S, Jeunet A, de Leiris J, Boucher F. Involvement of reactive oxygen species in cardiac preconditioning in rats. *Antioxidants & redox signaling*. 2003;5:115-122
415. Toyoda Y, Di Gregorio V, Parker RA, Levitsky S, McCully JD. Anti-stunning and anti-infarct effects of adenosine-enhanced ischemic preconditioning. *Circulation*. 2000;102:III326-331
416. Tranter M, Ren X, Forde T, Wilhide ME, Chen J, Sartor MA, Medvedovic M, Jones WK. Nf-kb driven cardioprotective gene programs; hsp70.3 and cardioprotection after late ischemic preconditioning. *Journal of molecular and cellular cardiology*. 2010;49:664-672
417. Tsovolas K, Iliodromitis EK, Andreadou I, Zoga A, Demopoulou M, Iliodromitis KE, Manolaki T, Markantonis SL, Kremastinos DT. Acute administration of vitamin c abrogates protection from ischemic preconditioning in rabbits. *Pharmacol Res*. 2008;57:283-289
418. Tsuchida A, Miura T, Miki T, Shimamoto K, Iimura O. Role of adenosine receptor activation in myocardial infarct size limitation by ischaemic preconditioning. *Cardiovascular research*. 1992;26:456-461
419. Tsuchida A, Thompson R, Olsson RA, Downey JM. The anti-infarct effect of an adenosine a1-selective agonist is diminished after prolonged infusion as is the cardioprotective effect of ischaemic preconditioning in rabbit heart. *Journal of molecular and cellular cardiology*. 1994;26:303-311
420. Tsuchida A, Yang XM, Burckhartt B, Mullane KM, Cohen MV, Downey JM. Acadesine extends the window of protection afforded by ischaemic preconditioning. *Cardiovascular research*. 1994;28:379-383
421. Tsuchida A, Miura T, Tanno M, Nozawa Y, Kita H, Shimamoto K. Time window for the contribution of the delta-opioid receptor to cardioprotection by ischemic preconditioning in the rat heart. *Cardiovasc Drugs Ther*. 1998;12:365-373
422. Turan NN, Basgut B, Aypar E, Ark M, Iskit AB, Cakici I. Chemical preconditioning effect of 3-nitropropionic acid in anesthetized rat heart. *Life Sci*. 2008;82:928-933
423. Ueda Y, Kitakaze M, Komamura K, Minamino T, Asanuma H, Sato H, Kuzuya T, Takeda H, Hori M. Pravastatin restored the infarct size-limiting effect of ischemic preconditioning blunted by hypercholesterolemia in the rabbit model of myocardial infarction. *J Am Coll Cardiol*. 1999;34:2120-2125
424. Uematsu M, Gaudette GR, Laurikka JO, Levitsky S, McCully JD. Adenosine-enhanced ischemic preconditioning decreases infarct in the regional ischemic sheep heart. *Ann Thorac Surg*. 1998;66:382-387
425. Valtchanova-Matchouganska A, Gondwe M, Nadar A. The role of c-reactive protein in ischemia/reperfusion injury and preconditioning in a rat model of myocardial infarction. *Life Sci*. 2004;75:901-910
426. Valtchanova-Matchouganska A, Ojewole JA. Mechanisms of opioid delta (delta) and kappa (kappa) receptors' cardioprotection in ischaemic preconditioning in a rat model of myocardial infarction. *Cardiovasc J S Afr*. 2003;14:73-80
427. Van Den Doel MA, Gho BCG, Duval SY, Schoemaker RG, Duncker DJ, Verdouw PD. Hypothermia extends the cardioprotection by ischaemic preconditioning to coronary artery occlusions of longer duration. *Cardiovascular research*. 1998;37:76-81
428. Vander Heide RS, Schwartz LM, Reimer KA. The novel calcium antagonist ro 40-5967 limits myocardial infarct size in the dog. *Cardiovascular research*. 1994;28:1526-1532
429. Van Winkle DM, Chien GL, Wolff RA, Soifer BE, Kuzume K, Davis RF. Cardioprotection provided by adenosine receptor activation is abolished by blockade of the katp channel. *The American journal of physiology*. 1994;266:H829-839
430. Van Wylen DG. Effect of ischemic preconditioning on interstitial purine metabolite and lactate accumulation during myocardial ischemia. *Circulation*. 1994;89:2283-2289
431. Vetterlein F, Muhlfeld C, Cetegen C, Volkmann R, Schrader C, Hellige G. Redistribution of connexin43 in regional acute ischemic myocardium: Influence of ischemic preconditioning. *American journal of physiology. Heart and circulatory physiology*. 2006;291:24
432. Vincent A, Gahide G, Sportouch-Dukhan C, Covinhes A, Franck-Miclo A, Roubille F, Barrere C, Adda J, Dantec C, Redt-Clouet C, Piot C, Nargeot J, Barrere-Lemaire S. Down-regulation of the transcription factor zac1 upon pre- and postconditioning protects against i/r injury in the mouse myocardium. *Cardiovascular research*. 2012;16:16
433. Virag JA, Anderson EJ, Kent SD, Blanton HD, Johnson TL, Moukdar F, DeAntonio JH, Thayne K, Ding JM, Lust RM. Cardioprotection via preserved mitochondrial structure and function in the mper2-mutant mouse myocardium. *American journal of physiology. Heart and circulatory physiology*. 2013;305:H477-483
434. Vladic N, Ge ZD, Leucker T, Brzezinska AK, Du JH, Shi Y, Warltier DC, Pratt PF, Jr., Kersten JR. Decreased tetrahydrobiopterin and disrupted association of hsp90 with enos by hyperglycemia impair myocardial ischemic preconditioning. *American journal of physiology. Heart and circulatory physiology*. 2011;301:9

435. Vogt AM, Ando H, Arras M, Elsasser A. Lack of adenosine causes myocardial refractoriness. *J Am Coll Cardiol*. 1998;31:1134-1141
436. Vogt AM, Ackermann C, Yildiz M, Schoels W, Kubler W. Lactate accumulation rather than atp depletion predicts ischemic myocardial necrosis: Implications for the development of lethal myocardial injury. *Biochim Biophys Acta*. 2001;16:219-226
437. Wall TM, Sheehy R, Hartman JC. Role of bradykinin in myocardial preconditioning. *J Pharmacol Exp Ther*. 1994;270:681-689
438. Walsh RS, Tsuchida A, Daly JJ, Thornton JD, Cohen MV, Downey JM. Ketamine-xylazine anaesthesia permits a katp channel antagonist to attenuate preconditioning in rabbit myocardium. *Cardiovascular research*. 1994;28:1337-1341
439. Wang Y, Xu H, Mizoguchi K, Oe M, Maeta H. Intestinal ischemia induces late preconditioning against myocardial infarction: A role for inducible nitric oxide synthase. *Cardiovascular research*. 2001;49:391-398
440. Wang YP, Maeta H, Mizoguchi K, Suzuki T, Yamashita Y, Oe M. Intestinal ischemia preconditions myocardium: Role of protein kinase c and mitochondrial k(atp) channel. *Cardiovascular research*. 2002;55:576-582
441. Wang NF, Xu J, Xia Q, Gao Q, Shi XX, Zhou ZL, Wang LL, Tong GX, Li H, Li PZ, Zhang X, Ling F, Pan H, Yang JM. [mechanism of cardioprotection induced by noninvasive limb ischemic preconditioning]. *Zhonghua Yi Xue Za Zhi*. 2009;89:1999-2002
442. Wang S, Dusting GJ, May CN, Woodman OL. 3',4'-dihydroxyflavonol reduces infarct size and injury associated with myocardial ischaemia and reperfusion in sheep. *British journal of pharmacology*. 2004;142:443-452
443. Wang Y, Kodani E, Wang J, Zhang SX, Takano H, Tang XL, Bolli R. Cardioprotection during the final stage of the late phase of ischemic preconditioning is mediated by neuronal no synthase in concert with cyclooxygenase-2. *Circulation research*. 2004;95:84-91
444. Watanabe K, Yaoita H, Ogawa K, Oikawa M, Maehara K, Maruyama Y. Attenuated cardioprotection by ischemic preconditioning in coronary stenosed heart and its restoration by carvedilol. *Cardiovascular research*. 2006;71:537-547
445. Weber NC, Toma O, Wolter JI, Obal D, Mullenheim J, Preckel B, Schlack W. The noble gas xenon induces pharmacological preconditioning in the rat heart in vivo via induction of pkc- and p38 mapk. *British journal of pharmacology*. 2005;144:123-132
446. Weber NC, Frassdorf J, Ratajczak C, Grueber Y, Schlack W, Hollmann MW, Preckel B. Xenon induces late cardiac preconditioning in vivo: A role for cyclooxygenase 2? *Anesth Analg*. 2008;107:1807-1813
447. Weinbrenner C, Nelles M, Herzog N, Sarvary L, Strasser RH. Remote preconditioning by infrarenal occlusion of the aorta protects the heart from infarction: A newly identified non-neuronal but pkc-dependent pathway. *Cardiovascular research*. 2002;55:590-601
448. Weinbrenner C, Schulze F, Sarvary L, Strasser RH. Remote preconditioning by infrarenal aortic occlusion is operative via delta1-opioid receptors and free radicals in vivo in the rat heart. *Cardiovascular research*. 2004;61:591-599
449. Wolfe CL, Sievers RE, Visseren FL, Donnelly TJ. Loss of myocardial protection after preconditioning correlates with the time course of glycogen recovery within the preconditioned segment. *Circulation*. 1993;87:881-892
450. Wolfrum S, Schneider K, Heidbreder M, Nienstedt J, Dominiak P, Dendorfer A. Remote preconditioning protects the heart by activating myocardial pkcepsilon-isoform. *Cardiovascular research*. 2002;55:583-589
451. Wolfrum S, Nienstedt J, Heidbreder M, Schneider K, Dominiak P, Dendorfer A. Calcitonin gene related peptide mediates cardioprotection by remote preconditioning. *Regul Pept*. 2005;127:217-224
452. Wong GT, Ling Ling J, Irwin MG. Activation of central opioid receptors induces cardioprotection against ischemia-reperfusion injury. *Anesth Analg*. 2010;111:24-28
453. Wong GT, Lu Y, Mei B, Xia Z, Irwin MG. Cardioprotection from remote preconditioning involves spinal opioid receptor activation. *Life Sci*. 2012;91:860-865
454. Wu DJ, Minatoguchi S, Uno Y, Arai M, Wang N, Nishida Y, Hashimoto K, Hashimoto Y, Takemura G, Fujiwara T, Fujiwara H. Combination of n-methyl-1-deoxynojirimycin and ischemic preconditioning markedly reduces the size of myocardial infarcts in rabbits. *Jpn Circ J*. 2001;65:673-677
455. Wu YN, Zhang Z, Chen Y, Jiao JJ, Kang Y, Gao WZ, Lou JS, Liu YX. Study on cardioprotection of ramipril against ischemia/reperfusion injury in diabetic rats. [chinese]. *Chinese Pharmacological Bulletin*. 2008;24:924-928
456. Wu YN, Zhu XH, Yuan HJ, Yu H, Li SJ, Kang Y, Jiao JJ, Gao WZ, Liu YX, Lou JS. Noninvasive limb ischemic preconditioning increased antioxidative ability of myocardium after ischemia-reperfusion injury in rats. [chinese]. *Chinese Pharmacological Bulletin*. 2008;24:1049-1052

457. Wu YN, Chen Y, Zhang Z, Dong YL, Yu XK, Jiao JJ, Kang Y, Gao WZ, Lou JS, Liu YX. [study on ultrastructure of cardioprotection of ramipril against ischemia/reperfusion injury in diabetic rats]. *Zhongguo Ying Yong Sheng Li Xue Za Zhi*. 2009;25:485-489
458. Wu YN, Yu H, Zhu XH, Yuan HJ, Kang Y, Jiao JJ, Gao WZ, Liu YX, Lou JS. Noninvasive delayed limb ischemic preconditioning attenuates myocardial ischemia-reperfusion injury in rats by a mitochondrial k(atp) channel-dependent mechanism. *Physiol Res*. 2011;60:271-279
459. Wu FY, Hsiao CY, Ou-Yang CP, Lai CC, Wang YY, Weng ZC, Huang CH. Preconditioning threshold of brief pressure overload of the left ventricle. *Journal of the Chinese Medical Association : JCMA*. 2013;76:497-503
460. Xiang SY, Ye LL, Duan LL, Liu LH, Ge ZD, Auchampach JA, Gross GJ, Duan DD. Characterization of a critical role for cftr chloride channels in cardioprotection against ischemia/reperfusion injury. *Acta Pharmacol Sin*. 2011;32:824-833
461. Xiao L, Lu R, Hu CP, Deng HW, Li YJ. Delayed cardioprotection by intestinal preconditioning is mediated by calcitonin gene-related peptide. *European journal of pharmacology*. 2001;427:131-135
462. Xiong J, Wang Q, Xue FS, Yuan YJ, Li S, Liu JH, Liao X, Zhang YM. Comparison of cardioprotective and anti-inflammatory effects of ischemia pre- and postconditioning in rats with myocardial ischemia-reperfusion injury. *Inflamm Res*. 2011;60:547-554
463. Xu JM, Chang YT, Hu DX. [protective effects of sevoflurane pretreatment on myocardial ischemia-reperfusion injury in rabbits]. *Hunan Yi Ke Da Xue Xue Bao*. 2001;26:79-82
464. Xu Z, Jiao Z, Cohen MV, Downey JM. Protection from amp 579 can be added to that from either cariporide or ischemic preconditioning in ischemic rabbit heart. *Journal of cardiovascular pharmacology*. 2002;40:510-518
465. Xu HS, Chen SX, Luo WJ, Gao QA, Yang Y. [effect of cortisol on ischemic preconditioning in rabbit hearts]. *Zhong Nan Da Xue Xue Bao Yi Xue Ban*. 2006;31:667-670
466. Xu JF, Wang ZQ, Wang JP. Ferilnic nirate produces delayed preconditioning against myocardial ischemia and reperfusion injury in rats. *Arch Pharm Res*. 2010;33:881-887
467. Xu H, Chen S, Luo W, Jiang H. [role of stress in myocardial protection of ischemic preconditioning]. *Zhong Nan Da Xue Xue Bao Yi Xue Ban*. 2013;38:804-808
468. Xuan YT, Guo Y, Zhu Y, Wang OL, Rokosh G, Bolli R. Endothelial nitric oxide synthase plays an obligatory role in the late phase of ischemic preconditioning by activating the protein kinase c epsilon p44/42 mitogen-activated protein kinase pser-signal transducers and activators of transcription1/3 pathway. *Circulation*. 2007;116:535-544
469. Yada T, Shimokawa H, Kajiya F. Cardioprotective effect of hydroxyfasudil as a specific rho-kinase inhibitor, on ischemia-reperfusion injury in canine coronary microvessels in vivo. *Clin Hemorheol Microcirc*. 2006;34:177-183
470. Yamada K, Matsui K, Yamamoto S, Kitano M, Ohashi N. Effect of an na+/h+ exchange inhibitor, sm-20550, on ischemic preconditioning in rabbits. *Int J Tissue React*. 2002;24:1-10
471. Yamasaki K, Fujiwara H, Tanaka M, Yokota R, Miyamae M, Ogawa J, Fujiwara T, Sasayama S. Preconditioning with 15-minute ischemia extends myocardial infarct size after subsequent 30-minute ischemia in rabbits. *Jpn Circ J*. 1997;61:344-352
472. Yamasaki K, Tanaka M, Yokota R, Fujiwara H, Sasayama S. Dilazep dihydrochloride prolongs the infarct size-limiting effect of ischemic preconditioning in rabbits. *Heart Vessels*. 2000;15:227-232
473. Yamashita N, Hoshida S, Taniguchi N, Kuzuya T, Hori M. A "second window of protection" occurs 24 h after ischemic preconditioning in the rat heart. *Journal of molecular and cellular cardiology*. 1998;30:1181-1189
474. Yamashita N, Hoshida S, Otsu K, Taniguchi N, Kuzuya T, Hori M. The involvement of cytokines in the second window of ischaemic preconditioning. *British journal of pharmacology*. 2000;131:415-422
475. Yang XM, Arnoult S, Tsuchida A, Cope D, Thornton JD, Daly JF, Cohen MV, Downey JM. The protection of ischaemic preconditioning can be reinstated in the rabbit heart after the initial protection has waned. *Cardiovascular research*. 1993;27:556-558
476. Yang XM, Baxter GF, Heads RJ, Yellon DM, Downey JM, Cohen MV. Infarct limitation of the second window of protection in a conscious rabbit model. *Cardiovascular research*. 1996;31:777-783
477. Yang YJ, Zhao JL, You SJ, Wu YJ, Jing ZC, Yang WX, Meng L, Wang YW, Gao RL. Different effects of tirofiban and aspirin plus clopidogrel on myocardial no-reflow in a mini-swine model of acute myocardial infarction and reperfusion. *Heart*. 2006;92:1131-1137
478. Yang XM, Liu Y, Tandon N, Kambayashi J, Downey JM, Cohen MV. Attenuation of infarction in cynomolgus monkeys: Preconditioning and postconditioning. *Basic research in cardiology*. 2010;105:119-128
479. Yang J, Li Y, Hu C. Ischemic preconditioning protects against myocardial ischemia-reperfusion injury through inhibiting toll-like receptor 4/nf-kappab signaling pathway in rats. *Zhong Nan Da Xue Xue Bao Yi Xue Ban*. 2011;36:972-978

480. Yang XM, Cui L, Alhammouri A, Downey JM, Cohen MV. Triple therapy greatly increases myocardial salvage during ischemia/reperfusion in the in situ rat heart. *Cardiovasc Drugs Ther*. 2013;27:403-412
481. Yang Z, Tian Y, Liu Y, Hennessy S, Kron IL, French BA. Acute hyperglycemia abolishes ischemic preconditioning by inhibiting akt phosphorylation: Normalizing blood glucose before ischemia restores ischemic preconditioning. *Oxidative medicine and cellular longevity*. 2013;2013:329183
482. Yao Z, Gross GJ. Role of nitric oxide, muscarinic receptors, and the atp-sensitive k+ channel in mediating the effects of acetylcholine to mimic preconditioning in dogs. *Circulation research*. 1993;73:1193-1201
483. Yao Z, Gross GJ. Acetylcholine mimics ischemic preconditioning via a glibenclamide-sensitive mechanism in dogs. *The American journal of physiology*. 1993;264:H2221-2225
484. Yao Z, Gross GJ. A comparison of adenosine-induced cardioprotection and ischemic preconditioning in dogs. Efficacy, time course, and role of katp channels. *Circulation*. 1994;89:1229-1236
485. Yao Z, Gross GJ. Activation of atp-sensitive potassium channels lowers threshold for ischemic preconditioning in dogs. *The American journal of physiology*. 1994;267:H1888-1894
486. Yao Z, Mizumura T, Mei DA, Gross GJ. Katp channels and memory of ischemic preconditioning in dogs: Synergism between adenosine and katp channels. *The American journal of physiology*. 1997;272:H334-342
487. Yellon DM, Alkhulaifi AM, Browne EE, Pugsley WB. Ischaemic preconditioning limits infarct size in the rat heart. *Cardiovascular research*. 1992;26:983-987
488. Yorozuya T, Adachi N, Dote K, Nakanishi K, Takasaki Y, Arai T. Enhancement of na+,k(+)-atpase and ca(2+)-atpase activities in multi-cycle ischemic preconditioning in rabbit hearts. *Eur J Cardiothorac Surg*. 2004;26:981-987
489. Ytrehus K, Liu Y, Downey JM. Preconditioning protects ischemic rabbit heart by protein kinase c activation. *The American journal of physiology*. 1994;266:H1145-1152
490. Yu CK, Li YH, Wong GT, Wong TM, Irwin MG. Remifentanil preconditioning confers delayed cardioprotection in the rat. *Br J Anaesth*. 2007;99:632-638
491. Yu M, Wen N, Wenzhong Z, Yuanchang X, Xiaoming D, Yongjin L. Effect of repeated ischaemic preconditioning on tlr4 and proinflammatory cytokines tnf-alpha and il-1beta in myocardial ischaemia-reperfusion injury in a rat model. *Arch Med Sci*. 2010;6:843-847
492. Yu GG, Zeng XJ, Wang HX, Lu LQ, Zheng SP, Ma LQ, Chang J, Wang J, Zhang DM, Du FH, Zhang LK. Cytochrome p450 2j3/epoxyeicosatrienoic acids mediate the cardioprotection induced by ischaemic post-conditioning, but not preconditioning, in the rat. *Clin Exp Pharmacol Physiol*. 2011;38:63-70
493. Zacharowski K, Zacharowski PA, Friedl P, Mastan P, Koch A, Boehm O, Rother RP, Reingruber S, Henning R, Emeis JJ, Petzelbauer P. The effects of the fibrin-derived peptide bbeta(15-42) in acute and chronic rodent models of myocardial ischemia-reperfusion. *Shock*. 2007;27:631-637
494. Zhang Y, Irwin MG, Wong TM. Remifentanil preconditioning protects against ischemic injury in the intact rat heart. *Anesthesiology*. 2004;101:918-923
495. Zhang J, Bian HJ, Li XX, Liu XB, Sun JP, Li N, Zhang Y, Ji XP. Erk-mapk signaling opposes rho-kinase to reduce cardiomyocyte apoptosis in heart ischemic preconditioning. *Mol Med*. 2010;16:307-315
496. Zhang SZ, Wang NF, Xu J, Gao Q, Lin GH, Bruce IC, Xia Q. Kappa-opioid receptors mediate cardioprotection by remote preconditioning. *Anesthesiology*. 2006;105:550-556
497. Zhang XH, Li CM, Ma XJ, Luo M. [correlation of limb and myocardial ischemia postconditioning with acute myocardial reperfusion injury]. *Zhonghua Yi Xue Za Zhi*. 2006;86:841-845
498. Zhao ZQ, Corvera JS, Halkos ME, Kerendi F, Wang NP, Guyton RA, Vinten-Johansen J. Inhibition of myocardial injury by ischemic postconditioning during reperfusion: Comparison with ischemic preconditioning. *American journal of physiology. Heart and circulatory physiology*. 2003;285:H579-588
499. Zhao X, Park J, Ho D, Gao S, Yan L, Ge H, Ilsmaa S, Lin L, Tian B, Vatner DE, Graham RM, Vatner SF. Cardiomyocyte overexpression of the alpha1a-adrenergic receptor in the rat phenocopies second but not first window preconditioning. *American journal of physiology. Heart and circulatory physiology*. 2012;3:3
500. Zhu X, Liu B, Zhou S, Chen YR, Deng Y, Zweier JL, He G. Ischemic preconditioning prevents in vivo hyperoxygenation in postischemic myocardium with preservation of mitochondrial oxygen consumption. *American journal of physiology. Heart and circulatory physiology*. 2007;293:18
501. Zhu XH, Li YM, Yuan HJ, Luo Q, Wu YN, Kang Y, Jiao JJ, Gao WZ, Liu YX, Lou JS. Noninvasive limb ischemic preconditioning increased antioxidative ability of myocardium after ischemia-reperfusion injury in diabetic rats. [chinese]. *Chinese Pharmacological Bulletin*. 2009;25:239-243
502. Zhu XH, Yuan HJ, Wu YN, Kang Y, Jiao JJ, Gao WZ, Liu YX, Lou JS, Xia Z. Non-invasive limb ischemic pre-conditioning reduces oxidative stress and attenuates myocardium ischemia-reperfusion injury in diabetic rats. *Free Radic Res*. 2011;45:201-210

503. Zhu SB, Liu Y, Zhu Y, Yin GL, Wang RP, Zhang Y, Zhu J, Jiang W. Remote preconditioning, perconditioning, and postconditioning: A comparative study of their cardio-protective properties in rat models. *Clinics*. 2013;68:263-268
